# Metabolic interactions between bacterial co-isolates from catheter-associated urinary tract infections

**DOI:** 10.1038/s41598-025-33855-1

**Published:** 2026-01-14

**Authors:** Dmytro Sokol, Olena Rzhepishevska, Iryna Marynova, Tor Monsen, Henrik Antti, Madeleine Ramstedt

**Affiliations:** 1 Department of Chemistry, Umeå Centre of Microbial Research, Umeå University, Umeå, Sweden; 2 Odesa I. I. Mechnikov National University, Odesa, Ukraine; 3 Department of Clinical Microbiology, Umeå University, Umeå, Sweden

**Keywords:** Diseases, Microbiology

## Abstract

Catheter-associated urinary tract infections (CAUTI) are complex infections often involving multi-species bacteria. *Escherichia coli* is frequently an early coloniser. Subsequent colonisation by *Pseudomonas aeruginosa* and coexistence mechanisms between the two strains within urethral catheters is not yet fully understood. In this study, metabolic adaptations between co-isolated clinical *E. coli* and *P. aeruginosa* strains were investigated. It was found that *P. aeruginosa* outgrew *E. coli* in artificial urine medium (AUM), whereas *E. coli* dominated in culture broth such as Iso-sensitest. No evidence of direct antagonism was observed. Metabolite analyses revealed distinct metabolite patterns indicating cross-feeding and metabolic adaptations. In AUM, stress-response metabolites were elevated. Additionally, *E. coli* appeared to experience Fe-limitation in AUM, while the same was not observed for *P. aeruginosa.* The results highlight the influence of nutrient conditions on processes within mixed biofilms.

**Supplementary Information:**

The online version contains supplementary material available at 10.1038/s41598-025-33855-1.

## Introduction

Urinary tract infections (UTIs) are among the most common infections globally^1^, associated with high morbidity and mortality and a high economic burden on the healthcare system as individuals^2–4^. UTI engage the urinary tract system involving urethra, bladder, kidney and prostate, of which bladder infection (cystitis) is the most common. Globally, UTI is estimated to be responsible for 405 million infections and 237 000 deaths in 2019^2^. In the USA, UTIs lead to about 3.5 million ambulance and 2.9 million emergency department visits with annual hospital cost estimated at 6 billion USD^4,5^. All types of UTI can lead to life-threatening systemic infections, with UTI-associated sepsis contributing up to 25% of all sepsis cases^6^. Risk factors are gender (women x 3.6), age, diabetes, urinary anatomical abnormalities and urinary stones. Also, recurrent- or UTI relapses are common after UTIs^4^ with symptoms such as urinary dysuria, frequency, urgency, suprapubic and loin pain. The spontaneous cure rate among uncomplicated community-acquired UTIs of symptoms and bacteriuria is relatively good^7^. In complicated UTIs, the risk of adverse events is higher, for example, due to the presence of medical devices such as urinary catheters or other complicating factors^4,8^. The most common uropathogen is *Escherichia coli*, which is responsible for approximately 70% of UTIs. Other microorganisms are *Staphylococcus saprophyticus*, *Klebsiella pneumoniae*,* Proteus mirabilis*,* Pseudomonas aeruginosa*,* Enterococcus* spp., *Streptococci* group B^7,9^. Many of these uropathogens may be resistant to antimicrobials, contributing to worsened patient outcomes and even death^9^. Antimicrobial treatment of UTIs is common and done with different strategies and success^10^. Due to the large population of UTI patients treated with antimicrobials, there is a major risk for increased antimicrobial resistance (AMR) among uropathogens, accelerating further dissemination of AMR isolates to other areas of society. In addition to resistance, other factors may complicate the infection, such as the ability of the microorganisms to form coatings called biofilms. The biofilms consist of microbial communities, often attached to a surface, producing extracellular polymeric substances (EPS) that encapsulate them. This can function both to increase the adhesion to various surfaces and to protect bacteria from a harsh environment^11–13^. The biofilm matrix can consist of both substances produced by bacteria and substances derived from their surroundings, which form a microenvironment where bacteria can multiply, engage in complex interactions using signalling molecules and exchange metabolites^11,14–16^. These interactions can occur between bacteria of the same species, in mono-species biofilms, as well as in multispecies biofilms^17^, increasing fitness of the microbial community^18–20^. It has been well described that multispecies bacterial communities can survive better than mono-species biofilms under many types of stress factors^21^, including antibiotic exposure^16,22^. Thus, biofilms play a large role in bacterial survival^15,16,18^.

UTIs linked to biofilm formation often relate to the presence of foreign bodies in the urinary tract, such as urinary catheters. Colonisation of indwelling bladder catheters increases the risk that bacteriuria develops into UTI by 10% each day^23^. Urinary catheters are used in very large numbers within healthcare and in the care of older people in nursing homes. A market estimate from 2001 described that more than 30 million urinary catheters were used in the US every year^24^. Catheter-associated urinary tract infections (CAUTIs) are especially challenging to treat due to the protective effect that the biofilm provides for its inhabitants against antibiotics and the host immune system. CAUTIs are caused by bacteria (or fungi) that colonise the surfaces of the urinary catheter within a week after insertion, forming biofilms^25,26^. A further complication arises in long-term catheterised patients where the catheter may become blocked by crystallised salts from the urine on the catheter surface and in the biofilm. The biofilms forming in catheters often develop into multispecies biofilms^27,28^ containing bacteria such as *Pseudomonas aeruginosa* and *Proteus mirabilis* possessing urease. This degrades urea into ammonium and, thereby, increases urine pH, inducng the formation of crystalline biofilms blocking the catheter. To better understand the interactions between bacteria in biofilm processes, there is a need to understand how bacteria co-exist and how this influences biofilm formation in clinical settings^16,25,27–30^.

In this context, metabolic interactions represent one important perspective to study. Bacteria may benefit from co-existing inside the catheter by metabolising different nutrients and/or metabolites secreted by other bacteria in proximity^14,15^. It has previously been described that cross-feeding increases fitness for bacteria as it increases access to new metabolic pathways that may be lacking in one bacterium, enhances the utilisation of resources by recycling waste products formed, and enables specialisation of metabolism^17^^,^^31^. In gut-associated consortia, for example, cross-feeding and other polymicrobial metabolic interactions are crucial for the functional structure of the biofilm^32^. Thus, metabolic cross-feeding is assumed to be a pronounced feature within biofilms and has been suggested as a potential target for antibacterial treatments^17,33,34^. In medical contexts, it has been shown that metabolic cross-feeding allowed *P. aeruginosa* to reach higher growth yields by consuming degradation products from other bacteria that digest lung mucin^17^. Pathogenicity during periodontitis has also been described to increase as a consequence of metabolic cross-metabolism between species^17^. Thus, it is interesting to study if similar processes occur between clinical bacterial strains involved in CAUTIs, and what types of metabolic interactions may occur. Most previous metabolomic studies of UTI have focussed on analysing and identifying metabolites in body fluids such as urine or blood, with the aim of characterizing compositional changes and/or finding biomarkers for infection or sepsis^35–38^. It has been described that presence of bacteria give rise to increased amounts of polyamines that support bacterial functions in various stress conditions^36,38,39^. Furthermore, alterations in levels of amino acids and TCA intermediates have been described for mono-species systems^35,40^ in response to various stress conditions of relevance to the urinary tract.

This study focuses on *E. coli*, a common bacterium isolated from the urinary tract during early urinary tract infection^25^, and *P. aeruginosa* that has been reported to colonise the lower urinary tract at later stages, often after *E. coli* has settled^11,14,27,28,41^. This work, thereby contributes to the research of metabolic interactions by presenting data from well controlled studies of clinically relevant bacterial cultures as both monospecies and dual-species communities. The controlled conditions exclude patient induced environmental factors that may influence the bacterial response observed in the metabolite patterns. Wang et al. reviewed metabolomic studies on bacterial systems and described that environmental factors have a large influence on the production of metabolites in bacteria^42^. Thus, there is a need for both controlled in vitro lab studies and studies in more complex and variable patient derived body fluids. The controlled studies of bacterial isolates under well- defined conditions inform regarding adaptations in bacteria arising from specific environmental alterations, such as nutrient level, as well as highlight differences (or lack of) between clinical strains and species. These more fundamental studies can thereafter be used to shine light on clinical data from patient samples where the variability will inherently be much larger due to host factors, environmental factors as well as which pathogen (species and strain) that was infecting the host. Here, we aimed to study clinically relevant dual-species biofilms to investigate to what extent and by which mechanisms bacteria may benefit from co-habitation on medical devices. In this research, we have focused on studying interactions between pairs of clinical CAUTI isolates of *E. coli* and *P. aeruginosa* that have been co-isolated from the same patient. The choice of CAUTI pathogens was made based on the clinical material available to us, from bacterial species most commonly present and isolated. The strains in the pairs were grown both as monocultures and co-cultures in different nutrient conditions to trigger various types of metabolic interactions. Three nutrient media were used: two relatively high-nutrient media: Iso-sensitest (ISO) and Luria-Bertani broth (LB), and a more nutrient-limited artificial urine medium (AUM). The LB medium was included, in a selection of experiments, to enable comparisons with other studies in the literature. The difference in nutrient content was chosen to understand how this may influence the interactions between bacterial strains. Metabolite profiles were obtained using gas chromatography mass-spectrometry (GC-MS) analyses and multivariate statistical analysis^29,30,43^ in order to study which types of metabolic interactions may occur during co-habitation between clinical strains, depending on the nutrient availability.

## Results

Anonymized clinical isolates (abbreviated Tmp for *P. aeruginosa* and Tme for *E. coli*) and reference strains (*P. aeruginosa* PA01 and *E. coli* K12) may differ in phenotype even when originating from the same bacterial species. Thus, to characterise phenotype for the different isolates, we performed several assays investigating their metabolic activity, motility, aggregation behaviour, hydrophobicity, and zeta potential (Supplemental information Table S1 & Figure S1). The *P. aeruginosa* strains were generally more hydrophobic (7–44%) than the *E. coli* strains (3–16%), but showed lower aggregative behaviour in pure phosphate buffer solution compared to *E. coli* (Supplementary information Figure S1 A & B). The Tmp07 isolate was the most hydrophobic, followed by Tmp06 and Tmp05. Concerning zeta potential, Tmp04 had the most negative zeta potential (−43 ± 3 mV), and Tmp06 was the least negative (−21 ± 2 mV) of the *P. aeruginosa* strains (Supplementary information Figure S1C). The variation in zeta potential between *E. coli* isolates was smaller; Tme14 was the most negative (−35 ± 1 mV) and Tme15 the least negative (−28 ± 1 mV), but all clinical strains had a lower zeta potential than the *E. coli* K12 reference strain (−42 ± 1 mV). Differences in swimming were observed between the strains, where Tmp07 and PA01 had the largest swimming and Tmp05 and Tmp06 the lowest of the *P. aeruginosa* strains. For *E. coli*, Tme13 had the highest swimming, and no swimming was observed for Tme14. The *E. coli* showed somewhat higher twitching than *P. aeruginosa* (Supplementary information Figure S1). No swarming was detected for *E. coli*, and only Tmp07 and Tmp05 of the *P. aeruginosa* isolates exhibited swarming. Measurements of pH in AUM were performed to determine the alkalinisation from urease activity during bacterial growth. All CAUTI strains slightly increased the pH (ΔpH ≈ 0.2) of the AUM medium (10 g/L urea) despite clinical *E. coli* strains being urease negative. However, a larger (ΔpH ≈ 0.5) increase in pH was observed for single cultures of *P. aeruginosa* and co-cultures of *P. aeruginosa* with *E. coli* (Supplementary information Figure S2) that showed varying degrees of urease activity.

### Biofilm formation and viable counts

**Fig. 1:**
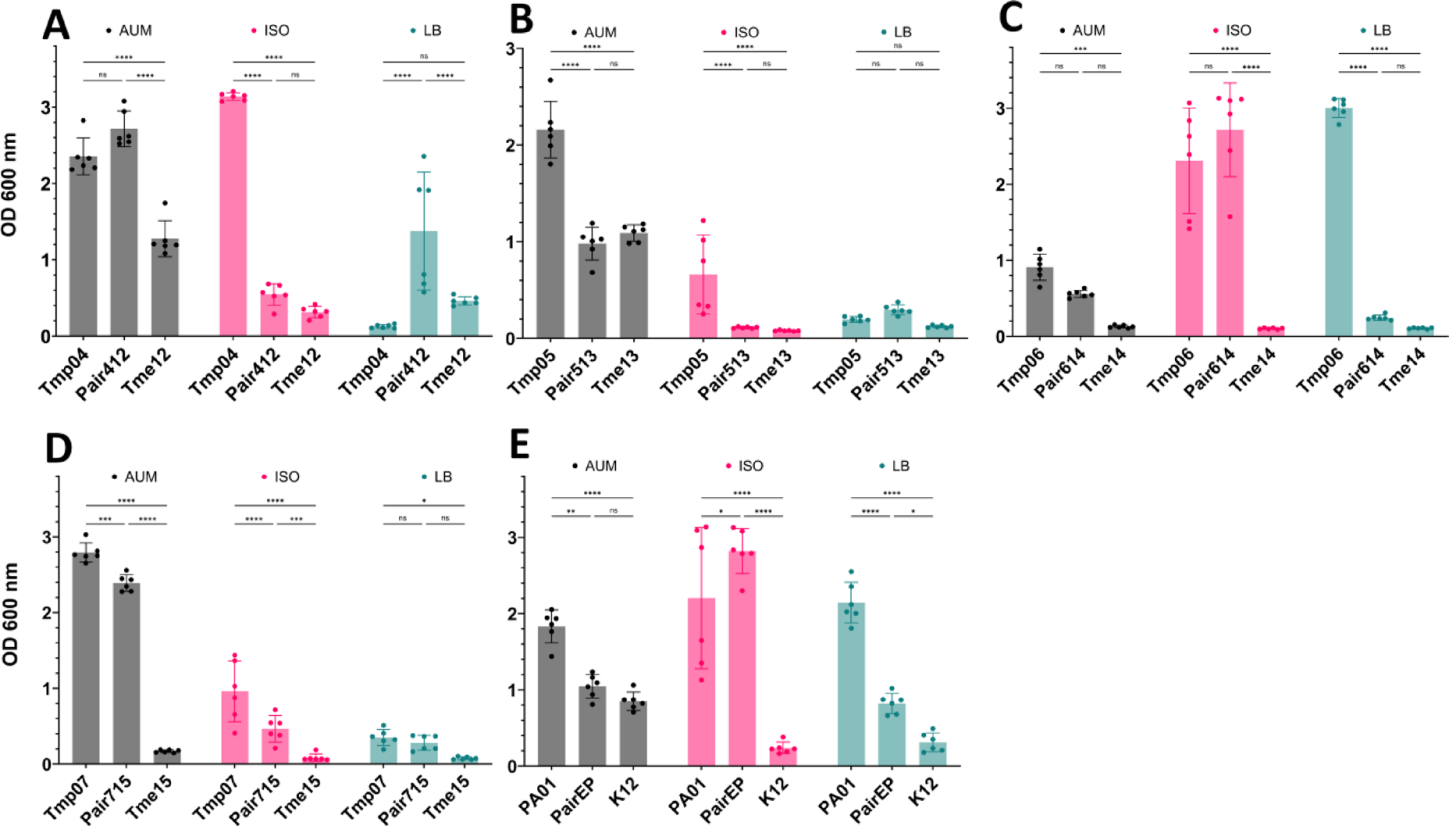
Biofilm formation of CAUTI-strains (mono- and co-cultures) in 3 media during 20 h growth. (**A**–**E**) Biofilm formation (OD600 nm, optical density at 600 nm wavelength) for the following strain combinations: (**A**) Tmp04/Tme12/Pair412, (**B**) Tmp05/Tme13/Pair513, (**C**) Tmp06/Tme14/Pair614, (**D**) Tmp07/Tme15/Pair715, and (**E**) PA01/K12/PairEP. Each panel displays *P. aeruginosa* monocultures (left bars), *E. coli* monocultures (right bars), and their co-cultures (middle bars), grown in AUM (black bars), ISO (red bars), and LB (green bars) media. Each bar represents the mean ± standard deviation from 6 biological replicates (n=6), with individual replicate values overlaid as dots. Within each medium group, significance bars indicate pairwise comparisons between strains determined using two-way ANOVA (factors: strain and medium) with Tukey’s post-hoc test. Statistical significance is represented as: ns (not significant), * p ≤ 0.05, ** p ≤ 0.01, *** p ≤ 0.001, **** p ≤ 0.0001.

The biofilm formation varied among isolates and growth media (Fig. 1), as well as between monocultures and co-cultures. Most clinical isolates of *P. aeruginosa* (Tmp04, Tmp05, Tmp07) and *E. coli* (Tme12, Tme13) monocultures, along with some co-cultures (Pair412, Pair715), formed similar or more biofilm when grown in AUM medium compared to reference strains (PA01, K12, PairEP). However, Tmp06 and Tme14 showed lower biofilm formation in both monoculture and co-culture compared to the reference strains in AUM (Fig. 1).

**Fig. 2:**
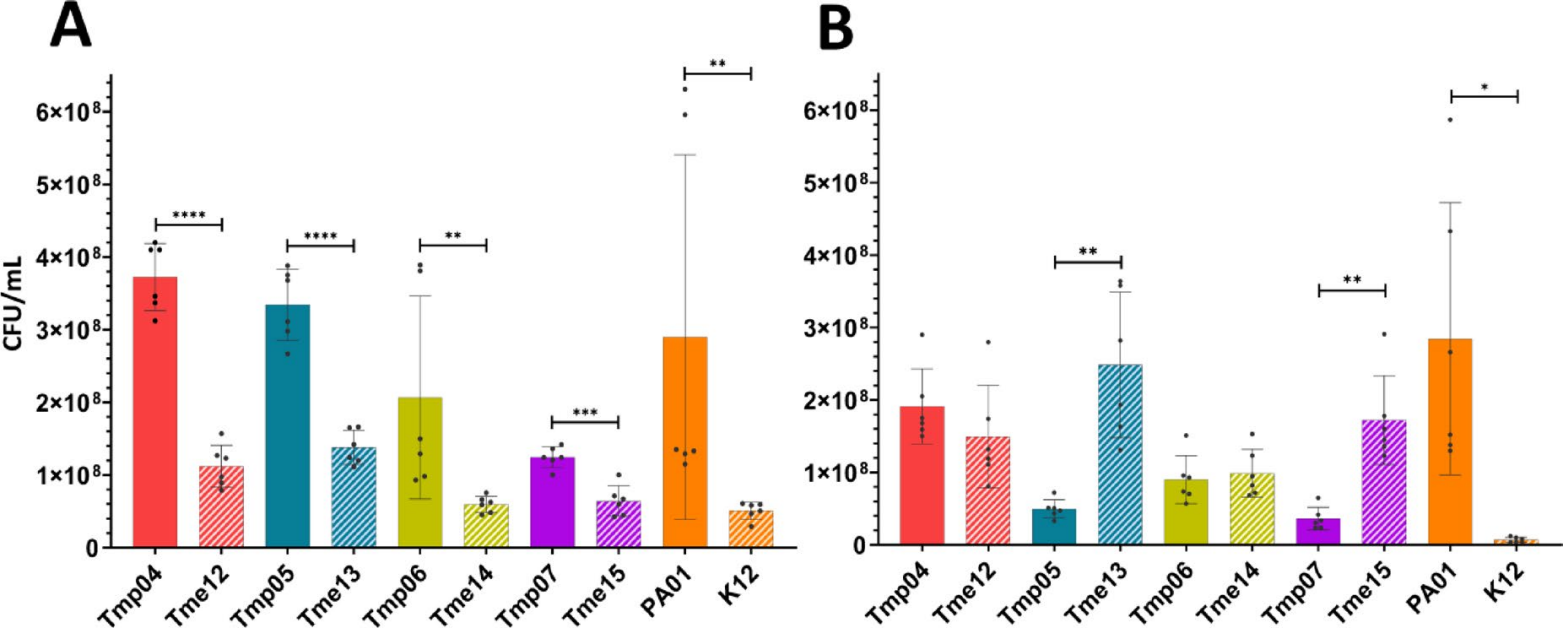
Viable counts of clinical strains in pairs from biofilms formed in AUM and ISO in 96-well plates (strains from pairs are represented with the same colour but with checked bars for *E. coli*). (**A**) Bacterial ratio of CAUTI strains in AUM; (**B**) Bacterial ratio of CAUTI strains in ISO. Data are expressed as mean ± standard deviation from 6 replicates per sample. Statistical significance between groups was determined using an unpaired t-test with Welch’s correction (for normally distributed data) or Mann–Whitney U test (for non-normal data), as appropriate. Significance levels are indicated as: * for p ≤ 0.05, ** for p ≤ 0.01, *** for p ≤ 0.001, and **** for p ≤ 0.0001.

In the biofilm assay for ISO, only Tmp06 and Tmp04 monocultures showed biofilm formation comparable to the reference PA01 monoculture. Co-cultures generally showed reduced biofilm formation in ISO compared to their corresponding *P. aeruginosa* monocultures. However, the clinical co-culture Pair614 and the reference co-culture PairEP formed more biofilm than their respective *P. aeruginosa* monocultures. When comparing biofilm formation across the three media, monocultures and co-cultures from clinical strains generally gave more biofilm in AUM than in ISO or LB (Fig. 1). Tmp monocultures and co-cultures also formed more biofilm in ISO, while most isolates showed poor biofilm formation in LB. These findings suggest that clinical isolates are more effective at forming biofilm under low-nutrient conditions, as in AUM, than in nutrient-rich conditions. No obvious correlation of biofilm volume with cell and culture phenotypes (e.g. hydrophobicity or motility) was seen. The crystal violet biofilm assay, which was used here to evaluate biofilm formation, stains both bacteria and extracellular polymeric substances (EPS) within the biofilm matrix. Thus, these results may be influenced by factors other than the actual quantity of bacterial cells in biofilms. To address this, viable counts of dual-species biofilms were done to determine the ratio of *P. aeruginosa* and *E. coli* cells in the mixed biofilms, along with their respective contributions to the dual-species biofilms. The data from viable counts showed that *Pseudomonas* colony-forming units (CFUs) were higher than *E. coli* CFUs in mixed cultures formed in AUM (Fig. 2). However, in ISO, Tmes either had similar or higher CFUs than Tmps. The dual culture of reference strains showed more PA01 CFUs than K12 in both conditions. The combined biofilm and viable counts data, thus, indicated that Tmps grew relatively better in AUM. Biofilm formation was more pronounced than for the co-culture or Tme mono-culture, and in the co-culture data Tmps reached higher viable counts. We hypothesize that this difference was related to nutrient composition.

### Metabolite profiles of clinical CAUTI strains

**Fig. 3:**
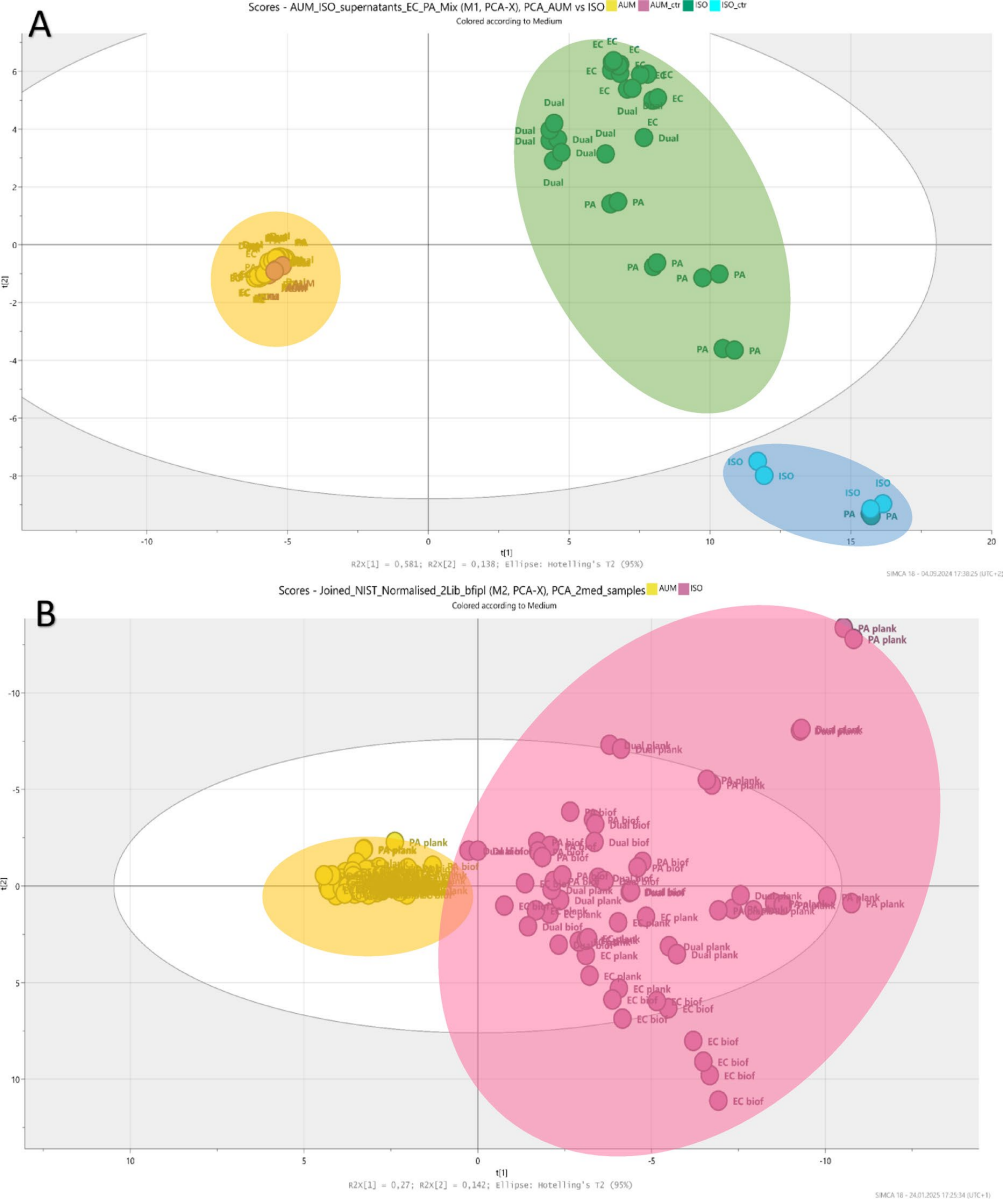
PCA models of mono- and co-cultures grown in AUM and ISO. (**A**) PCA model of spent media samples (R2X = 0.91, Q2 = 0.81) showing three distinct clusters: yellow circle (bacterial samples and medium control in AUM), green circle (bacterial samples in ISO excluding Tmp05), and blue circle (ISO medium control and Tmp05). Sample colour coding (small circles): yellow = bacterial samples in AUM, pink = AUM medium control, cyan = ISO medium control, green = bacterial samples in ISO. (**B**) PCA model of planktonic and biofilm cell samples (R2X = 0.68, Q2 = 0.45) showing separation into two major groups: yellow circle (samples from AUM) and pink circle (samples from ISO). Bacterial samples from AUM and ISO had 3 and 2 technical replicates, respectively; medium controls had 6 replicates (AUM) and 4 replicates (ISO).

**Fig. 4:**
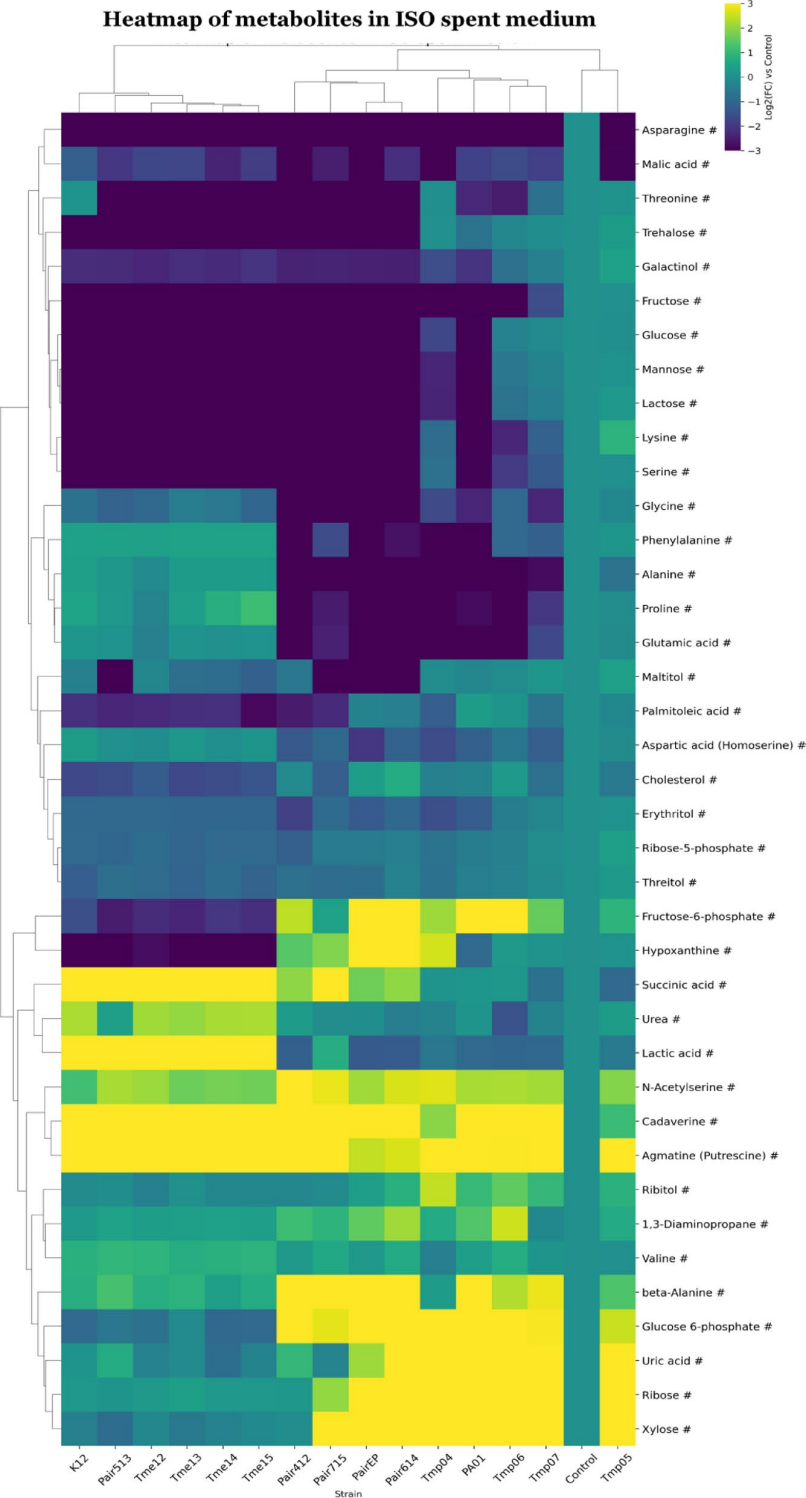
Clustered heatmap of significant metabolites in ISO spent medium. Metabolites are marked based on their significance: those that are statistically significant (p-value < 0.05 after FDR correction) are marked with an asterisk (*), while those with a significant fold-change (absolute log2FC > 0.75) are marked with a hashtag (#). Metabolites that meet both criteria are left unmarked. The FC for each metabolite in bacterial samples was calculated relative to its peak area in the medium control sample. Heatmap was generated using Cowtea v1.0.0 (repository link https://github.com/sokoljator/CAUTI-metabolomics)

**Fig. 5:**
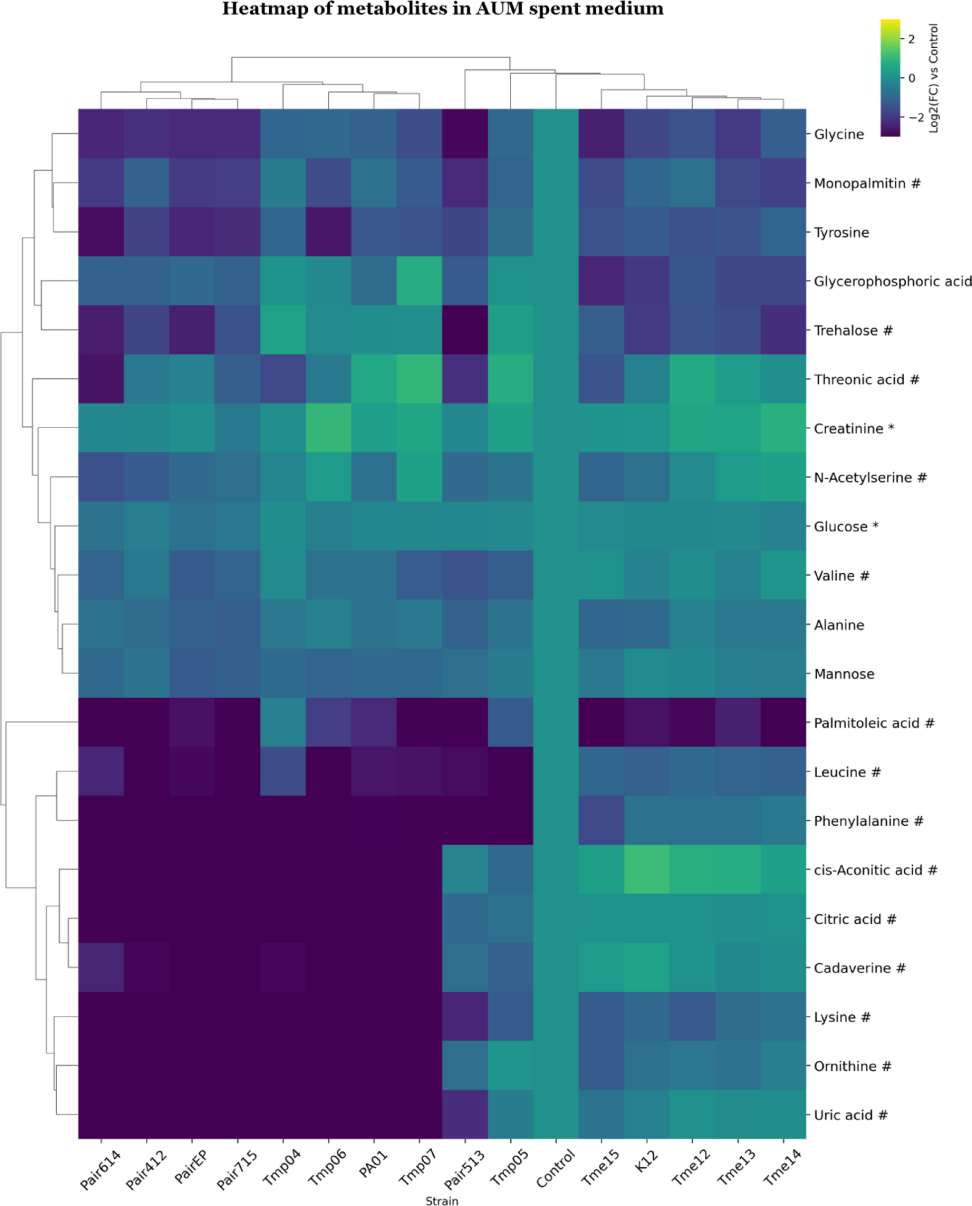
Clustered heatmap of significant metabolites in AUM spent medium. Metabolites are marked based on their significance: those that are statistically significant (p value < 0.05 after FDR correction) are marked with an asterisk (*), while those with a significant fold-change (absolute log2FC > 0.75) are marked with a hashtag (#). Metabolites that meet both criteria are left unmarked. The FC for each metabolite in bacterial samples was calculated relative to its peak area in the medium control sample. Heatmap was generated using Cowtea v1.0.0 (repository link https://github.com/sokoljator/CAUTI-metabolomics)

**Fig. 6:**
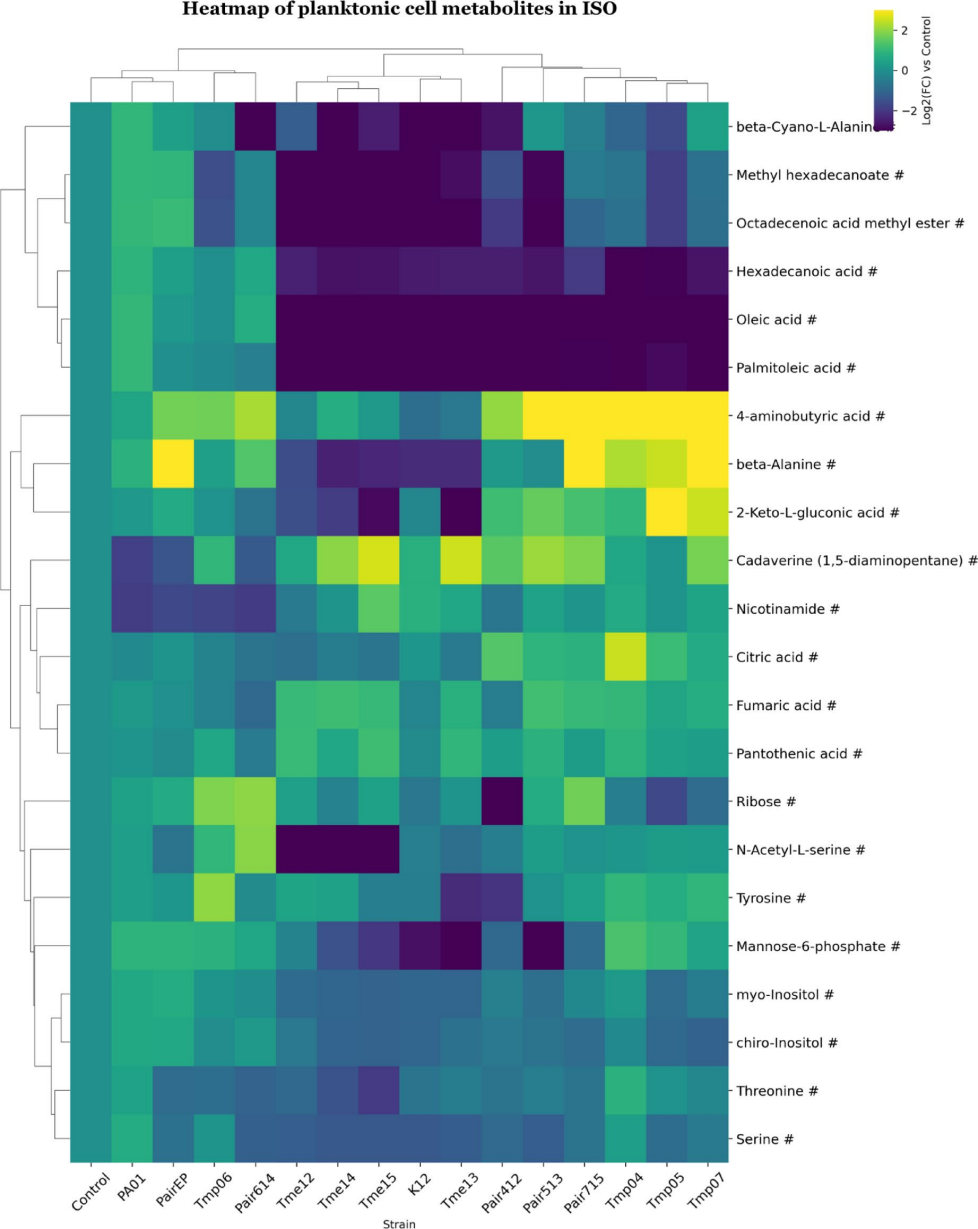
Clustered heatmap of significant metabolites in mono- and co-cultures of planktonic cell samples in ISO. Metabolites are marked based on their significance: those that are statistically significant (p-value < 0.05 after FDR correction) are marked with an asterisk (*), while those with a significant fold-change (absolute log2FC > 0.75) are marked with a hashtag (#). Metabolites that meet both criteria are left unmarked. The FC for each metabolite in bacterial samples was calculated relative to its peak area in the control (average metabolite composition of K12 and PA01). Heatmap was generated using Cowtea v1.0.0 (repository link https://github.com/sokoljator/CAUTI-metabolomics)

**Fig. 7:**
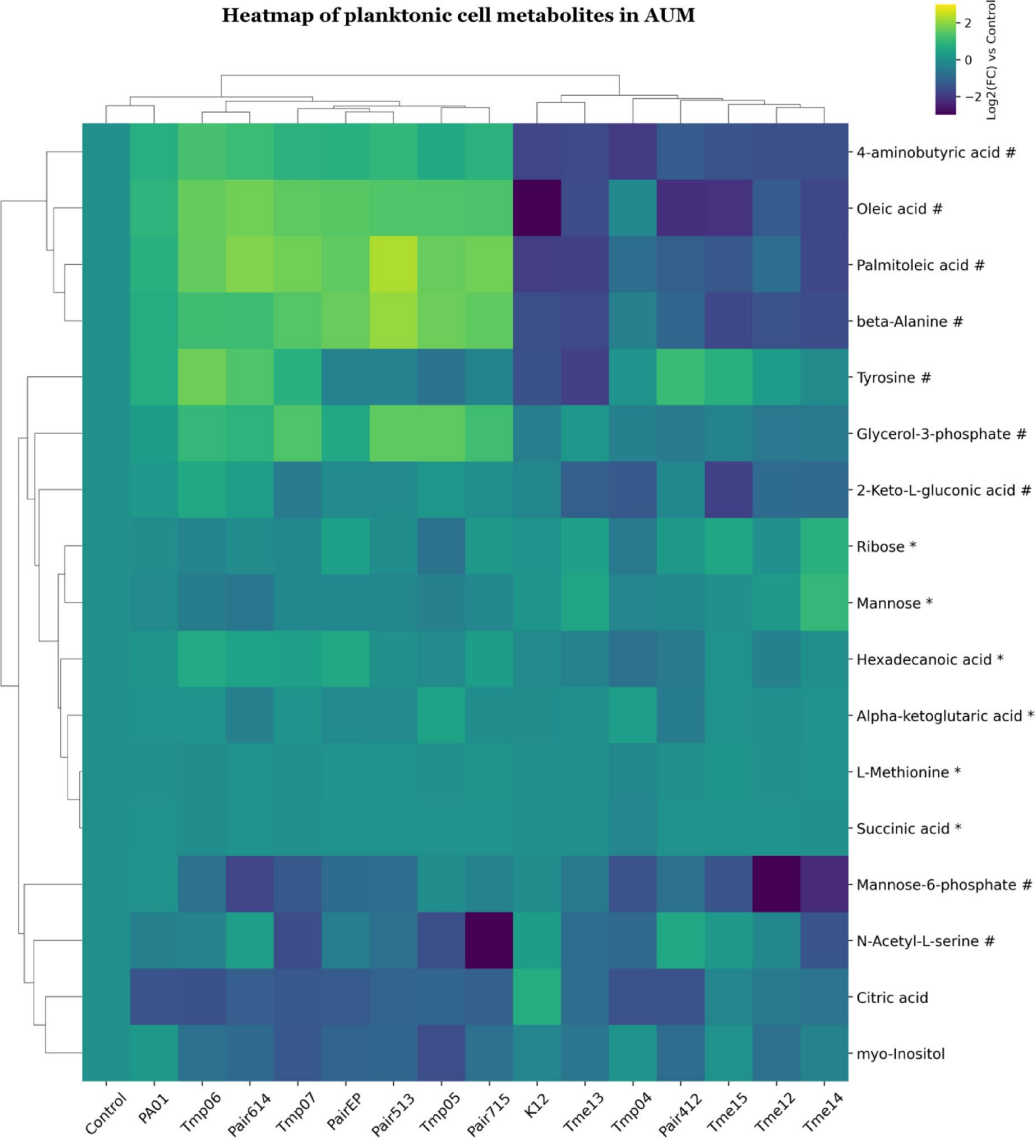
Clustered heatmap of significant metabolites in mono- and co-cultures of planktonic cell samples in AUM. Metabolites are marked based on their significance: those that are statistically significant (p-value < 0.05 after FDR correction) are marked with an asterisk (*), while those with a significant fold-change (absolute log2FC > 0.75) are marked with a hashtag (#). Metabolites that meet both criteria are left unmarked. The FC for each metabolite in bacterial samples was calculated relative to its peak area in the control (average metabolite composition of K12 and PA01). Heatmap was generated using Cowtea v1.0.0 (repository link https://github.com/sokoljator/CAUTI-metabolomics)

**Fig. 8:**
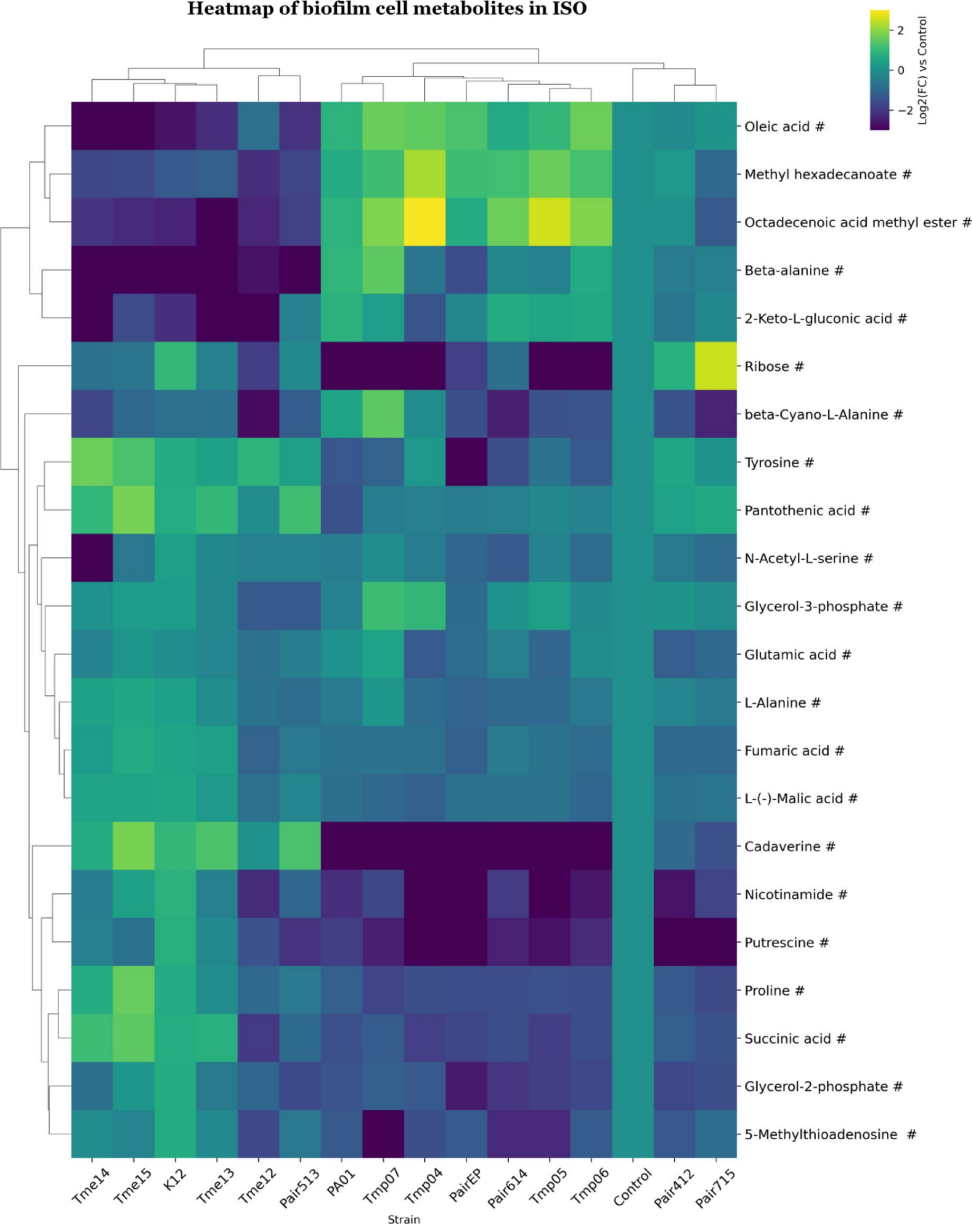
Clustered heatmap of significant metabolites in mono- and co-cultures of biofilm cell samples in ISO. Metabolites are marked based on their significance: those that are statistically significant (p-value < 0.05 after FDR correction) are marked with an asterisk (*), while those with a significant fold-change (absolute log2FC > 0.75) are marked with a hashtag (#). Metabolites that meet both criteria are left unmarked. The FC for each metabolite in bacterial samples was calculated relative to its peak area in the control (average metabolite composition of K12 and PA01). Heatmap was generated using Cowtea v1.0.0 (repository link https://github.com/sokoljator/CAUTI-metabolomics)

**Fig. 9:**
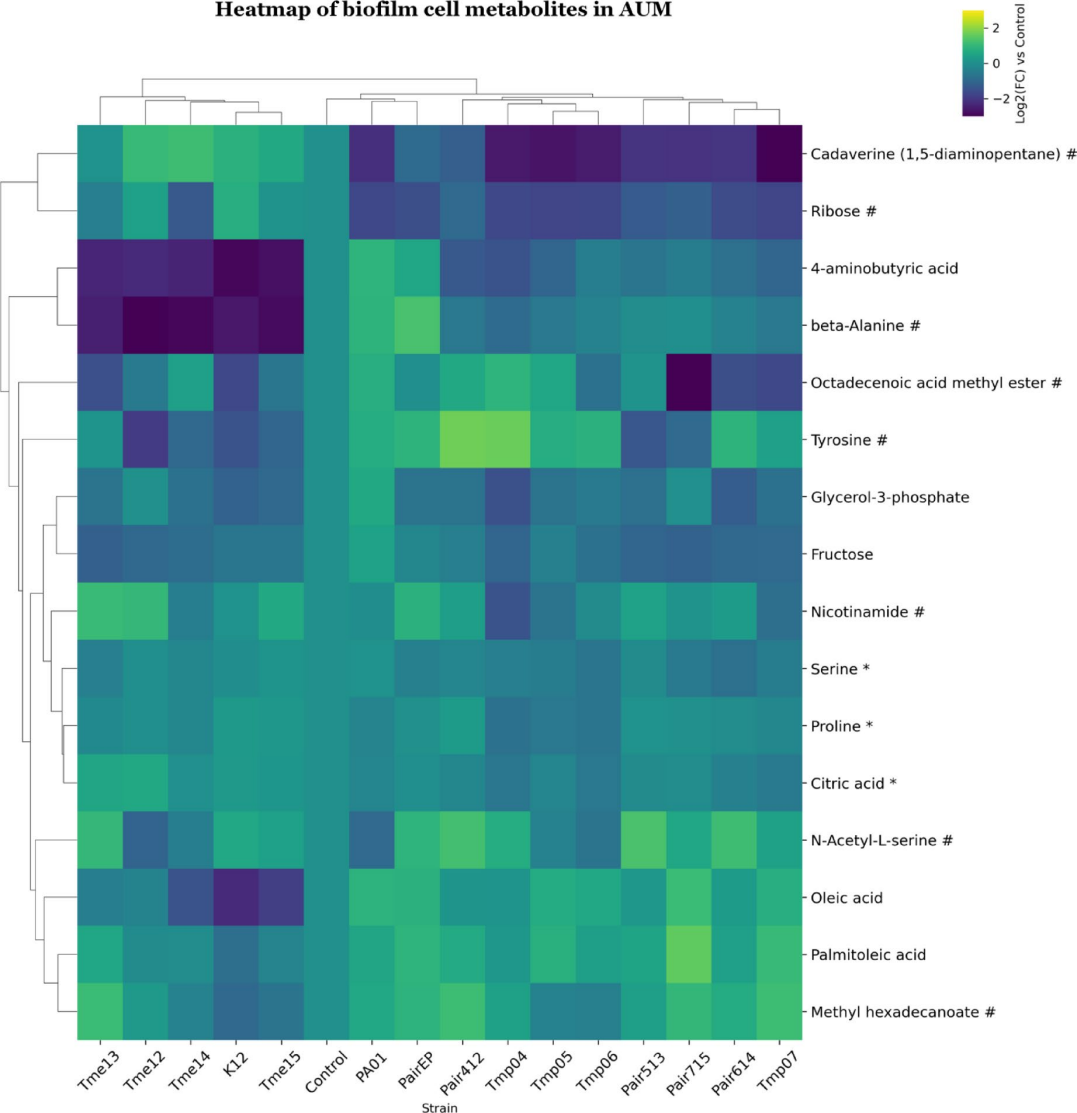
Clustered heatmap of significant metabolites in mono- and co-cultures of biofilm cell samples in AUM. Metabolites are marked based on their significance: those that are statistically significant (p-value < 0.05 after FDR correction) are marked with an asterisk (*), while those with a significant fold-change (absolute log2FC > 0.75) are marked with a hashtag (#). Metabolites that meet both criteria are left unmarked. The FC for each metabolite in bacterial samples was calculated relative to its peak area in the control (average metabolite composition of K12 and PA01). Heatmap was generated using Cowtea v1.0.0 (repository link https://github.com/sokoljator/CAUTI-metabolomics)

Metabolomics was used to investigate metabolite differences in planktonic cells, biofilms, as well as in the culture medium. The culture medium was studied in order to investigate how bacteria utilised nutrients and which metabolites were produced. The medium after culture growth will hereafter be called “spent medium”. Metabolite data were collected from mono- and dual-species cultures (spent medium, planktonic cells and biofilm cells) grown in AUM and ISO. Principal component analysis (PCA) and orthogonal partial least squares discriminant analysis (OPLS-DA) were used to analyse patterns in metabolome profiles. The comparison of intracellular metabolites between planktonic and biofilm samples was performed to study the metabolic differences between these two bacterial lifestyles. In the PCA model for spent medium, AUM samples were tightly clustered, while ISO samples showed higher metabolic diversity spreading along the y-axis (Fig. 3A). A similar pattern was observed in the PCA model for planktonic and biofilm cells, where AUM samples formed compact clusters compared to more dispersed ISO samples (Fig. 3B). These results showed that distinct differences in metabolic profiles were observed between the two medium conditions. To further investigate these differences, separate models were built for each media as well as sample type, as described in the supplemental material. From these models, significant metabolites were identified and are presented in heatmaps (Figs. 4, 5, 6, 7, 8 and 9).

### Metabolites in spent medium

In the dataset from the ISO spent medium, samples were clustered into two main groups in the heat map where the second was split into three subgroups (Fig. 4). The groups were: Group 1) All Tmes with K12 and Pair513; Group 2a) clinical co-cultures with control co-culture; Group 2b) PA01 with all Tmps except Tmp05, which was clustered together with the medium control as Subgroup 2c. Thus, clustering appeared to be related to genus and mono-/co- culture (Fig. 4). That Tmp05 clustered together with the medium control suggested weak growth in monoculture. Such weak growth for Tmp05 probably also shifted Pair513 to the Tme group. The first group (Tmes & K12 & Pair513) showed a pronounced increase of succinic acid, urea, lactic acid, N-acetylserine, cadaverine and agmatine/putrescine in the spent medium compared to the control ISO medium, indicating possible production and/or release of these metabolites into the medium. Group 2 showed increases in N-acetylserine, cadaverine and agmatine/putrescine compared to the pure medium (Fig. 4). Group 2 also showed an increase for fructose-6-phosphate, beta-alanine, glucose-6-phosphate, uric acid, ribose, and xylose, which was not seen for Group 1. In addition, Group 2a showed an increase in hypoxanthine. Some metabolites were also decreased compared to the control. There was a pronounced decrease in glycine, phenylalanine, alanine, proline and glutamic acid for Group 2, while threonine, trehalose, fructose, glucose, mannose, lactose, lysine, and serine were decreased most in Group 1 and Group 2a. Interestingly, asparagine was decreased in all groups, suggesting it was consumed by all strains.

In the AUM spent medium dataset, fewer metabolites were found to be significantly altered (Fig. 5). The samples from the AUM dataset were clustered similarly to the clustering in ISO, except that Pair 513 shifted to Group 2c next to Tmp05, again indicating low growth. Thus, the same group numbering was used for both spent media. The metabolites: leucine, phenylalanine, cis-aconitic acid, citric acid, cadaverine, lysine, ornithine, and uric acid decreased to a large extent in Group 2. However, leucine, phenylalanine, lysine, and ornithine were decreased to a lower extent in Group 1, and cis-aconitic acid was increased compared to the medium control (Fig. 5). Glycerophosphoric acid and trehalose were decreased in Group 1 and Group 2a. Palmitoleic acid was consumed from the medium in most samples.

### Planktonic cell metabolites

In the ISO medium, planktonic samples clustered into 3 main groups, slightly different from the spent medium (Fig. 6). One group formed by Tmp04, Tmp05 and Tmp07 and their co-cultures (Group 3). A second group similar to Group 1 in the spent medium (Tmes & K12) and a third group where the control clustered together with PA01, PairEP, Tmp06 and Pair614 (Group 4). The close clustering of monocultures of *Pseudomonas* next to their co-cultures indicated a close similarity and large effect of *Pseudomonas* on the metabolite pattern of the co-culture (Fig. 6). Fatty acids (palmitoleic acid, oleic acid, hexadecenoic acid, octadecanoic acid methyl ester, and methyl hexadecanoate) were decreased in Groups 1 and 3. Group 3 showed a pronounced increase in 4-aminobutyric acid, beta-alanine, and 2-keto-L-gluconic acid, while in Group 1 these metabolites were mostly decreased. Interestingly, cadaverine and nicotinamide were decreased in Group 4 but not in the other groups.

Planktonic cells from the AUM medium clustered into two major groups (Fig. 7). One resembling Group 1 with *E. coli* monocultures, but with the addition of Pair412. The other group consisted of *Pseudomonas* monocultures and the remaining co-cultures. The most notable difference between groups was observed in 4-aminobutyric, oleic acid, palmitoleic acid and beta-alanine, which were decreased in *E. coli* monocultures but increased in other samples.

### Biofilm cell metabolites

In the ISO biofilm dataset, samples were clustered into two groups (Fig. 8). One *E. coli* group similar to other conditions but with one additional co-culture (Tmes & K12 & Pair513). A second larger group with *Pseudomonas* mono and co-cultures where two co-cultures (Pair412 & Pair715) and the control were clustered together into a subgroup. For Group 1 (and Pair513) the content of oleic acid, methyl hexadecanoate, octadecanoic acid methyl ester, beta-alanine, and 2-keto-L-gluconic acid was decreased. Cadaverine, nicotinamide and putrescine were decreased in all Tmps (mono- and co-cultures), whereas *E. coli*, monocultures and Pair513 showed increased cadaverine levels (Fig. 8). Also, for AUM biofilms, samples clustered in two groups (Fig. 9). One group with monocultures of *E. coli* (Group 1, all Tmes & K12), and a second larger group with the rest of the biofilm samples, where *Pseudomonas* reference strain monoculture and coculture formed one subgroup with the control. Beta-alanine and 4-aminobutyric acid were decreased in *E. coli* monocultures. Cadaverine and ribose were decreased in *Pseudomonas* monocultures and co-cultures.

## Discussion

Catheter-associated urinary tract infections (CAUTIs) represent common medical device-related infections that become increasingly complicated with prolonged catheterisation in patients. Over time, multi-species communities develop stable biofilms that are difficult to eradicate, leading to chronic infections in patients^25,26,29^, as well as catheter blockages caused by crystalline biofilms^27,28,44,45^. These incrustations result from urine alkalinisation due to urease-producing bacteria, which precipitate salts from the urine^11,27,28^. In our collection of clinical bacterial strains, urease activity was not observed in Tmes, but it was observed to a low degree in Tmps (Supplementary information Figure S20). This indicates that neither Tmes nor Tmps had the potential to cause encrustation in urinary catheters^27^. No clear correlation was observed between the physicochemical properties of the isolates and their biofilm formation in 96-well plates (Fig. 1, Supplementary information Figure S1). The CAUTI isolates generally formed more biofilm in AUM than in media with higher nutrient content, both as individual strains and in co-culture (Fig. 1). In multispecies biofilms, the ratio of different bacteria may vary^18,46^, which was also observed in our study. Viable counts showed that clinical *P. aeruginosa* had higher cell counts in biofilms formed in AUM. However, higher cell counts of clinical *E. coli* were observed in dual-species biofilms grown in ISO (Fig. 2). This observation aligns with previous studies^47,48^, which suggests that nutrient availability influences interspecies interactions and biofilm composition. The results also indicated that bacteria may rely on different metabolic pathways in the media, leading to variations in bacterial interactions depending on nutrient availability.

### Metabolite analysis of bacterial samples in ISO and AUM media

**Fig. 10:**
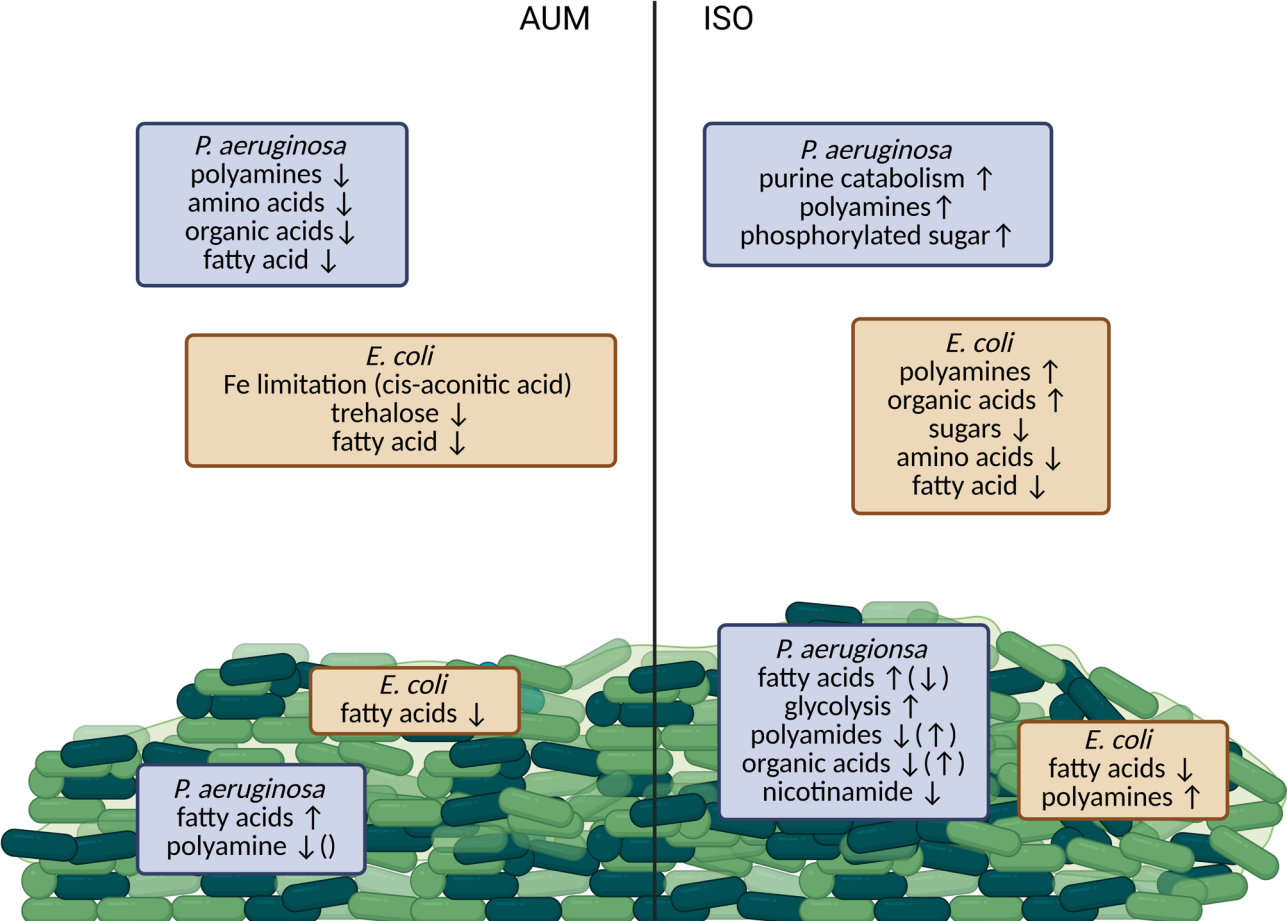
Conceptual drawing summarizing selected changes in metabolite pattern for supernatants and in bacterial cell pellets during growth in artificial urine medium (AUM) or in iso-sensitest medium (ISO) for *P. aeruginosa* and *E.coli* CAUTI isolates. Metabolites specified in boxes at the bottom of the drawing and inside the biofilm cartoon represent changes in intracellular metabolites. Metabolites in boxes in the surrounding fluid represent changes observed in the metabolite pattern in filtered cell supernatants. Intracellular metabolite changes represent changes observed both for planktonic cells and biofilms. However, when arrows are given in parenthesis, this denotes a difference between biofilm cells and planktonic cells, the latter being in parenthesis. Co-cultures, in general exhibited metabolite changes similar to *P aeruginosa* cultures. (Created in BioRender. Ramstedt, M. (2026) https://BioRender.com/08446la).

The analysis of the metabolite patterns for *E. coli* and *P. aeruginosa* monocultures and respective co-cultures showed noticeable differences in the metabolite levels and metabolite composition depending on the growth media (ISO and AUM) and sample types (spent medium, planktonic cells, and biofilm cells). In the medium, metabolite patterns indicated which metabolites were consumed and utilised by bacteria, as well as which substances were released into the surrounding environment. On the other hand, metabolite patterns in the cell samples reflect intracellular metabolites, highlight differences in the accumulation or consumption of cellular metabolites under different conditions (Fig. 10).

### Metabolite analysis of ISO supernatants

After cultivation of *E. coli* monocultures, the ISO spent medium (Fig. 4) showed an increase in metabolites associated with amino acid catabolism (e.g., urea, agmatine/putrescine and cadaverine)^49,50^; anaerobic glycolysis^51^ (e.g., lactic acid), and tricarboxylic acid (TCA) cycle^52^ (e.g., succinic acid). In addition, sugars were decreased as well as amino acids such as threonine, lysine, and serine. This suggested that monocultures of *E. coli* were actively involved in amino acid catabolism, protein synthesis^53^^,^^54^ and using glycolytic pathways^55^. Previous studies have described amino acid catabolism as an adaptation to urine for pathogenic *E. coli* strains^56^. Additionally, supernatants of *E. coli* monocultures had decreased levels of metabolites linked to nucleotide metabolism^53^ (hypoxanthine), fatty acid biosynthesis or changes in membrane composition^54^^,^^57^ (palmitoleic acid). Metabolite patterns in spent medium from *P. aeruginosa* monocultures and co-cultures showed increased levels of metabolites associated with purine catabolism^53,54,58^ (e.g., uric acid, ribose) as well as pyrimidine catabolism and synthesis of Coenzyme A^53,58,59^ (beta-alanine). In addition, fructose-6-phosphate and glucose-6-phosphate were increased, indicating that the Embden-Meyerhof glycolytic pathway (EMP) and the synthesis of polysaccharides and peptidoglycan were either inactive or rate-limiting. This likely led to the accumulation and subsequent release of these phosphorylated intermediates into the medium^55^, even though glucose was taken up from the medium and phosphorylated. Alternatively, this metabolite was exchanged and cross-fed between bacterial cells, as has been described for intermediates in amino acid synthesis^60^. The high abundance of uric acid and ribose in spent medium in *Pseudomonas* suggested an active purine degradation pathway, and increased xylose may be linked to an active pentose phosphate pathway. These pathways have been found to play important roles in managing oxidative stress^61,62^, which may be caused by the release of superoxides during purine degradation^53^. The metabolite pattern in the spent media for cocultures was similar to monocultures of both species, giving a broad consumption of amino acids (Fig. 4). In addition, some specific increases were observed in co-culture for metabolites associated with nucleotide turnover and salvage pathways^53,63^ (e.g., hypoxanthine, beta-alanine), as well as sulphur amino acid metabolism (e.g., N-acetylserine)^54,64^.

### Metabolite analysis of AUM supernatants

Differences in metabolite profiles in the AUM spent medium were mainly related to differences between *E. coli* monocultures and the rest of the samples (Fig. 5). *E. coli* monocultures had increased levels of cis-aconitic acid, which is a TCA cycle intermediate^52^. This suggests accumulation and release of cis-aconitic acid into the medium, potentially due to the low iron levels in AUM^30^. Low iron could inhibit the activity of aconitase, the enzyme that catalyses the conversion of citrate into isocitrate. In contrast, *Pseudomonas* and co-culture groups had relatively low levels of cis-aconitic acid along with citric acid, cadaverine (a diamine formed from lysine) and ornithine (a precursor of amino acids such as citrulline and arginine^54^, as well as other amino acids). *P. aeruginosa* uses amino acid porins in the outer membrane to transport arginine, lysine, histidine, ornithine and various peptides into the cell^65^. This suggests that *P. aeruginosa* strains used organic acids as carbon sources, ornithine for polyamine or amino acid synthesis, and amino acids for protein synthesis. Polyamines have been described as critical for bacterial responses to nitrosative stress in AUM medium and may contribute to resistance against pH increases^49,66^. This is in line with the alkalinisation of media observed for these *P. aeruginosa* isolates. However, polyamines have a wide variety of functions in bacterial cells^39^. Interestingly, trehalose was primarily consumed by the *E. coli* cultures (both mono and cocultures) in both media. In AUM, this may reflect a response to the relatively low nutrient content. Trehalose may enhance survival in nutrient-limited conditions as it can be converted to the storage polymer glycogen to sequester carbon if a carbon sources are available^67–70^. However, trehalose was also consumed in more nutrient-rich conditions, suggesting that it may also simply serve as a suitable carbon source^71^. The similarity between the *Pseudomonas* monoculture and co-cultures aligns with viable count data from co-cultures in AUM, which showed a higher abundance of *P. aeruginosa* cells than *E. coli* cells (Fig. 2).

Some differences emerged when comparing monocultures and co-cultures in ISO and AUM media. In AUM, *E. coli* appeared to experience possible iron limitation, which may explain the lower bacterial counts observed (Fig. 2). In the ISO medium, all cultures showed indications of active anabolic and catabolic metabolisms. In addition, some organic acids (succinic acid lactic acid) released by Tmes in monocultures were absent in co-cultures, indicating either altered metabolism in *E. coli* during coculture or consumption of these metabolites by *P. aeruginosa*. This observation is in line with previous studies describing that *E. coli* may utilise a mixture of respiratory and fermentative metabolism in ample supply of carbon sources^72^. This strategy gives rise to a number of organic acids lowering pH and giving *E. coli* a growth advantage compared to many other bacterial species, e.g. *P. aeruginosa*^73^. However, the effect has been described as strain specific and the results here, suggest that *P. aeruginosa* strains in the urinary tract may have been under selective pressure to use these organic acids as a carbon source, thereby obtaining a constant supply of carbon while reducing the ability of *E. coli* to lower pH. Yasir et al. described that this metabolic strategy in *E. coli*, involving organic acids, relies on the presence of glucose^72^. In the two media used here, only ISO contained glucose, suggesting that this metabolic strategy would not take place in the same way in AUM. Together with the hypothesised iron limitation, this may explain why *E. coli* cell numbers were lower than those for *Pseudomonas* in co-cultures in AUM.

### Metabolite analysis of planktonic and biofilm cultures

After investigating metabolites from the spent medium, we analysed the intracellular metabolites in monocultures and cocultures. To facilitate comparisons, the metabolite patterns for planktonic and biofilm cell datasets were referenced against an average metabolite content for K12 and PA01 (control, Figs. 6, 7, 8 and 9). The clustering of cell samples in the ISO medium, indicated that the metabolite profile for *Pseudomonas* dominated for all co-culture pairs. *Pseudomonas* planktonic monocultures and co-cultures demonstrated a decrease of metabolite levels associated with fatty acid biosynthesis^54^ or routinely detected as fragments from phospholipids^74^ (e.g., palmitoleic acid (planktonic cells). However, oleic acid, and octadecenoic methyl ester (both planktonic and biofilms) were increased in biofilms but decreased in planktonic cells. Metabolites linked to glycolysis via the Entner–Doudoroff (ED) pathway^55^ (e.g., 2-ketogluconic acid), and CoA synthesis^59^ (e.g., beta-alanine) increased in both biofilm and planktonic cells. The difference between the accumulation of the fatty acid oleic acid and octadecenoic methyl ester between cells in planktonic form and biofilms may relate to differences in cell membrane. Ratios between different types of fatty acids have been described as key regulators of membrane fluidity^75,76^. This could suggest that *P. aeruginosa* adapts to biofilm growth by changing fatty acid production and/or membrane composition. It has been described that bacterial cells in the more nutrient-rich outer parts of a biofilm can provide fatty acids to cells in the interior of the biofilm in exchange for amino acids^77^. Such metabolic interactions may level out differences between cells in co-culture biofilms and possibly explain why biofilm cocultures did not show the same level of depletion of intracellular fatty acids as planktonic *E. coli* bacteria did (Figs. 6, 7, 8 and 9). The levels of malic acid, cadaverine, nicotinamide, agmatine/putrescine and succinic acid were decreased in *P. aeruginosa* mono and coculture biofilms compared to control. However, planktonic cultures had similar or higher levels compared to the control. Additionally, the accumulation of 2-ketogluconic acid in the *P. aeruginosa* group and increased cadaverine in planktonic cells from amino acid catabolism (with putrescine and cadaverine as end products^78^ might indicate that planktonic cells may have experienced greater oxidative stress than biofilm cells^55,62^ or that the two lifestyles had differences in uptake and utilisation of these amines as nitrogen sources^79^. These findings are consistent with solution data showing that *P. aeruginosa* released polyamines like agmatine/putrescine into solution. These may have been taken up by *E. coli* and used as carbon and nitrogen sources by conversion into 4-aminobutyric acid and succinic acid that can enter the TCA cycle^78^. In addition, *Pseudomonas* appeared to consume organic acids such as succinic acid and lactic acid formed by *E. coli* when grown in coculture (Fig. 4). Nicotinamide is part of the metabolism of the coenzyme nicotinamide adenine dinucleotide (NAD+). Thus, differences between samples may indicate differences in electron carriers, or their metabolism, between the cultures^80^. All *Pseudomonas* mono and cocultures showed lower nicotinamide levels than the control in biofilm samples, but not in planktonic cell samples. This may suggest higher NAD+ turnover or more efficient NAD+ recycling in biofilms, as well as in Tmps and co-cultures^81,82^.

In the AUM medium, the metabolite patterns were even more similar between biofilms and planktonic cells than in ISO, but differences were observed between *E. coli* monocultures and *P. aeruginosa* monocultures and co-cultures. *E. coli* monocultures had lower levels of fatty acids (e.g., oleic and palmitoleic acids) than the other cultures, and this was especially pronounced for planktonic cells. These differences suggest that *P. aeruginosa* cells had a more active fatty acid biosynthesis with respect to these compounds or were remodelling their membrane. Furthermore, *Pseudomonas* produced stress response metabolites in planktonic form (such as 4-aminobutyric acid), possibly following alkalinisation of the medium or other stressors. The metabolite 4-aminobutyric acid has been linked to enhanced stress response mechanisms, interkingdom signalling^82,83^ and may also serve as a carbon and nitrogen source for bacteria^78^. These metabolic shifts indicate that *Pseudomonas* growth advantage in co-cultures in AUM may be due to an ability to grow using alternative pathways, for example using polyamine degradation (converting agmatine/putrescine into 4-aminobutyric acid and succinic acid).

In conclusion, clinical isolates generally formed more biofilms in AUM than in the nutrient-rich medium, both as single-species and dual-species cultures. In dual-species biofilms, *P. aeruginosa* cells consistently outnumbered *E. coli* cells, consistent with previous research^11^. This advantage appeared to be linked to iron availability and alkalinisation of the medium. However, in nutrient-rich conditions, viable cell counts were more comparable between the two species for clinical strains, though less so for reference strains. Interestingly, in some clinical pairs, *E. coli* demonstrated a higher cell count within biofilms than *P. aeruginosa*, suggesting strain-specific interactions and metabolic strategies. This advantage is most likely linked to the ability of *E. coli* to use a metabolic strategy mixing respiratory and fermentative metabolism in the presence of glucose^72^, lowering culture pH and producing organic acids. The differences observed between media indicate that nutrient-rich media may not accurately reflect the mechanisms occurring in the urinary tract, emphasising the suitability of model media more closely resembling clinical conditions, such as the AUM medium. The reduced *E. coli* viable cell counts in AUM could be connected to several factors. *P. aeruginosa* isolates exhibited urease activity, which likely increased pH and negatively impacted the growth of *E. coli* strains and their fitness in the biofilms. Furthermore, metabolite patterns indicated that *E. coli* cells experienced iron limitation in AUM (accumulation of cis-aconitic acid), further hindering their growth, while no such effect was observed in *P. aeruginosa* strains. *P. aeruginosa* showed indications of adaptations including production of stress-response metabolites (4-aminobutyric acid) and usage of polyamines. Production and secretion of polyamines are in line with previous studies of both clinical samples (urine) as well as studies of bacterial metabolites in uropathogenic bacteria^36,38,39^. In co-cultures, the intracellular metabolome patterns were highly influenced by *P. aeruginosa* strains in both ISO and AUM media and for both planktonic and biofilm cultures. Furthermore, the data in ISO medium suggested potential metabolic interactions between the species, where *E. coli* appeared to produce organic acids consumed by *Pseudomonas* strains in co-culture. An interesting topic for future studies would be to investigate the long-term effects of these metabolic interactions at the two nutrient levels. Possibly the growth disadvantages experienced by *E. coli* eventually lead to a mono-species situation where only *P. aeruginosa* remains, in conditions resembling those of AUM.

The results from metabolite datasets revealed differences between the species, indicating that both species adjusted their metabolism based on surrounding nutrients and possibly the presence of other bacterial species. This aligns with previous publications discussing the benefit of interspecies cross-feeding and resource sharing in bacterial multispecies communities^17^. The data in this study suggest that such interactions may occur between clinical isolates from patients with CAUTI. The data further suggests that biofilms formed in urinary catheters are predominantly composed of *P. aeruginosa*, but that this species may benefit from the presence of *E. coli* due to complementary metabolisms between the two species.

## Materials & methods

### Bacterial strains

Clinical strains in this study originated from Norrland’s University Hospital (NUS, Umeå, Sweden). Urine samples from patients with suspected urinary tract infection (UTI) were sent to the Department of Clinical Microbiology, Umeå University and NUS, for culture, species identification and antimicrobial susceptibility tests, information used for handling of clinical patients. The denoted isolates were anonymized for the researchers and contained no clinical or personal information and no human cells or DNA/RNA. The procedure follows the ethical guidelines, approval, and regulations at Department of Clinical Microbiology, NUS, and nationally. The need for informed consent was waived by Norrland’s University Hospital because no information or donated material can be traced to an individual. The isolates were typed using matrix-assisted laser desorption ionisation-time of flight mass spectrometry (MALDI-TOF MS; Bruker Daltronics, Bremen, Germany) at the Department of Clinical Microbiology (Umeå University and NUS, Umeå, Sweden, accreditation no 137). A MALDI-TOF score higher than 2.0 was deemed acceptable for species identification (individual scores were all higher than 2.3 for each strain). Catheter isolates that originated from patients with both *Pseudomonas aeruginosa* and *Escherichia coli* were chosen to form four clinical pairs. These included *P. aeruginosa* (Tmp04 = CCUG 78363, Tmp05 = CCUG 78364, Tmp06 = CCUG 78365, Tmp07 = CCUG 78366) and *E. coli* (Tme12 = CCUG 78367, Tme13 = CCUG 78368, Tme14 = CCUG 78369, Tme15 = CCUG 78370). (Please note, that in plural the abbreviation Tmes and Tmps will be used to refer to the two groups of clinical strains.) Laboratory strains *P. aeruginosa* PA01 (PA01) and *E. coli* K-12 (K12) were used as references to enable comparison with other studies. The strains were stored as frozen stocks in 33% *(v/v)* glycerol at − 80 °C until use.

To study interactions in dual-species biofilms, *P. aeruginosa* and *E. coli* strains isolated from the same patient were grown together, i.e. Tme04 with Tme12 (Pair412), Tmp05 with Tme13 (Pair513), Tmp06 with Tme14 (Pair614), Tmp07 with Tme15 (Pair715). In addition, a co-culture of the two lab strains PA01 with K12 (PairEP) was made as a reference. The strains were deposited at the Culture Collection of Gothenburg (CCUG) (https://www.ccug.se) during the time of publication of this work.

### Media and cultivation procedures

All strains were routinely cultivated on blood agar (BA) plates. Luria-Bertani broth (LB) and Iso-Sensitest Broth (ISO) from Thermo Scientific™ Oxoid™ were used for the growth of mono- and multispecies cultures in rich nutrient media. Artificial urine medium (AUM) prepared according to Brooks (1997)^84^ was used to study cultures at low nutrient content and growth conditions more similar to human urine, but standardised. To enable colony counting, selective agar plates were used to differentiate between colonies of *E. coli* and *P. aeruginosa* during growth. Two types of selective agar plates were used: urinary tract infection ChromoSelect agar from Millipore^®^ (UTI) and cysteine–lactose–electrolyte-deficient agar from Millipore^®^ (CLED). Growth appearance on these plates are shown in Supplementary information Figure S3. Physicochemical characterisation of strains, motility assays, and additional growth experiments using variants of these culture media are further described in the supplementary information.

### Biofilm assay in microplates

Fresh bacterial cultures grown on blood agar (BA) plates were used to make individual bacterial suspensions with an optical density at 600 nm wavelength (OD) of 1.3 in 0.9% *(w/v)* sterile NaCl. OD was measured using a WPA CO8000 Cell Density Meter. The biofilm assay was made in Sarstedt™ 96-well flat-bottom untreated plates (microplates). Corner and edge wells were filled with 200 µL of sterile water to reduce errors due to evaporation during the incubation. Two microplates were prepared for each medium condition: AUM, LB, and ISO. The bacterial suspension of each monoculture and co-culture was added to separate wells in microplates (6 replicates per sample) to give a starting OD = 0.1. In co-cultures, the *E. coli* and *P. aeruginosa* ratio was 1:1 by volume. The microplates were incubated at + 37 °C for 4 h. To exchange part of the medium and provide fresh nutrients, 150 µL of liquid medium from each well was discarded carefully without disturbing the formed biofilm and 150 µL of fresh warm media (+ 37 °C) was pipetted into each well carefully. After that, all plates were incubated for an additional 16 h at + 37 °C. (Typical colony appearance exemplified in Supplementary information Figure S21).

The crystal violet (CV) staining assay for biofilms previously reported by O’Toole GA (1998) was used to stain the biofilms described above after 16 h of cultivation^13^. All liquid in each well was carefully discarded using a pipette. Each well was, thereafter, incubated with 125 µL of a 0.1% *(w/v)* aqueous solution of CV for 10 min at room temperature. Then, the CV solution was removed, and each well was washed three times with 200 µL of 0.9% *(w/v)* NaCl. Stained biofilms in microplates were air-dried overnight at room temperature. To extract CV bound to biofilms, 150 µL of 33% *(v/v)* acetic acid was added to each well and incubated at room temperature for 10 min. Then, all liquid was transferred to new microplates. Plates were analysed in a multilabel counter Perkin Elmer Wallac 1420 VICTOR2™ at a wavelength of 600 nm.

### Urease testing

To examine the urease activity of the clinical strains, a previously published urease test protocol was used^85^. Each tube contained 5 mL of the agar test medium. Bacterial cultures with OD_600_ = 1.3 of each bacterial strain were prepared in 0.9% *(w/v)* NaCl from fresh BA-plates. Then, 20 µL of single bacterial culture was inoculated into test tubes. A volume of 10 µL of each bacterial suspension into tubes was used for pairs. Urease activity was evaluated visually and recorded by taking a photo of each test tube at different time points with a maximum incubation time of 120 h. The colour changes of the medium in the test tubes from yellow to purple indicated urease activity following alkalinisation (Supplementary information Figure S20). The *P. aeruginosa* PA01 strain was used as a positive control, and *E. coli* K12 strain was used as a negative control. Testing was performed three times.

### Viable counts

To estimate the number and ratio of viable cells of *P. aeruginosa* and *E. coli* in co-cultures, the viable counts of biofilms were recorded by counting colony-forming units per 1 mL (CFU/mL) on selective UTI plates^86^. Biofilms were cultivated as described above in AUM and ISO. After 20 h of total incubation at + 37 °C, 150 µL of supernatant was removed carefully using a multichannel pipette. As a washing step, 200 µL of sterile 0.9% *(w/v)* NaCl was carefully pipetted into each well and removed. Thereafter, 100 µL of sterile 0.9% *(w/v)* NaCl was pipetted into each well, and the biofilm detached by scraping with the tip of a single-channel pipette. The biofilms from each strain pair (24 wells per pair) were pooled, and OD was measured in glass tubes (OD_i_). Tubes with biofilms were adjusted to OD ≈ 1.0 (OD_a_) with sterile 0.9% *(w/v)* NaCl, and a 10-fold dilution series was prepared until the final dilutions were 10^− 5^ and 10^− 6^. Then, 100 µL of these final dilutions were transferred onto the UTI plates, and the bacterial suspension was spread by shaking with sterile glass beads. The plates were incubated at + 37 °C overnight. Colony-forming units (CFU/mL) were calculated for each plate considering the dilutions. Six plates were counted for each condition.

### Sample preparation for metabolomics

Bacterial cultures were grown in AUM and ISO in 96-well plates, following the growth conditions from the protocol for the biofilm assay in microplates above. From 12 wells (independent cultures) grown under the same experimental conditions, 100 µL of supernatant was carefully aspirated to avoid biofilm disruption and then transferred to clean 2 mL Eppendorf tubes. This process was repeated across all experimental conditions, resulting in 1.2 mL of bacterial culture per tube. Then, tubes were centrifuged at 15,000 rpm for 10 min, and 110 µL volumes of supernatant were aliquoted into fresh 1.5 mL tubes. These tubes with supernatant were labelled and stored in the freezer. Thereafter, the remaining supernatant was discarded from the original tubes, and 0.5 mL of NaCl 0.9% was added to wash the bacterial pellet. After centrifugation and discarding the washing solution, the tubes containing the bacterial pellet (planktonic cells) were frozen.

To collect the biofilm, the wells containing bacterial biofilms were washed twice with 200 µL of 0.9% NaCl, carefully avoiding the degradation of the biofilm. Then, 100 µL of 0.9% NaCl solution was added to each empty well containing biofilm. Scratching with a micropipette tip was done to detach the biofilm and release it into the solution, which was then transferred to a new 1.5 mL tube. After centrifugation and removal of the supernatant, the tubes containing biofilm cells were frozen. For AUM cultures three independent samples pooled from 12 wells were used, and for ISO cultures, two independent samples pooled from 12 wells for gas chromatography/mass spectrometry (GC–MS).

### Gas chromatography-mass spectrometry

Metabolites from the bacterial cell cultures and supernatants were analysed using GC–MS as previously described^87,88^.

Spent media samples (50 µL), biofilm pellets and planktonic cell pellets collected in a previous step were extracted separately. Each sample was extracted in a 1.5 mL Eppendorf tube (Sarstedt™) with 450 µL of extraction mixture: MeOH/H_2_O in a ratio 90:10 *(v/v)* (Fisher Chemicals™) including internal standards (IS, 7 ng/µL) using tungsten beads (Qiagen) and a bead mill to facilitate the release of metabolites. After that, samples were incubated on ice for 2 h and centrifuged for 10 min at 4 °C, at 14,000 rpm (Hettich, 220/220R, Germany). The supernatant containing extracted metabolites was transferred to a fresh tube and kept at − 20 °C.

Before derivatisation, 100 µL of the supernatant was transferred to GC-vials (Scantec Nordic/Thermo Fisher Scientific). The samples were evaporated for 2 h using Genevac™ miVac Quatro Concentrator until dryness. For derivatisation, 15 µL of methoxamine was added to the vials, then they were shaken for 10 min using Vortex mixer multi (VWR) and incubated at 70 °C for 1 h in an oven (Memmert GmbH/Thermo Fisher Scientific). The samples were left at room temperature for 16 h. Further, 30 µL of 50:50 mix *(v/v)* of N-methyl-N-(trimethylsilyl)trifluoroacetamide (MSTFA) % and heptane (including methyl stearate 15 ng/mL) was added to each sample, vortexed and incubated at room temperature for 1 h before analysis. For alkane series standards, a mix of C8–C20 (10 µL), C21–C40 (10 µL) and heptane (30 µL), all purchased from Sigma-Aldrich/Merck, was used.

The samples were run using Pegasus^®^ BT GC–TOFMS, Benchtop Gas Chromatography Time-of-Flight Mass Spectrometer. A fused silica capillary column (10 m × 0.18 mm × 0.18 μm internal diameter) with a stationary phase DB5-MS (0.18 μm) and helium as a mobile phase was used. An aliquot of 0.5 µL of each sample was collected using L-PAL3, LECO autosampler and injected into an Agilent 7809B gas chromatograph coupled to a mass analyser. Quality control samples were prepared by pooling 50 µL of extracted samples. Supernatant, planktonic cell and biofilm cell samples had three technical replicates for samples grown in AUM and two technical replicates for samples grown in ISO.

### Raw data pre-processing

The raw metabolomic data obtained after GC–MS analysis was exported as NetCDF files. Baseline correction, chromatogram alignment, peak picking and preliminary annotation through comparison to in/house libraries was done using the in-house software SMC–RDA 3.996 (Swedish Metabolomics Centre – Raw Data Analysis). After this, the data was manually curated by excluding all distorted peaks. Thereafter, each metabolite annotated in SMC–RDA was validated using the NIST MS 08 search (National Institute of Standard and Technology, USA) against public metabolite libraries. Annotation was confirmed when the NIST MS match score was larger than 700. Metabolites that had low match scores were excluded from further analysis. Peak areas of metabolites were used to infer quantitative relationships between sample groups. To compensate for potential differences in sample biomass, peak areas for planktonic and biofilm cell samples were normalised to average peak intensity for all metabolites in a given sample as previously described by Ilchenko et al., 2024^87^.

### Multivariate data analysis

To analyse the metabolomic data after pre-processing, multivariate data analysis (MVDA) was performed using SIMCA^®^ 18.0.0 software (Umetrics AB/Sartorius, Umeå, Sweden). The analysis included Principal Component Analysis (PCA) and Orthogonal Partial Least Squares Discriminant Analysis (OPLS-DA) as the main tools, as previously described^89–91^. The resulting models are presented as supplementary information Figures S4-S19. Based on these analyses, heat maps presented in Figs. 4, 5, 6, 7, 8 and 9 were developed, as described below.

PCA is an unsupervised method used to reduce the dimensionality of the multivariate data while retaining most of the variation^89^. The original variables are transformed into principal components (PCs) capturing the maximum variance in the data. Data were pre-processed by unit variance scaling (UV-scaling) and mean-centring. The number of PCs was determined based on the cumulative explained variance representing a high proportion of the total variance (first two components), thereby capturing the dataset’s primary structure. Sample clustering was visualised using a score plot, while a loading plot highlighted the contribution of individual metabolites to the sample distribution observed in the score plot.

OPLS-DA is a supervised method aimed at finding separation between pre-defined sample classes. A unique feature of the OPLS methodology is the ability to separate the systematic variation in the predictor matrix (X) into predictive variation correlated with the response (Y, defined sample classes) and orthogonal variation uncorrelated with Y^90,91^. Thus, facilitating interpretation of the complex metabolomics data. Data preprocessing was performed similarly to PCA, using UV-scaling and mean-centring. The number of predictive and orthogonal components was optimised through internal cross-validation.

Model quality was assessed using several parameters: R2X (proportion of variation in X explained by the model, with values > 0.5 indicating a good model), R2Y (proportion of variation in Y explained by the model, with values > 0.7 indicating a good fit) and Q2 (predictive ability, with values > 0.5 suggesting strong predictive performance). Variable Importance in Projection (VIP) scores were used to evaluate the significance of each variable, with scores > 1.0 indicating important contributions to class separation.

S-plots were generated for each OPLS-DA model to identify significant variables contributing to class separation by plotting predictive loadings *p* against correlation coefficients *p(corr)*. Metabolites located at the plot corners (both positive and negative) were considered significant, while those near the centre — less significant. Thresholds of |*p*| > 0.15 and |*p(corr)*| > 0.5 were applied to select valuable metabolites contributing to group separation^89,92^.

The Shared and Unique Structures (SUS) plot is a graphical tool used to compare two OPLS-DA models (e.g., *E. coli* vs. Pair and *P. aeruginosa* vs. Pair) by plotting the correlation coefficients *p(corr)* from each model^93^. This approach helps identify common and unique features between the models. For each SUS-plot, *p(corr)* values from the OPLS-DA model of *P. aeruginosa* strains versus Pairs were plotted on the X-axis, while those from the *E. coli* strains versus Pairs model were plotted on the Y-axis. Variables near the diagonal represent features shared between both models, while variables farther from the diagonal indicate features unique to one of the models.

### Choosing of key metabolites

After the multivariate analysis, key metabolites were identified for each bacterial group (mono- and co-cultures of *E. coli* and *P. aeruginosa*) across supernatants, planktonic and biofilm cells grown in AUM and ISO media. Metabolite identification was based on the following criteria: *p* — loading weights indicating the contribution of each variable to the predictive component; *p(corr)* — correlation coefficient representing the relationship between each variable and bacterial class separation; VIP — variable importance of projection, reflecting the metabolite’s importance to class separation; VIPcvSE or VIPcvSE × 2.44693 (Variable Importance in Projection with Cross-Validation Standard Error) is a modified VIP score incorporating cross-validation standard error, indicating stability if VIP scores.

Significance thresholds were set as follows: |*p*| > 0.15 — a higher value reflects the metabolite’s contribution to group separation; |*p(corr)*| > 0.5 — a greater value emphasizes the metabolite’s correlation with specific bacterial groups; VIP > 1 — indicates the significance of the metabolite in class separation; VIPcvSE — assesses the consistency of the VIP score during cross-validation, with a lower value indicating greater stability and robustness of the VIP score; VIP/VIPcvSE ratio > 2 — a larger value suggests the reliability of the variable (metabolite) importance.

To define the key metabolites for each bacterial group (*E. coli* monoculture, *P. aeruginosa* monoculture, *E. coli* and *P. aeruginosa* co-culture) within each sample type (supernatant, planktonic, and biofilm cells) grown in ISO and AUM, each metabolite was scored based on four criteria: *p*,* p(corr)*, VIP, and VIP/VIPcvSE. Since the chosen parameters do not have universally defined thresholds and can vary depending on the strictness of data analysis performed, the scoring was done as a sum of these four criteria, each multiplied by its respective weight of importance (criterion value × weight). This scoring approach, described below, allowed us to filter and identify relevant metabolites that are important for sample separation. Importantly, this approach is not overly strict, and by combining the effects of all criteria, it reduces the risk of overlooking metabolites that may individually fall just outside a specific threshold but, when considered collectively, still contribute meaningfully to group separation.

The weight for |*p*| was set to 0.75, as metabolites with lower |*p*| < 0.15 were considered relevant. The weight for |*p(corr)*| was set to 1.25, since this parameter is important for demonstrating strong correlations with bacterial groups and for observing significant differences between groups. The weight of VIP was set to 1.5, reflecting the importance of the metabolite in distinguishing between bacterial groups and its role in the model. The weight for VIP/VIPcvSE ratio was set to 1.0, as the smaller VIPcvSE indicates greater stability of the VIP score. A larger ratio suggests that the metabolite is more stable and not overfitted in the model, confirming it is not a false positive. The scoring formula was as follows:

$$\:Score\:=\:\left|p\right[1\left]\right|\:\times\:\:0.75\:+\:\left|p\right(corr\left)\right|\:\times\:\:1.25\hspace{0.17em}+\hspace{0.17em}VIP\:\times\:\:1.5\hspace{0.17em}+\hspace{0.17em}VIP/VIPcvSE\:\times\:\:1$$

Finally, all metabolites were evaluated based on their final score. Metabolites with a score > 4 were considered of high importance. Metabolites with scores > 2.5 were considered of moderate importance. Metabolites with scores < 2.5 were considered of low importance.

Metabolites with high |*p(corr)*| values in SUS-plots and high or moderate scores were interpreted as highly correlated to specific bacterial groups, suggesting they were likely produced primarily by those groups.

### Statistical analysis

After multivariate data analysis (MVDA), statistical evaluations and heatmap generation were done using a custom Python based pipeline implementing adaptive statistical testing named COWTEA (CAUTI Omics Workflow for Targeted metabolite Analysis) using libraries *pandas*,* matplotlib*,* numpy*,* seaborn*,* scipy*,* statsmodels*, and *scikit-posthocs*, alongside with GraphPad Prism 8. A two-tailed unpaired t-test compared test conditions with controls (e.g., viable counts). Two-way ANOVA evaluated biofilm formation differences across bacterial strains and media.

Extracellular and intracellular metabolite profiles were analysed using Cowtea v1.0.0 (available at https://github.com/sokoljator/CAUTI-metabolomics, in the repository choose script Cowtea_v1.0.0.py). Medium control (without bacteria) was used for analysing extracellular metabolites. For intracellular metabolites, the averaged measurement from reference strains (K12 and PA01) was used, with standard deviations calculated as √((sd1²+sd2²)/2). Transformation methods (log, square root, cube root, Box-Cox, generalised log, inverse) were assessed using SciPy’s statistics, and the method yielding the lowest percentage of non-normally distributed metabolites (Shapiro-Wilk test, *p* > 0.05) was selected further.

Data distributions for each metabolite were evaluated for normality via Shapiro-Wilk tests and variance homogeneity with Levene’s tests (both from SciPy). Statistical tests were chosen based on characteristics: one-way ANOVA for normally distributed data with equal variances, Welch’s ANOVA for normally distributed data with unequal variances, or Kruskal-Wallis for non-normally distributed data. Significant results (*p*-value < 0.05) were further processed with post-hoc tests: Tukey’s HSD after ANOVA, Games-Howell following Welch’s ANOVA, or Dunn’s test after Kruskal-Wallis. Analysis included hierarchical comparisons, which included global pairwise strain comparisons, biological group tests (clinical isolates versus respective reference strains), and between-group comparisons (*E. coli* vs. control, *P. aeruginosa* vs. control, Pairs vs. control). Multiple comparison corrections were applied using the false discovery rate (FDR) method (Benjamini-Hochberg procedure).

Metabolite significance was determined through two criteria: statistical significance between control and test strains (FDR-corrected p-value < 0.05) and absolute log₂ fold-change > 0.75 (> 68% increase or > 40% decrease) in at least 33% of non-control strains (calculated with NumPy). Clustered heatmaps of log2 fold-changes of peak area were generated using Seaborn’s cluster map. Metabolites meeting both criteria were unmarked (dual-significant), indicating robust metabolic changes.While those meeting only statistical significance were marked with asterisks (*), representing subtle effects of limited biological magnitude. That those meeting only fold-change (FC) thresholds were marked with hashtags (#), indicating biologically substantial changes covered by high variability. These heatmaps represent log2FC for metabolites in all samples compared to controls. The medium control was used for the samples in the spent medium. The averaged reference strains K12 and PA01 were used for planktonic and biofilm cell samples (one per planktonic and one per biofilm cell sample). A log2FC cutoff of |0.75| was applied to highlight biologically relevant changes (> 68% increase for log2FC > 0.75; > 40% decrease for log2FC < −0.75).

## Supplementary Information

Below is the link to the electronic supplementary material.

Supplementary Material 1

## Data Availability

The datasets used and/or analysed during the current study are available from the corresponding author on reasonable request.
