# Correction: Metabolic interactions between bacterial co-isolates from catheter-associated urinary tract infections

**DOI:** 10.1038/s41598-026-39740-9

**Published:** 2026-02-16

**Authors:** Dmytro Sokol, Olena Rzhepishevska, Iryna Marynova, Tor Monsen, Henrik Antti, Madeleine Ramstedt

**Affiliations:** 1 Department of Chemistry, Umeå Centre of Microbial Research, Umeå University, Umeå, Sweden; 2 Odesa I. I. Mechnikov National University, Odesa, Ukraine; 3 Department of Clinical Microbiology, Umeå University, Umeå, Sweden

Correction to : *Scientific Reports* 10.1038/s41598-025-33855-1, published online 14 January 2026

The original version of this Article contained errors in the legend of Figure 4, 5, 6, 7, 8 and 9 where the following sentence was omitted: “(p-value < 0.05 after FDR correction) are marked with an asterisk (*), while those with a significant fold-change (absolute log2FC > 0.75)”. As a result, the legends of the figures have been corrected as follows:
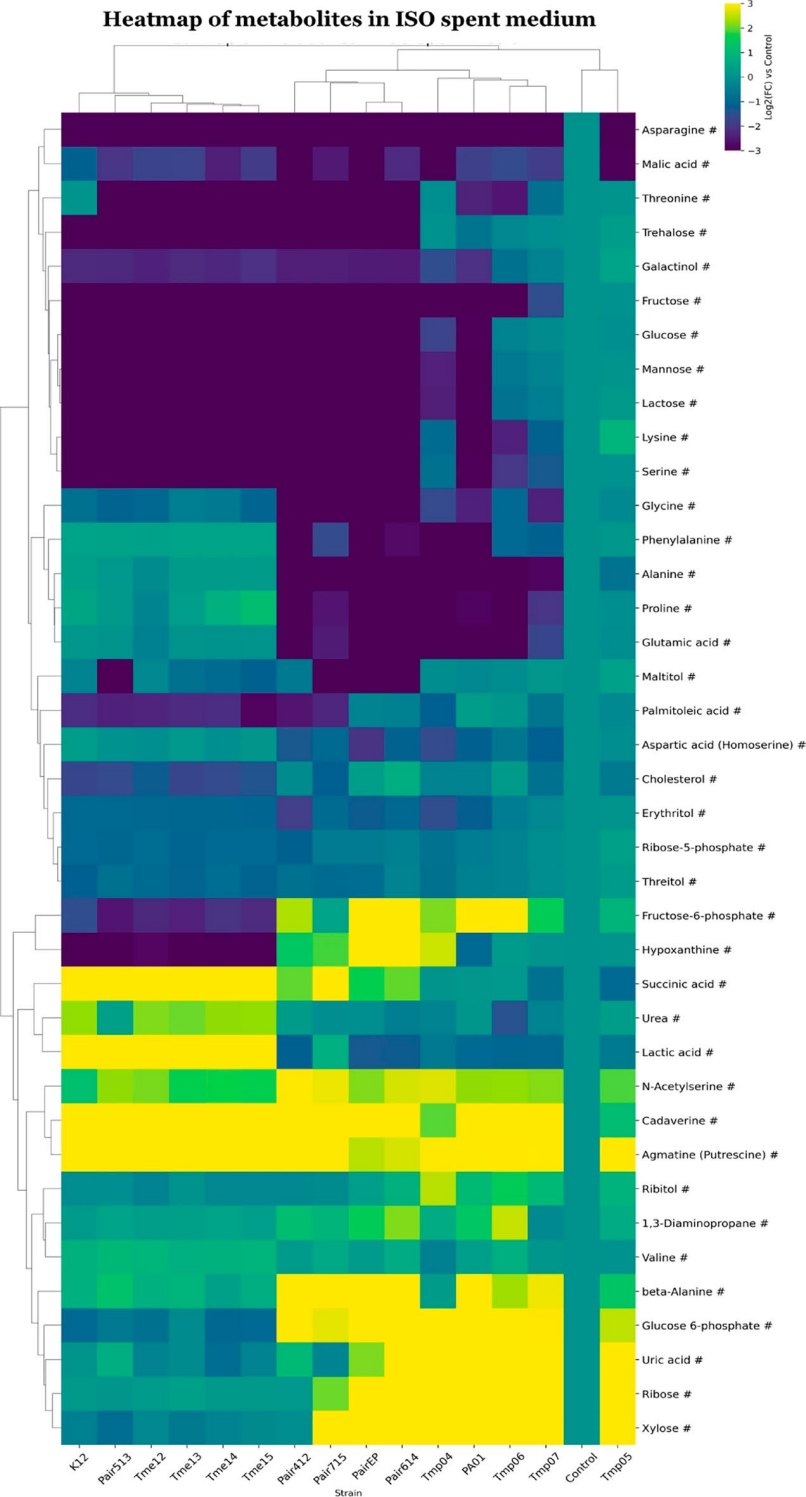


“**Figure 4. Clustered heatmap of significant metabolites in ISO spent medium.**

Clustered heatmap of significant metabolites in ISO spent medium. Metabolites are marked based on their significance: those that are statistically significant (p-value 0.75) are marked with a hashtag (#). Metabolites that meet both criteria are left unmarked. The FC for each metabolite in bacterial samples was calculated relative to its peak area in the medium control sample. Heatmap was generated using Cowtea v1.0.0 (repository link https://github.com/sokoljator/CAUTI-metabolomics).”

now reads:

“**Figure 4. Clustered heatmap of significant metabolites in ISO spent medium.**

Metabolites are marked based on their significance: those that are statistically significant (p-value < 0.05 after FDR correction) are marked with an asterisk (*), while those with a significant fold-change (absolute log2FC > 0.75) are marked with a hashtag (#). Metabolites that meet both criteria are left unmarked. The FC for each metabolite in bacterial samples was calculated relative to its peak area in the medium control sample. Heatmap was generated using Cowtea v1.0.0 (repository link https://github.com/sokoljator/CAUTI-metabolomics)”.
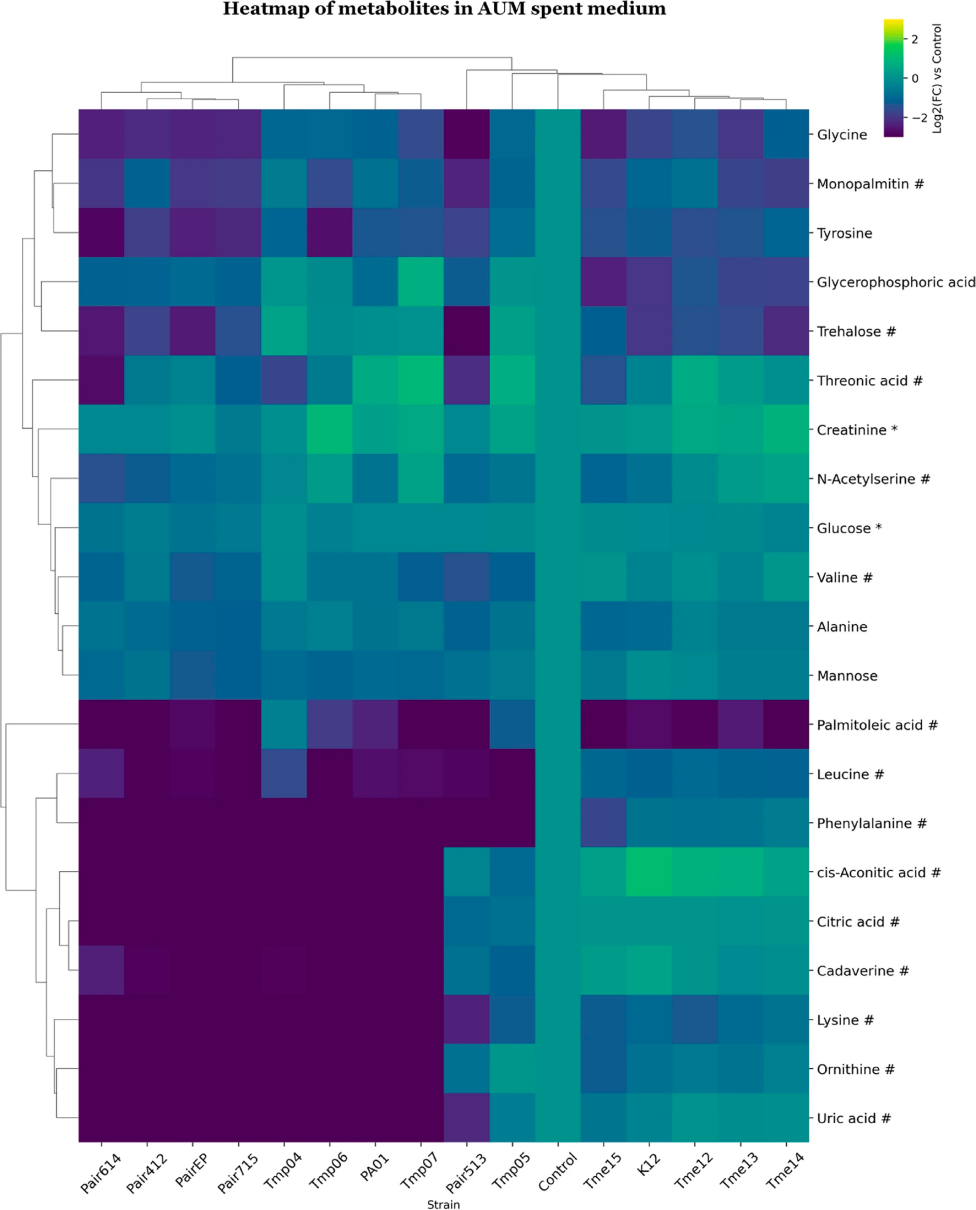


“**Figure 5. Clustered heatmap of significant metabolites in AUM spent medium.**

Clustered heatmap of significant metabolites in AUM spent medium. Metabolites are marked based on their significance: those that are statistically significant (p value 0.75) are marked with a hashtag (#). Metabolites that meet both criteria are left unmarked. The FC for each metabolite in bacterial samples was calculated relative to its peak area in the medium control sample. Heatmap was generated using Cowtea v1.0.0 (repository link https://github.com/sokoljator/CAUTI-metabolomics)”.

now reads:

“**Figure 5. Clustered heatmap of significant metabolites in AUM spent medium.**

Metabolites are marked based on their significance: those that are statistically significant (p value < 0.05 after FDR correction) are marked with an asterisk (*), while those with a significant fold-change (absolute log2FC > 0.75) are marked with a hashtag (#). Metabolites that meet both criteria are left unmarked. The FC for each metabolite in bacterial samples was calculated relative to its peak area in the medium control sample. Heatmap was generated using Cowtea v1.0.0 (repository link https://github.com/sokoljator/CAUTI-metabolomics )”.
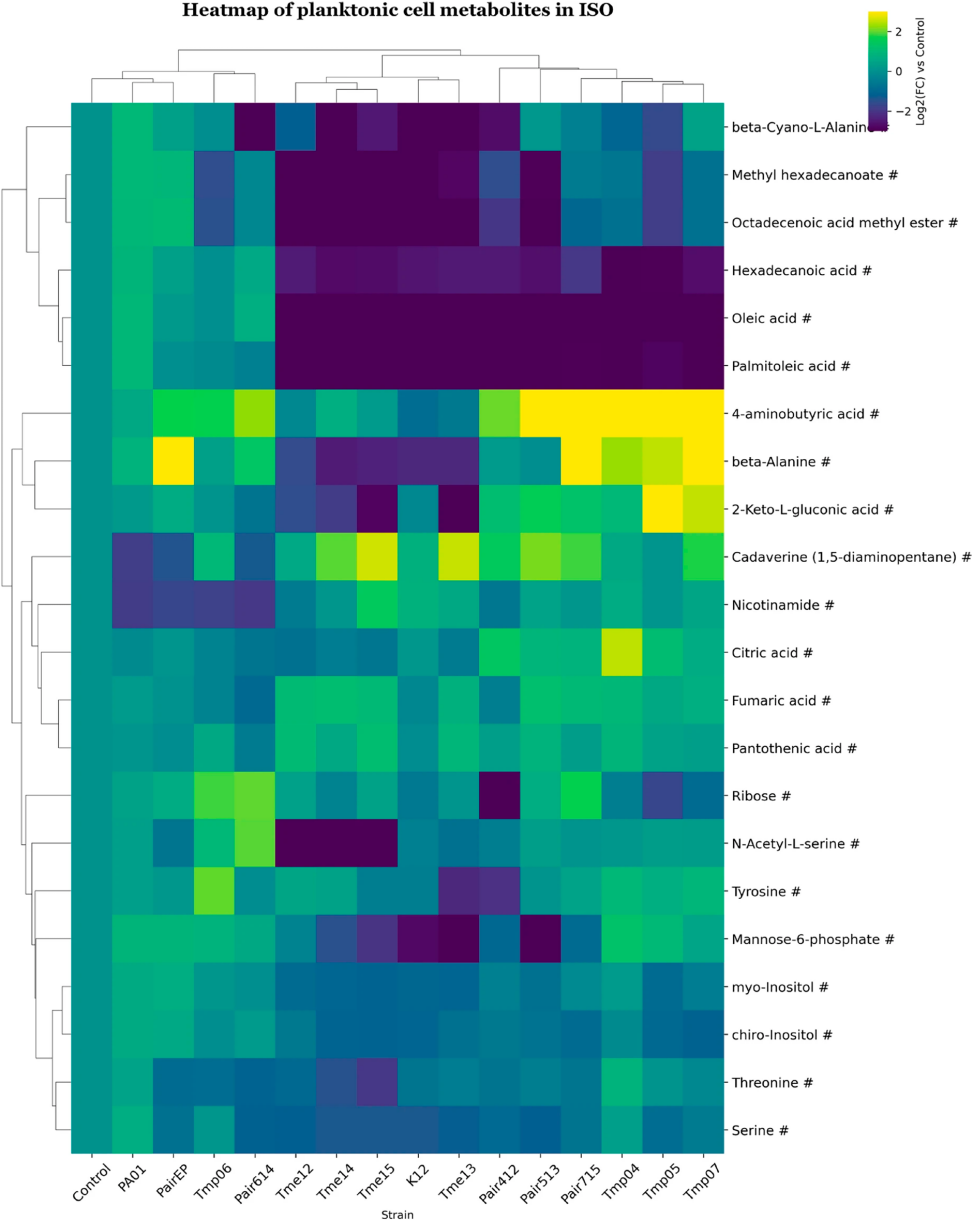


“**Figure 6. Clustered heatmap of significant metabolites in mono- and co-cultures of planktonic cell samples in ISO.**

Clustered heatmap of significant metabolites in mono- and co-cultures of planktonic cell samples in ISO. Metabolites are marked based on their significance: those that are statistically significant (p-value 0.75) are marked with a hashtag (#). Metabolites that meet both criteria are left unmarked. The FC for each metabolite in bacterial samples was calculated relative to its peak area in the control (average metabolite composition of K12 and PA01). Heatmap was generated using Cowtea v1.0.0 (repository link https://github.com/sokoljator/CAUTI-metabolomics)”.

now reads:

“**Figure 6. Clustered heatmap of significant metabolites in mono- and co-cultures of planktonic cell samples in ISO.**

Metabolites are marked based on their significance: those that are statistically significant (p-value < 0.05 after FDR correction) are marked with an asterisk (*), while those with a significant fold-change (absolute log2FC > 0.75) are marked with a hashtag (#). Metabolites that meet both criteria are left unmarked. The FC for each metabolite in bacterial samples was calculated relative to its peak area in the control (average metabolite composition of K12 and PA01). Heatmap was generated using Cowtea v1.0.0 (repository link https://github.com/sokoljator/CAUTI-metabolomics)”.
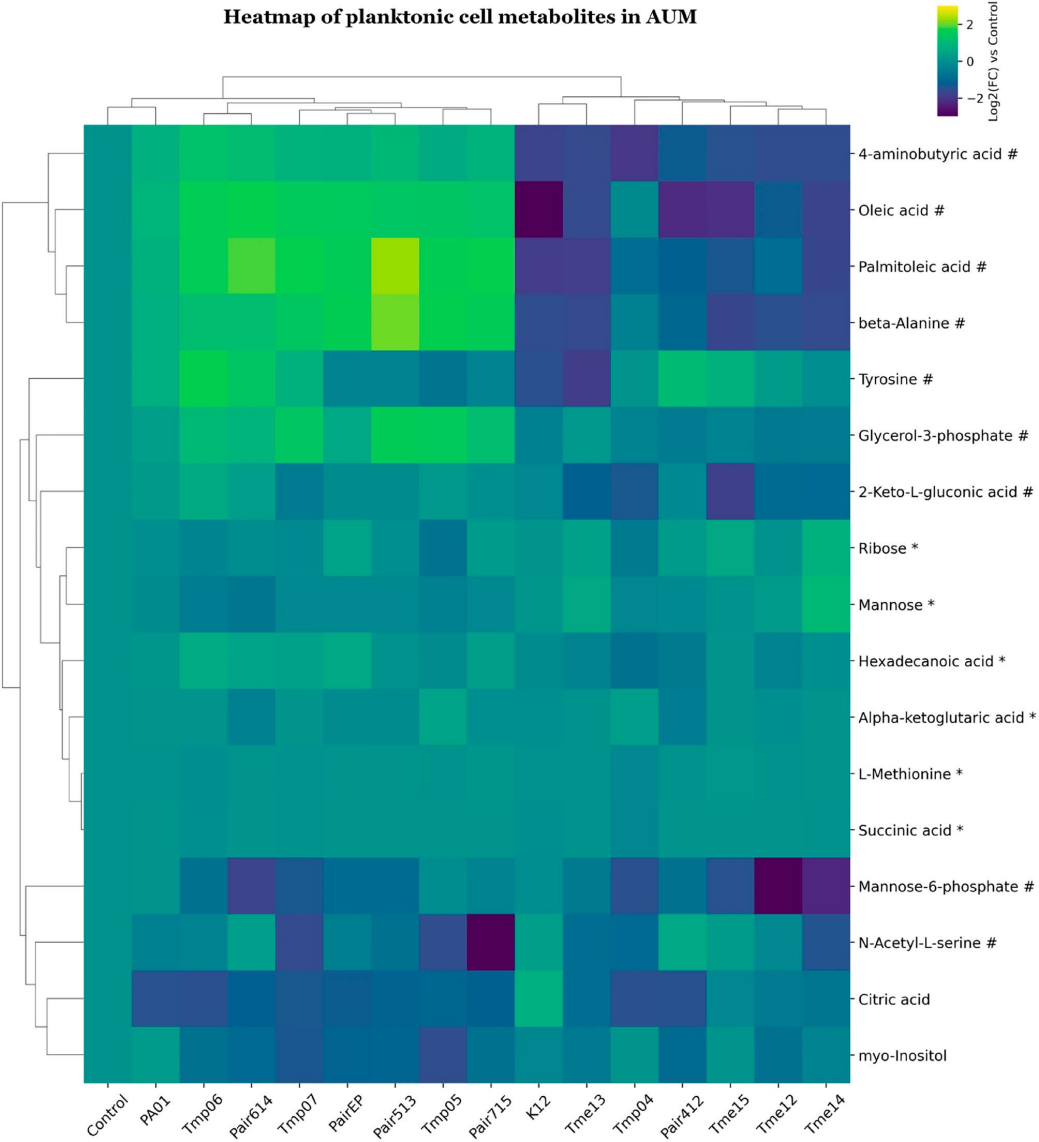


“**Figure 7. Clustered heatmap of significant metabolites in mono- and co-cultures of planktonic cell samples in AUM.**

Clustered heatmap of significant metabolites in mono- and co-cultures of planktonic cell samples in AUM. Metabolites are marked based on their significance: those that are statistically significant (p-value 0.75) are marked with a hashtag (#). Metabolites that meet both criteria are left unmarked. The FC for each metabolite in bacterial samples was calculated relative to its peak area in the control (average metabolite composition of K12 and PA01). Heatmap was generated using Cowtea v1.0.0 (repository link https://github.com/sokoljator/CAUTI-metabolomics)”.

now reads:

“**Figure 7. Clustered heatmap of significant metabolites in mono- and co-cultures of planktonic cell samples in AUM.**

Metabolites are marked based on their significance: those that are statistically significant (p-value < 0.05 after FDR correction) are marked with an asterisk (*), while those with a significant fold-change (absolute log2FC > 0.75) are marked with a hashtag (#). Metabolites that meet both criteria are left unmarked. The FC for each metabolite in bacterial samples was calculated relative to its peak area in the control (average metabolite composition of K12 and PA01). Heatmap was generated using Cowtea v1.0.0 (repository link https://github.com/sokoljator/CAUTI-metabolomics)”.
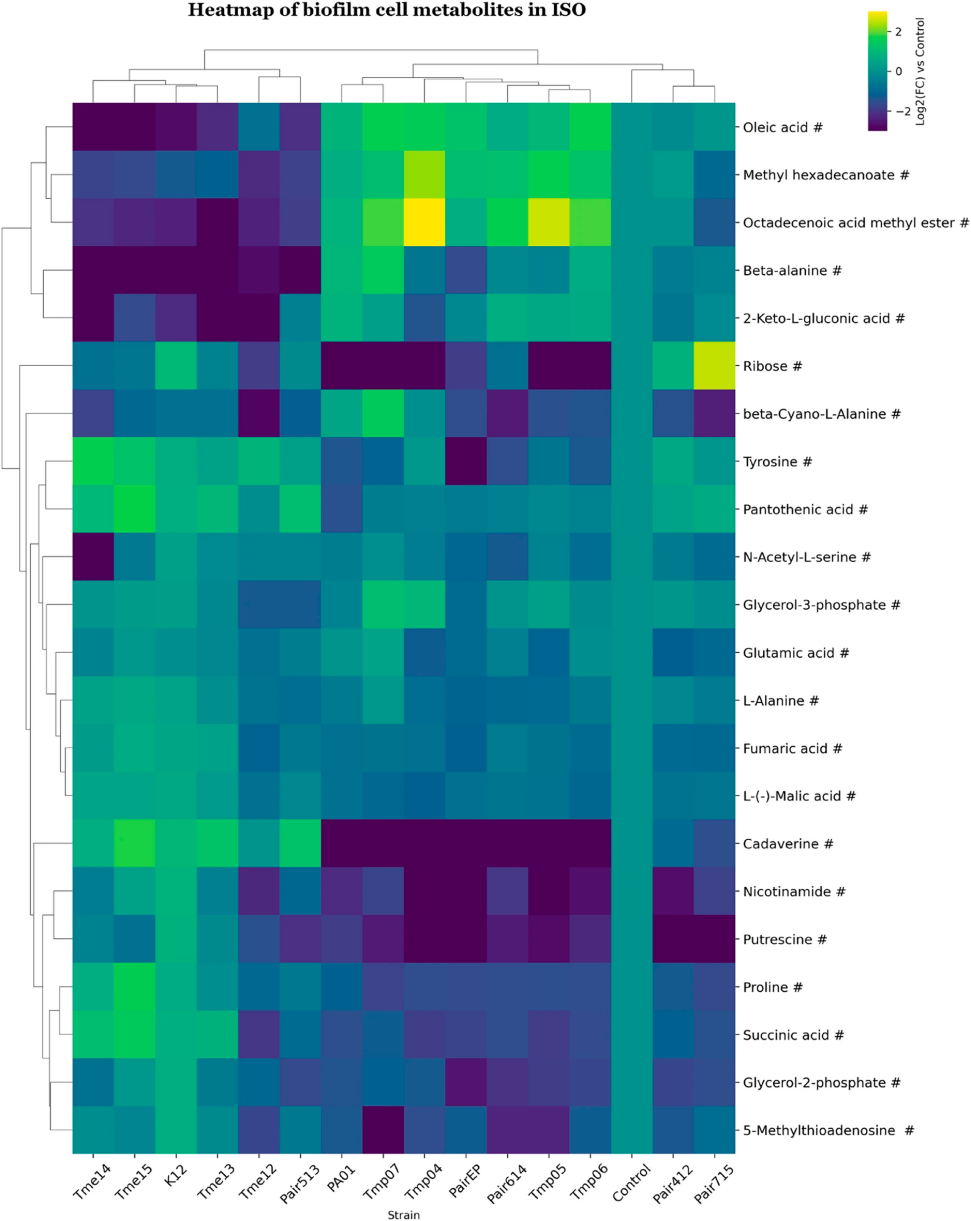


**“Figure 8. Clustered heatmap of significant metabolites in mono- and co-cultures of biofilm cell samples in ISO.**

Clustered heatmap of significant metabolites in mono- and co-cultures of biofilm cell samples in ISO. Metabolites are marked based on their significance: those that are statistically significant (p-value 0.75) are marked with a hashtag (#). Metabolites that meet both criteria are left unmarked. The FC for each metabolite in bacterial samples was calculated relative to its peak area in the control (average metabolite composition of K12 and PA01). Heatmap was generated using Cowtea v1.0.0 (repository link https://github.com/sokoljator/CAUTI-metabolomics)”.

now reads:

**“Figure 8. Clustered heatmap of significant metabolites in mono- and co-cultures of biofilm cell samples in ISO.**

Metabolites are marked based on their significance: those that are statistically significant (p-value < 0.05 after FDR correction) are marked with an asterisk (*), while those with a significant fold-change (absolute log2FC > 0.75) are marked with a hashtag (#). Metabolites that meet both criteria are left unmarked. The FC for each metabolite in bacterial samples was calculated relative to its peak area in the control (average metabolite composition of K12 and PA01). Heatmap was generated using Cowtea v1.0.0 (repository link https://github.com/sokoljator/CAUTI-metabolomics)”.
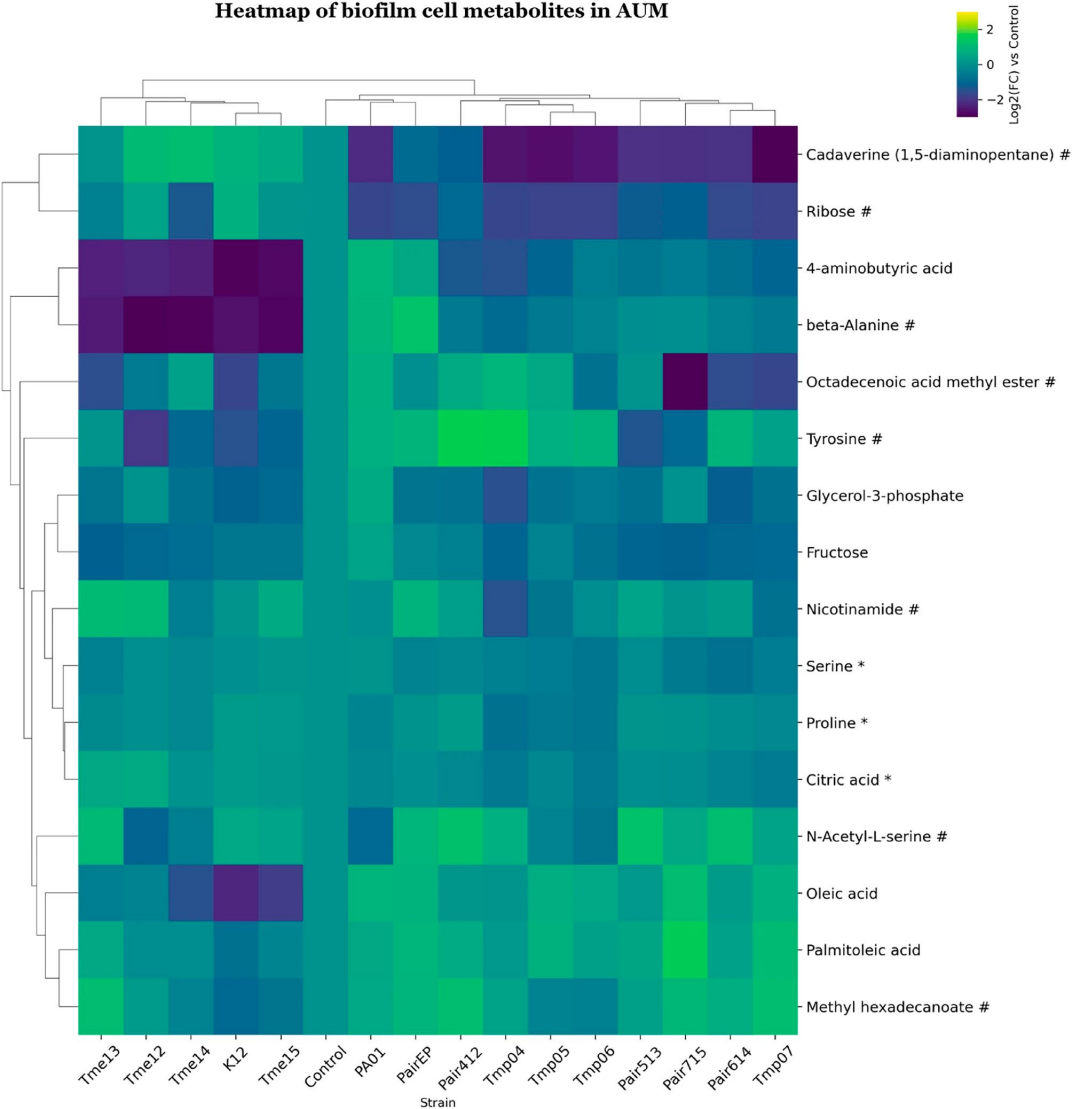


**“Figure 9. Clustered heatmap of significant metabolites in mono- and co-cultures of biofilm cell samples in AUM.**

Clustered heatmap of significant metabolites in mono- and co-cultures of biofilm cell samples in ISO. Metabolites are marked based on their significance: those that are statistically significant (p-value 0.75) are marked with a hashtag (#). Metabolites that meet both criteria are left unmarked. The FC for each metabolite in bacterial samples was calculated relative to its peak area in the control (average metabolite composition of K12 and PA01). Heatmap was generated using Cowtea v1.0.0 (repository link https://github.com/sokoljator/CAUTI-metabolomics) ”.

now reads:

**“Figure 9. Clustered heatmap of significant metabolites in mono- and co-cultures of biofilm cell samples in AUM.**

Metabolites are marked based on their significance: those that are statistically significant (p-value < 0.05 after FDR correction) are marked with an asterisk (*), while those with a significant fold-change (absolute log2FC > 0.75) are marked with a hashtag (#). Metabolites that meet both criteria are left unmarked. The FC for each metabolite in bacterial samples was calculated relative to its peak area in the control (average metabolite composition of K12 and PA01). Heatmap was generated using Cowtea v1.0.0 (repository link https://github.com/sokoljator/CAUTI-metabolomics)”.

The original Article has been corrected.

