# Supplementary material for: Metabolic interactions between bacterial co-isolates from catheter-associated urinary tract infections
Source: Sci Rep. 2026 Jan 14;16:2061. doi: 10.1038/s41598-025-33855-1 (PMC12808099; doi:10.1038/s41598-025-33855-1)
Supplement: Supplementary file 1 — Supplementary Material 1 [file 41598_2025_33855_MOESM1_ESM.pdf]

SUPPLEMENTAL INFORMATION

For manuscript

Metabolic interactions between bacterial co-isolates from catheter-associated urinary tract infections

Dmytro Sokol[1], Olena Rzhepishevska[1], Iryna Marynova[1,2], Tor Monsen[3], Henrik Antti[1], Madeleine Ramstedt*[1]

1. Department of Chemistry, Umeå Centre of Microbial Research, Umeå University, Umeå, Sweden
2. Odesa I. I. Mechnikov National University, Odesa, Ukraine
3. Norrland's University Hospital (NUS), Umeå, Sweden

*=corresponding author (madeleine.ramstedt@umu.se)

Supplemental methodology

Cell size and zeta potential measurement

Bacterial suspensions were prepared from colonies collected from an overnight bacterial culture on blood agar (incubated at 37 °C) suspended in sodium phosphate buffer at pH 7.4. The obtained suspension was prepared to have an optical density (OD_{600}) measured at 600 nm. Glass tubes with bacterial suspensions were kept on ice to avoid cell proliferation before measurement. Zeta potential and cell size were measured with ZetaSizer Nano ZS, Malvern Instruments Ltd. Three biological replicas with three technical replicas were performed for each strain.

Hydrophobicity

The microbial adhesion to hexadecane (MATH) assay was used to determine hydrophobicity [1]. Bacterial suspension was prepared from overnight bacterial cultures on blood agar that were suspended in 1 mL of PBS buffer (pH = 7.4). A few drops of bacterial suspension were added into a tube with 3 mL PBS buffer to give OD_{600} = 0.5. As a reference, the PBS buffer was used before adding the bacterial suspension. A volume of 1 ml of hexadecane was added to the bacterial suspension, and the two phases were shaken vigorously on a vortex for 1 min at room temperature. The tube was left standing in a test tube rack until

two layers were separated. Thereafter, OD_{600} was re-measured (A). Three biological replicas were performed for each sample. Hydrophobicity was calculated according to the formula:

$$\text{Hydrophobicity}\,\% = \frac{A0 - A}{A0} \times 100$$

where *A0* — optical density of the initial cell suspension;

A — optical density of the aqueous phase after agitation with hexadecane.

Motility

Motility was determined using an assay with agar plates of different densities. A solution with 20 % Iso-sensitest medium was prepared according to the manufacturer's instructions. After that, agar was added to different final concentrations depending on the type of motility assay. The solutions were autoclaved and carefully poured into culture plates (25 mL), avoiding the formation of air bubbles, etc., that could influence the motility. A percentage of 0.3 % agar was used for swimming[2,3], 0.5 % for swarming [4,5] and 1 % for twitching[6,7].

The bacterial suspension was from an overnight bacterial culture on blood agar, suspended in 3 mL of phosphate-buffered saline (PBS) to give an OD_{600} = 1.0. Three replicas were performed for each motility. For each type of motility, 5 µL of bacterial suspension was used. For swarming, a drop of suspension was placed on top of the agar at the centre of the plate and left to dry. For twitching, the pipette was stabbed into the agar, and the suspension was inserted at the bottom of a Petri dish. For swimming, a drop of suspension was injected in the middle of the agar. Care was taken to avoid accidental injection on the agar plate surface that could lead to unwanted swarming motility. The plates were incubated at 37°C for 24 h without inverting the culture plates. The area of the growth zone was recorded as a measure of motility, photos of every agar plate were taken, and the area was measured using the Analysing Digital Images software (John Pickle and Alan Gould, 2011). In case of twitching, the agar was carefully removed to improve visualisation, and the culture at the bottom was stained with 0.1 % crystal violet water solution for 4–5 min at room temperature. After that, the crystal violet solution was removed, and the bottom of the Petri dish was washed with water. Swarming was judged binary as present or not.

Measuring pH after growth in liquid culture

Mono- and dual-species bacterial cultures were grown in AUM with urea concentrations of 10 g/L and 15 g/L to measure pH after cultivation. Glass tubes containing sterile 0.9 % (w/v) NaCl were prepared, adjusting the OD_{600} to 1.3 for each strain. Separate glass tubes with 3 mL of each medium were prepared in triplicate for each strain. An aliquot of 12 µL of bacterial culture from the NaCl tubes (OD_{600} = 1.3) was transferred into the glass tubes containing media. The tubes were vortexed and incubated at 37°C without shaking for 30 h. Then, bacterial suspensions were transferred into 15 mL plastic tubes, and pH was measured using a Mettler Toledo SevenEasy™ pH meter S20. Three replicates for each strain and their pairs in each medium were analysed.

Biofilm in the presence of different levels of urea

Biofilm formation of clinical strains grown in AUM containing higher urea concentrations (10 g/L, 15 g/L, 25 g/L) was investigated in both monocultures and pairs. The analysis was conducted using the crystal violet (CV) staining assay to quantify biofilm biomass under these conditions.

Supplementary results and discussion

Several experiments were performed to characterise the phenotype of the clinical isolates and compare them with the reference strains. Physicochemical properties such as cell hydrophobicity, zeta potential, aggregation behaviour and motility were investigated (**Figure S1**).

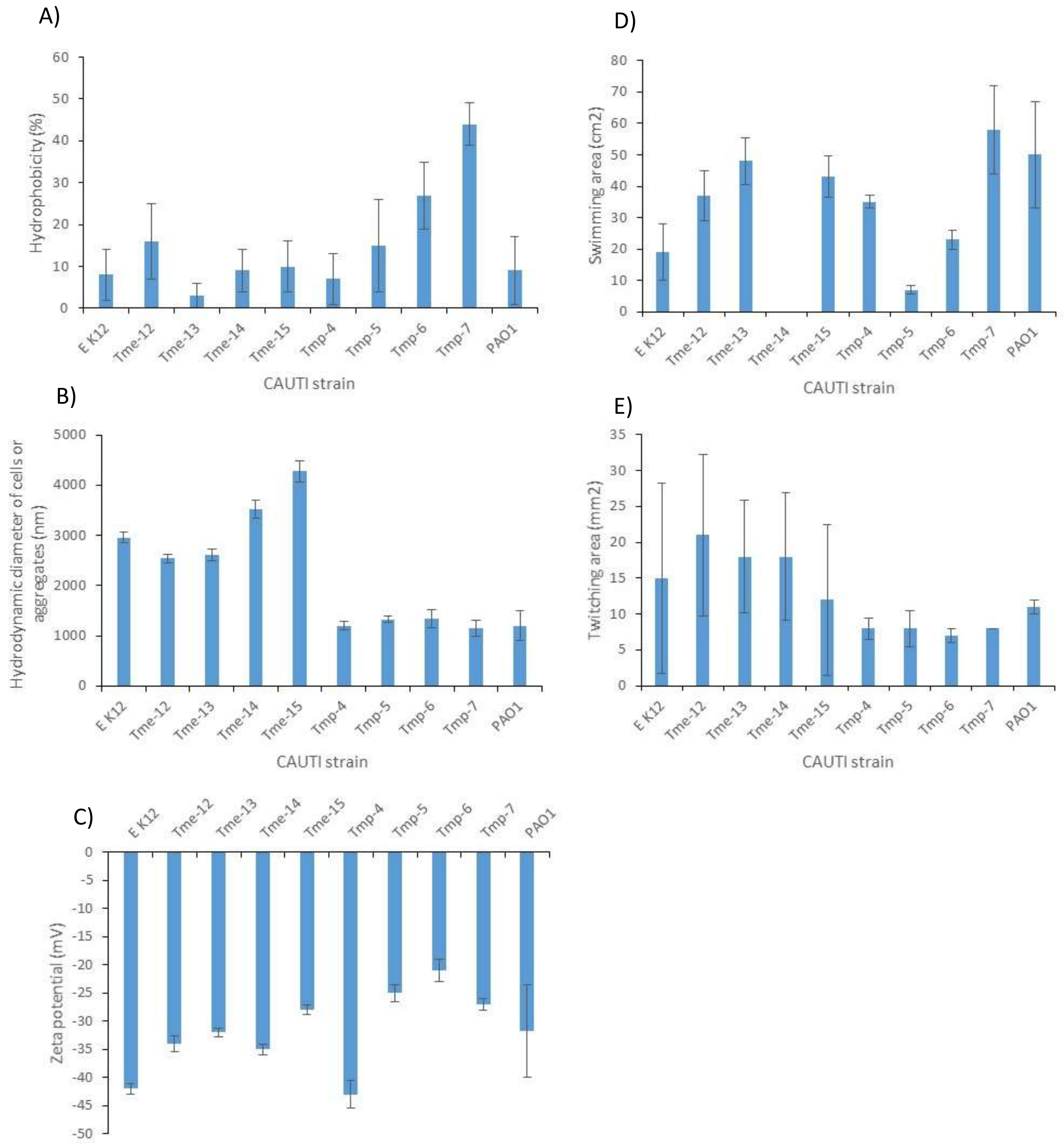

Figure S1. Physiochemical properties of CAUTI-strains.

a) Hydrophobicity, b) hydrodynamic diameter of cells or cell aggregates formed in buffer c) zeta potential, d) swimming motility, and e) twitching motility for the clinical isolates and reference strains used in this study. Bars represent averages and error bars standard deviation of: for hydrophobicity and hydrodynamic diameter at least three biological replicas with two additional technical replicas, for motility three biological replica, for hydrophobicity three biological replicas.

Characterisation of urase activity and bacterial growth on culture plates

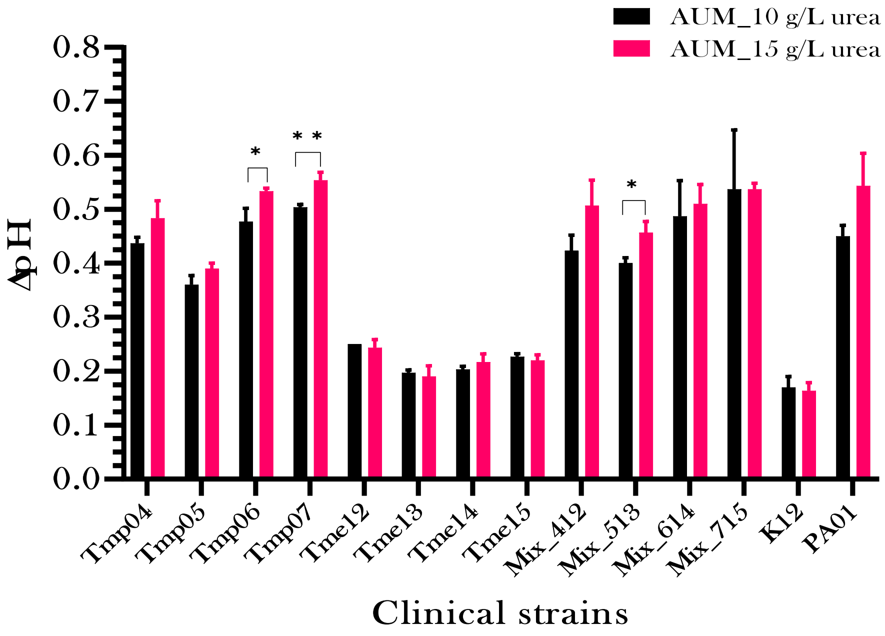

Figure S2. Change of pH in AUM.

Measurement of pH at the end point 30 h of bacterial cultivation. Black bars represent the pH change of bacterial samples in AUM with 10 g/L of urea in comparison to AUM without additional urea, red bars— pH change in AUM with 15 g/L of urea in comparison to AUM without additional urea. Data are presented by bars as mean ± standard deviation obtained from 3 replicates per sample (Statistical significance was represented as * when $p \leq 0.05$, ** — $p \leq 0.01$ after performing a two-tailed unpaired t-test). On the graph samples Mix_412, Mix_513, Mix_614 and Mix_715 correspond to Pair412, Pair513, Pair614 and Pair715 [illegible]

Table S1. Morphological description and enzymatic activity of CAUTI–strains

Visual evaluation of strain properties has been made by using a scale '1–5', where 'V' represents the highest feature, 'I' — the lowest, '0' — no feature, while 'yes' — the presence of feature, 'no' — the absence of feature, 'N/A' — absence of data. Colony colour was observed on blood agar plates.

	Clinical strains								Control strains	
	Tmp4	*Tmp5*	*Tmp6*	*Tmp7*	*Tme12*	*Tme13*	*Tme14*	*Tme15*	*K12*	*PA01*
**Haemolytic activity**	V	V	III	V	0,I	0,I	I,II	I	0	N/A
**Pyocyanin**	III	II	I	IV	0	0	0	0	0	V
**Medium colour change**	yes	yes	yes	yes	yes	yes	no	no	yes	yes
**Bacteriophage infection**	no	yes	yes	no	no	no	no	no	no	no
**Colony colour**	creamy	creamy with brown and blue	creamy	creamy with brown and blue	light brown	light brown	white	white	creamy	creamy with brown and blue
**Colony type**	dry	dry	normal	normal	wet	wet	wet	wet	wet	normal
**Urease activity**	II	IV	IV	V	0	0	0	0	0	V
**Lactase**	no	no	no	no	no	yes	yes	yes	N/A	no
**β-galactosidase**	no	no	no	no	no	yes	yes	yes	no	no
**β-glucosidase**	no	no	no	no	no	no	no	no	no	no
**Tryptophan deaminase**	yes	no	no	no	no	no	no	no	no	no

To investigate enzymatic activity differences, bacteria were cultured on various clinical media, including BA, UTI, and CLED solid media plates, and urease activity was monitored in liquid culture (**Table S1**). Clinical *P. aeruginosa* strains (Tmp04, Tmp05, Tmp06, Tmp07) had phenotypes similar to the reference strain PA01. However, Tmp04, differed in colony colour, odour and colony morphology. *E. coli* strains Tme12 and Tme13 had morphological similarities to the reference K12 strain, while Tme14 and Tme15 were different in colony colour and morphology. On UTI plates colonies of Tme13, Tme14 and Tme15 appeared purple, whereas Tme12 and K12 colonies were colourless (**Figure S3**).

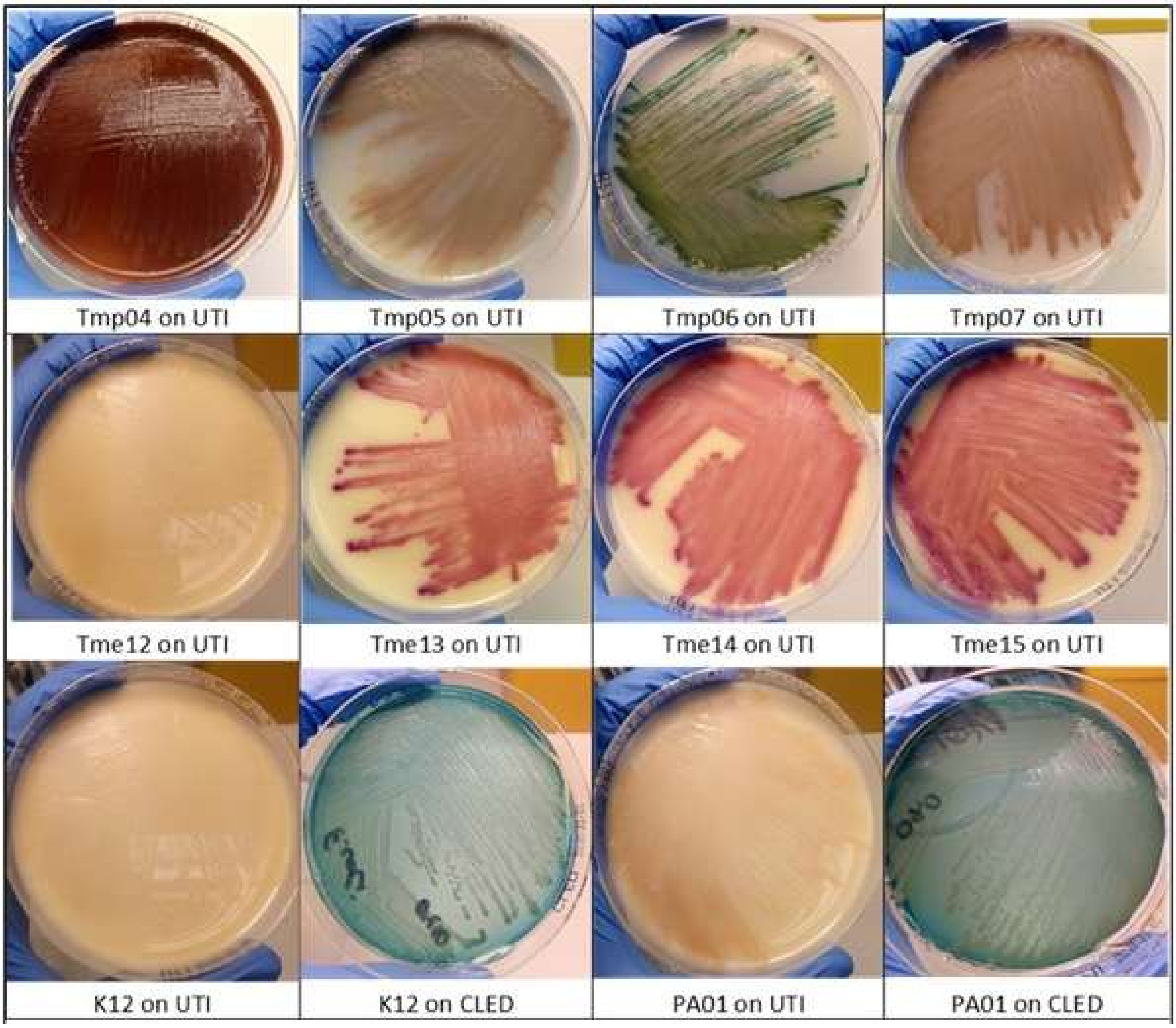

Figure S3. Growth of CAUTI–strains on UTI plates.

Colonies of *E. coli* strains were normally coloured pink due to β-galactosidase, but were colourless in the absence of this enzyme. Colonies of *P. aeruginosa* strains normally were colourless or had greenish and/or brownish shades. UTI–plates were the best for cultivating mixed cultures due to the colour difference between strains. The pair from K12 and PA01, as well as Tmp04 and Tme12 were differentiated based on their morphological differences.

Multivariate data analysis and plots

Metabolite profiles in ISO medium

The PCA model of supernatants from cultures grown in ISO showed distinct clustering where *E. coli* samples were tightly grouped, while *P. aeruginosa* samples were more dispersed. Cultures containing *E. coli* were separated along the first principal component, whereas those containing *P. aeruginosa* were separated along the second (**Figure S4**). Notably, supernatants from one isolate, Tmp05, were positioned close to ISO control, suggesting minimal bacterial growth in those samples.

The PCA model for planktonic and biofilm cells showed separation between *E. coli* and *P. aeruginosa* monocultures along the first principal component, with co-cultures approximately positioned between them. Planktonic and biofilm samples were further separated along the second component for both *E. coli* and *P. aeruginosa* strains (**Figure S5**). To better investigate the separation between classes, individual OPLS-DA models were built for supernatants (**Figure S6**), planktonic cells (**Figure S7**) and biofilm cell samples (**Figure S8**). Furthermore, SUS plots were generated to identify metabolites that differentiated sample groups. (**Figure S10–11)**. These plots represented the model loadings, comparing *P. aeruginosa* monocultures to co-cultures on the X-axis, and *E. coli* monocultures to co-cultures on the Y-axis.

Metabolite profiles in AUM

Following our analysis of metabolite patterns in a nutrient-rich ISO medium, we studied the nutrient-poor AUM medium, which mimics human urine in nutrient composition. PCA analysis of supernatants (**Figure S12**) showed separation along the first component, primarily reflecting the differences between the pure AUM medium and bacterial supernatants. Additionally, distinct groupings along the second principal component were observed, where *E. coli* samples were separated from *P. aeruginosa* samples and co-cultures placed between both monocultures.

In planktonic and biofilm cells, the PCA plot showed a clear separation between *E. coli, P. aeruginosa* monocultures, and co-cultures, with the latter ones, grouped closer to *P. aeruginosa* samples than *E. coli* samples (**Figure S13**). OPLS-DA models were thereafter built for supernatants (**Figure S14**), planktonic (**Figure S15**) and biofilm cells (**Figure S16**).

Additionally, SUS plots (**Figure S18**, **Figure S17** & **Figure S19**) were built, highlighting the key metabolites associated with mono- and co-cultures.

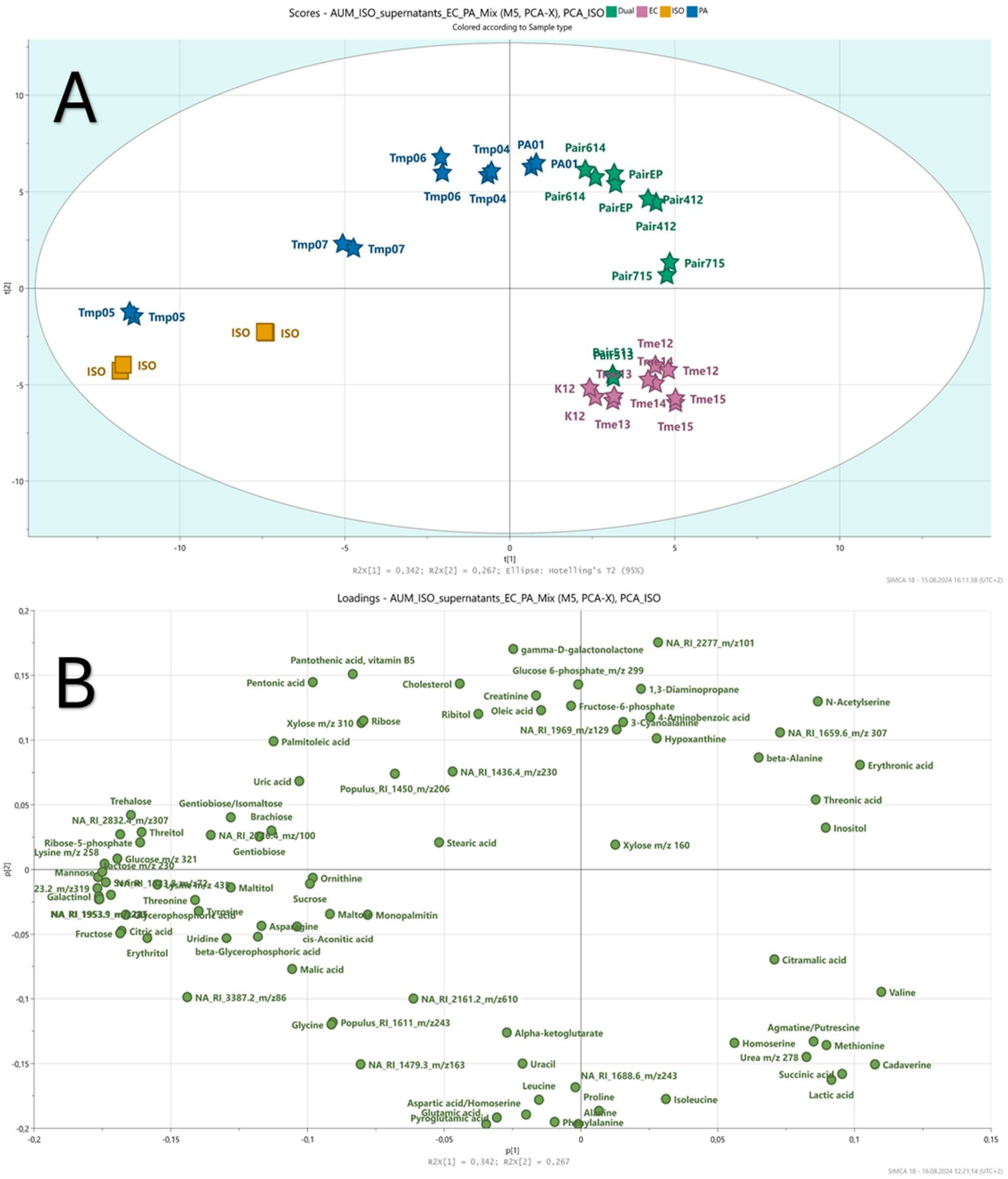

Figure S4. PCA model (R2X = 0.876, Q2 = 0.624) of supernatants in ISO.

A — Score plot; B — Loadings.

<u>Legend of score plot:</u> green stars (Dual) — supernatant co-culture, blue stars (PA) — supernatants of *P. aeruginosa* monocultures, pink stars (EC) — supernatants of *E. coli* monoculture, orange squares (ISO) — pure ISO medium

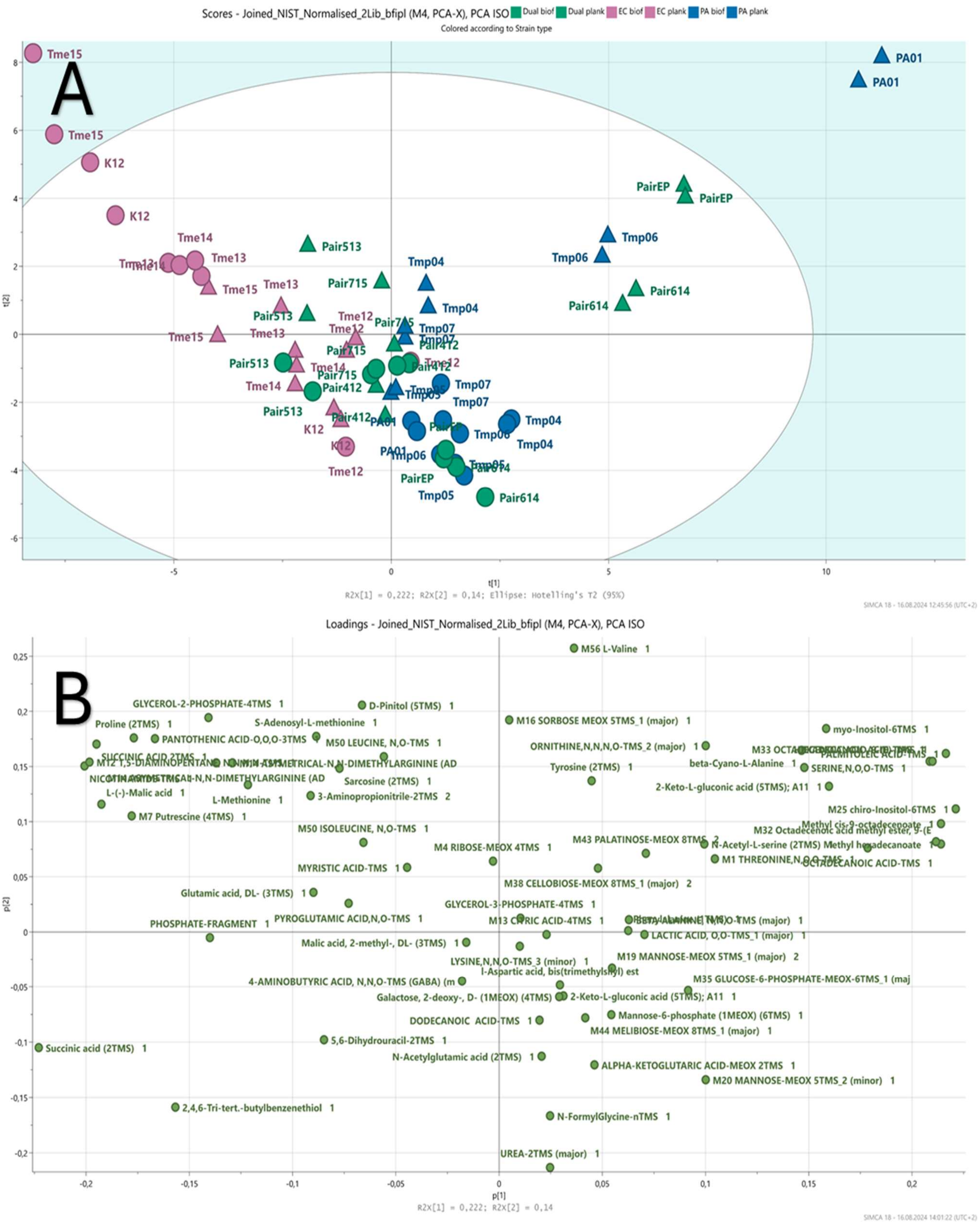

Figure S5. PCA model (R2X = 0.623, Q2 = 0.383) of planktonic and biofilm cells in ISO.

A — Score plot; B — Loadings.

Legend of score plot: green triangles (Dual plank) — planktonic cell co-culture, green circles (Dual biof) — biofilm cell co-culture, blue triangles (PA plank) — *P. aeruginosa* planktonic monocultures, blue circles (PA biof) — *P. aeruginosa* biofilm monocultures, pink triangles (EC plank) — *E. coli* planktonic monocultures, pink circles (EC plank) — *E. coli* biofilm monocultures

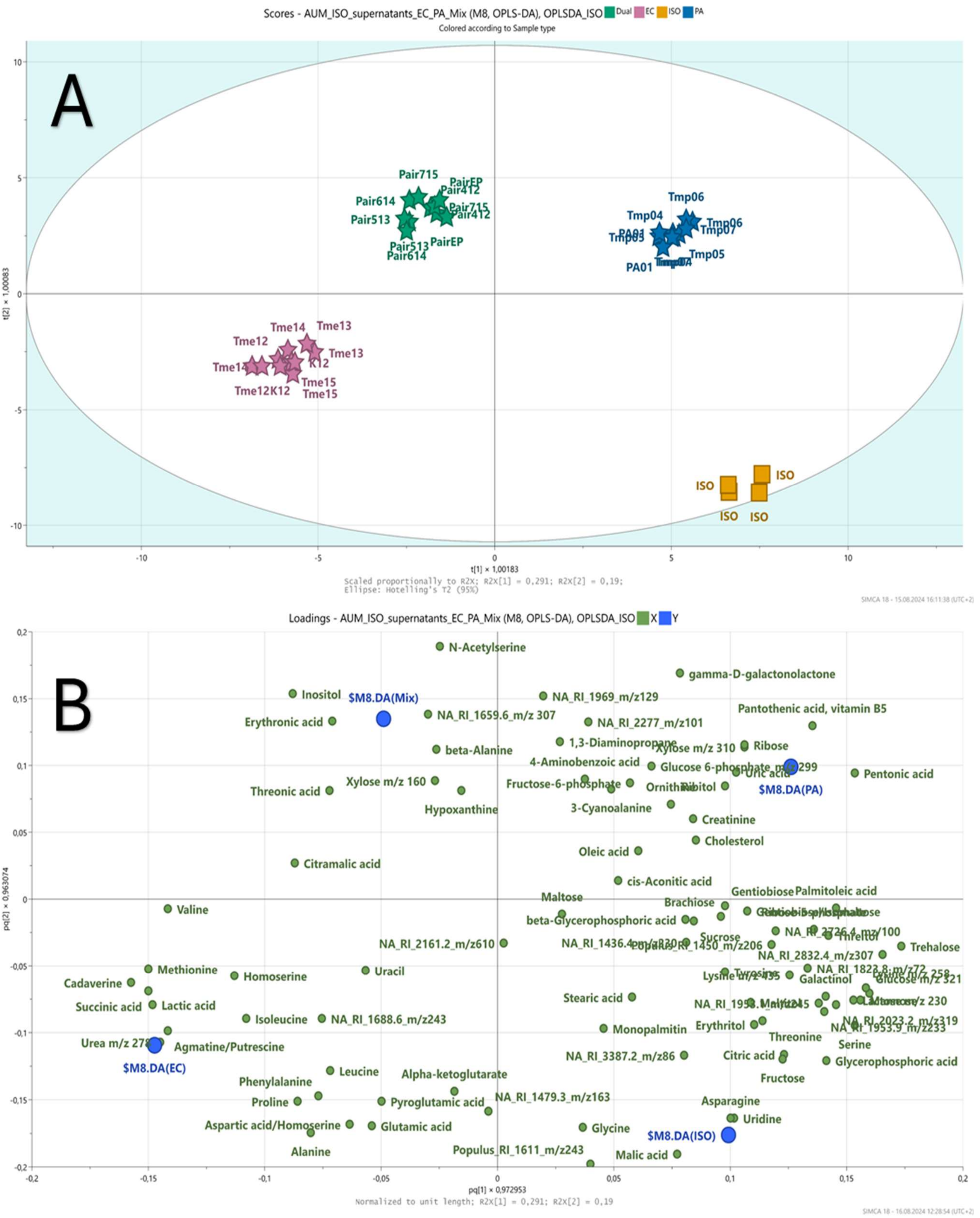

Figure S6. OPLS-DA model (R2X = 0.877, R2Y = 0.987, Q2 = 0.952, p = 5.57996 × 10^{-24}) of supernatants in ISO.

A — Score plot; B — Loadings.

Legend of score plot: green stars (Dual) — supernatant co-culture, blue stars (PA) — supernatants of *P. aeruginosa* monocultures, pink stars (EC) — supernatants of *E. coli* monoculture, orange squares (ISO) — pure ISO medium

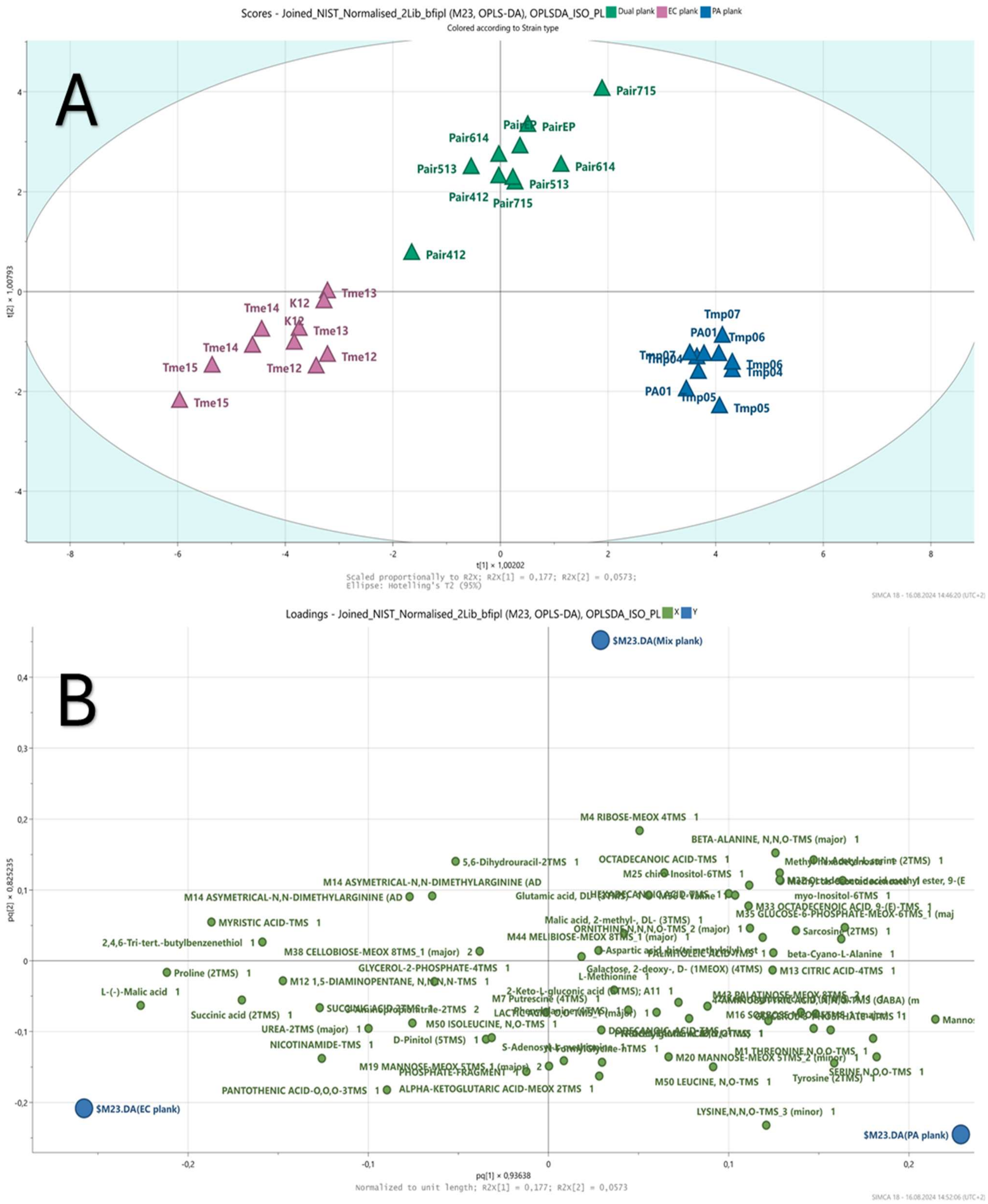

Figure S7 OPLS-DA model (R2X = 0.732, R2Y = 0.921, Q2 = 0.725, p = 1.41734 × 10^{-7}) of planktonic cells in ISO.

A — Score plot; B — Loadings.

Legend of score plot: green triangles (Dual plank) — planktonic cell co-culture, blue triangles (PA plank) — *P. aeruginosa* planktonic monocultures, pink triangles (EC plank) — *E. coli* planktonic monocultures

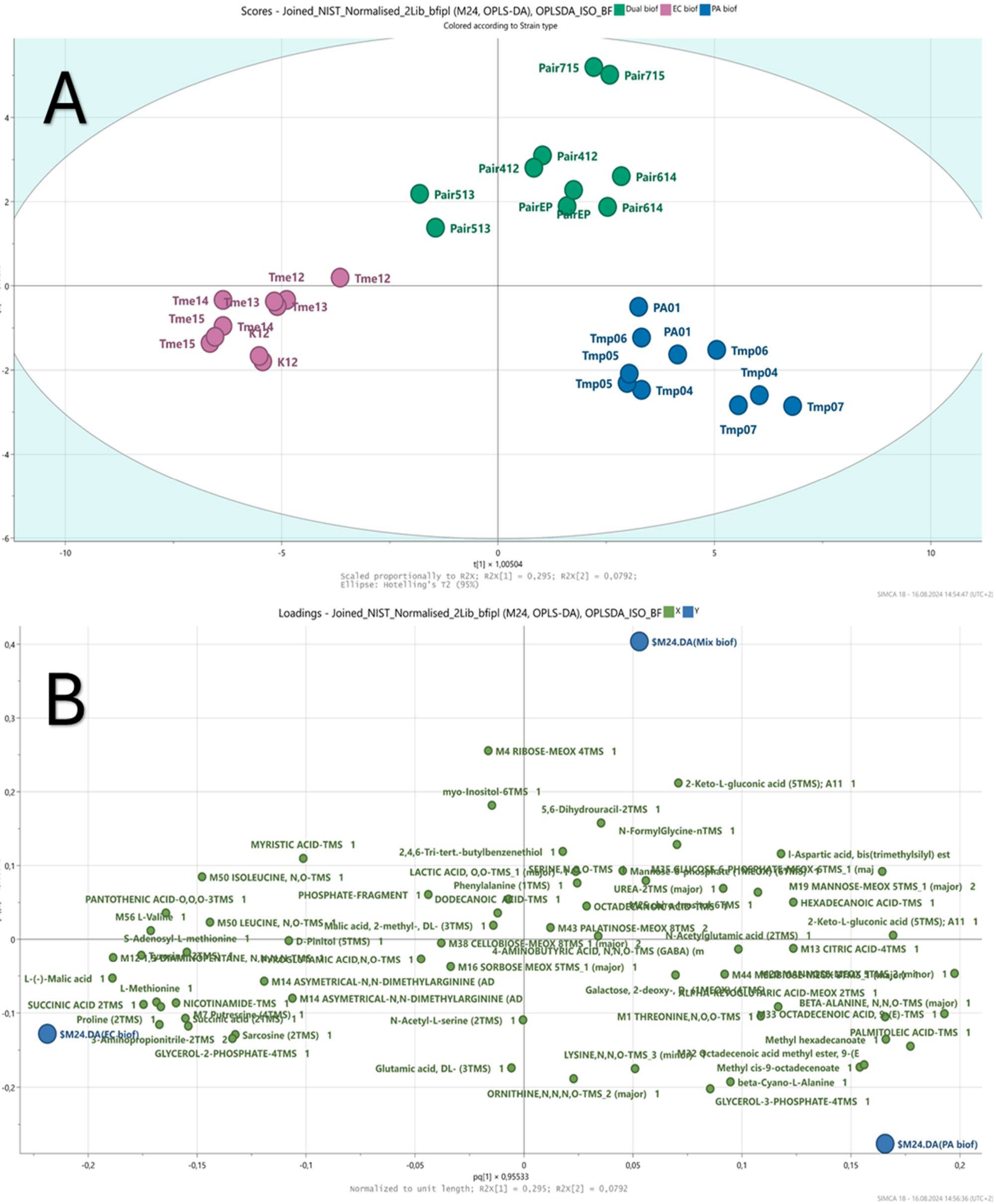

Figure S8. OPLS-DA model (R2X = 0.712, R2Y = 0.875, Q2 = 0.666, p = 1.43168 × 10^{-5}) of biofilm cells in ISO.

A — Score plot; B — Loadings.

<u>Legend of score plot:</u> green circles (Dual biof) — biofilm cell co-culture, blue circles (PA biof) — *P. aeruginosa* biofilm monocultures, pink circles (EC plank) — *E. coli* biofilm monocultures

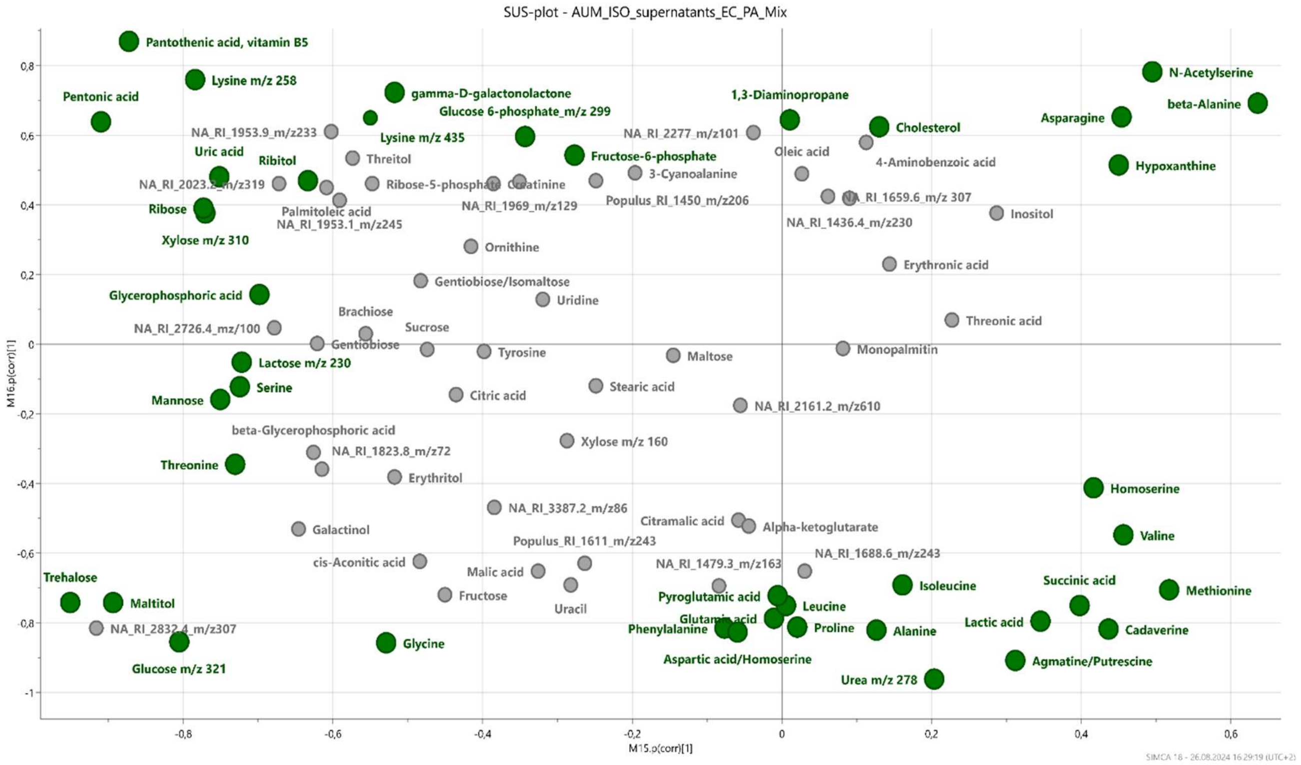

Figure S10. SUS-plot of supernatants in ISO.

OPLS-DA model of *P. aeruginosa* monocultures and co-cultures in ISO is on the X-axis, OPLS-DA model of *E. coli* monocultures and co-cultures in ISO is on the Y-axis. Annotated metabolites highlighted in green were picked for further analysis to check their levels in all samples.

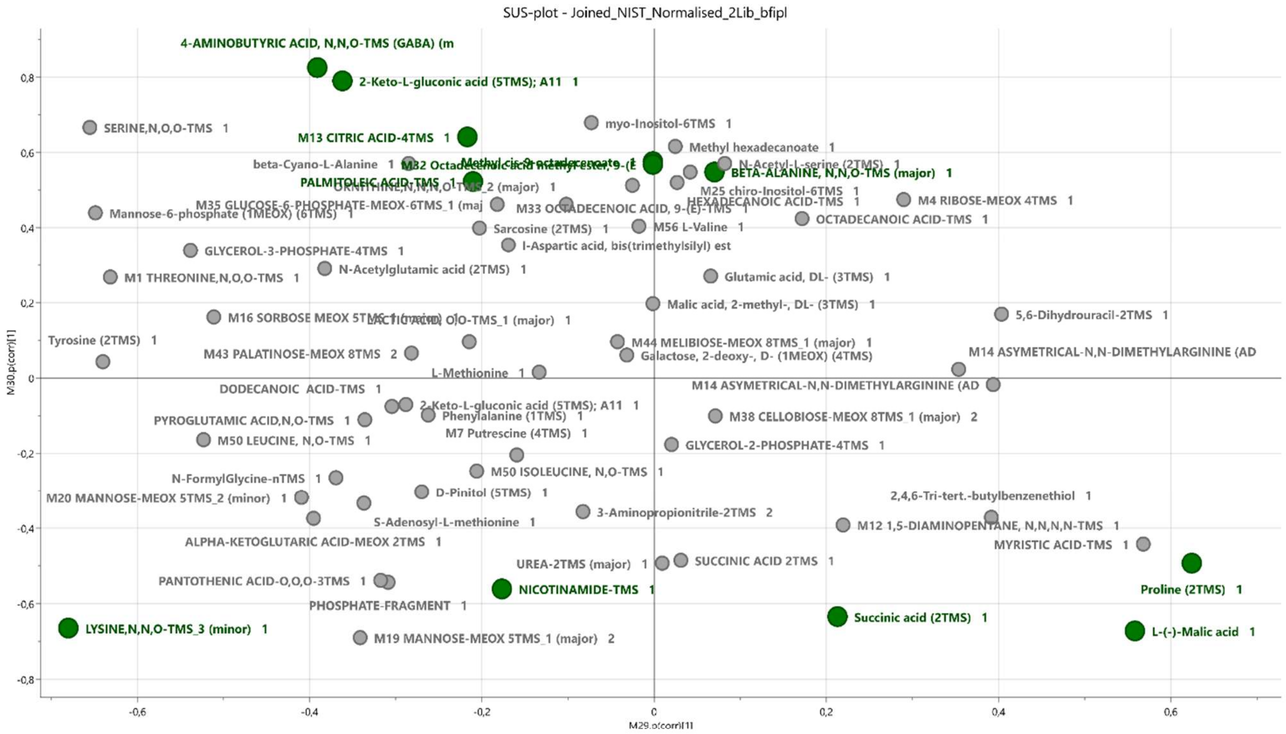

Figure S9. SUS-plot of planktonic cells in ISO.

OPLS-DA model of *P. aeruginosa* monocultures and co-cultures in ISO is on the X-axis, OPLS-DA model of *E. coli* monocultures and co-cultures in ISO is on the Y-axis. Annotated* metabolites highlighted in green were picked for further analysis to check their levels in all samples.

* — methyl cis-9-octadecenoic acid was further annotated as oleic acid

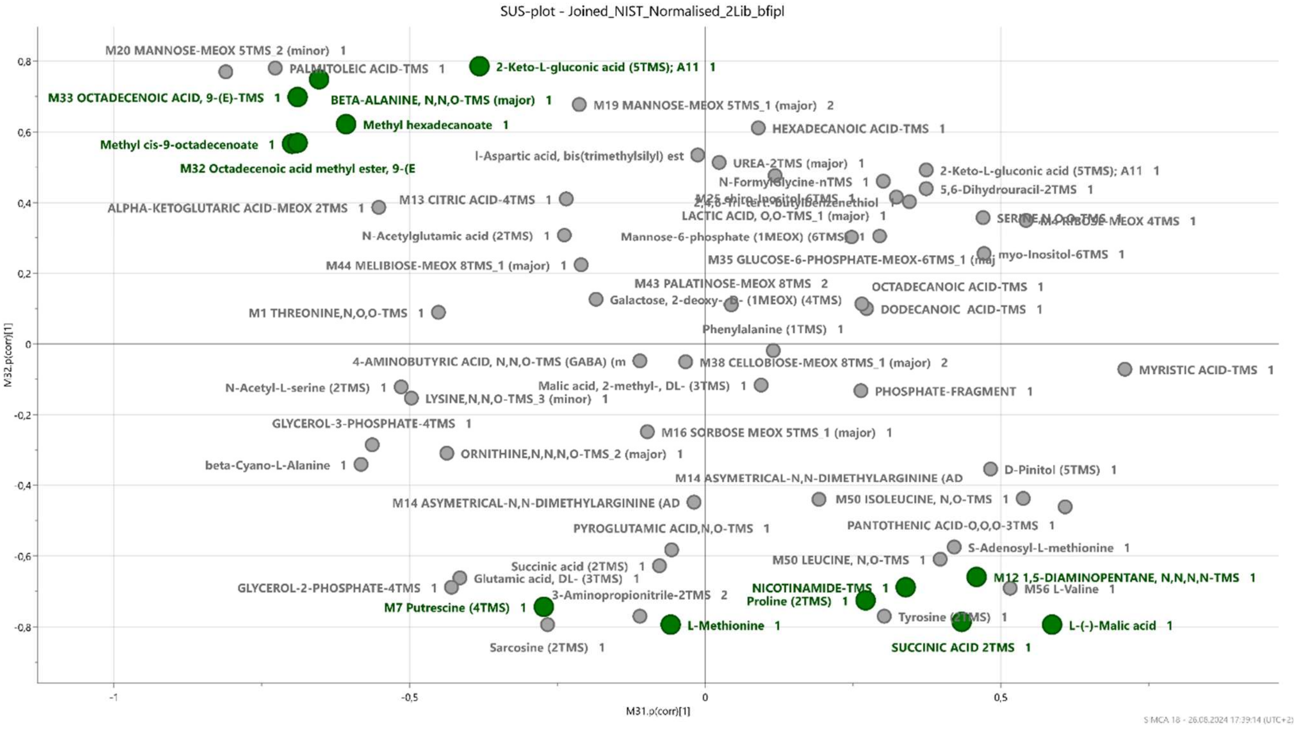

Figure S11. SUS-plot of biofilm cells in ISO.

OPLS-DA model of *P. aeruginosa* monocultures and co-cultures in ISO is on X-axis, OPLS-DA model of *E. coli* monocultures and co-cultures in ISO is on Y-axis. Annotated* metabolites highlighted in green were picked for further analysis to check their levels in all samples.

* — octadecenoic acid methyl ester was further annotated as methyl linoleate; octadecenoic acid was further annotated as oleic acid; methyl hexadecanoate was further annotated as methyl palmitate; putrescine was further annotated as agmatine/putrescine; 1,5-diaminopentane was further annotated as cadaverine

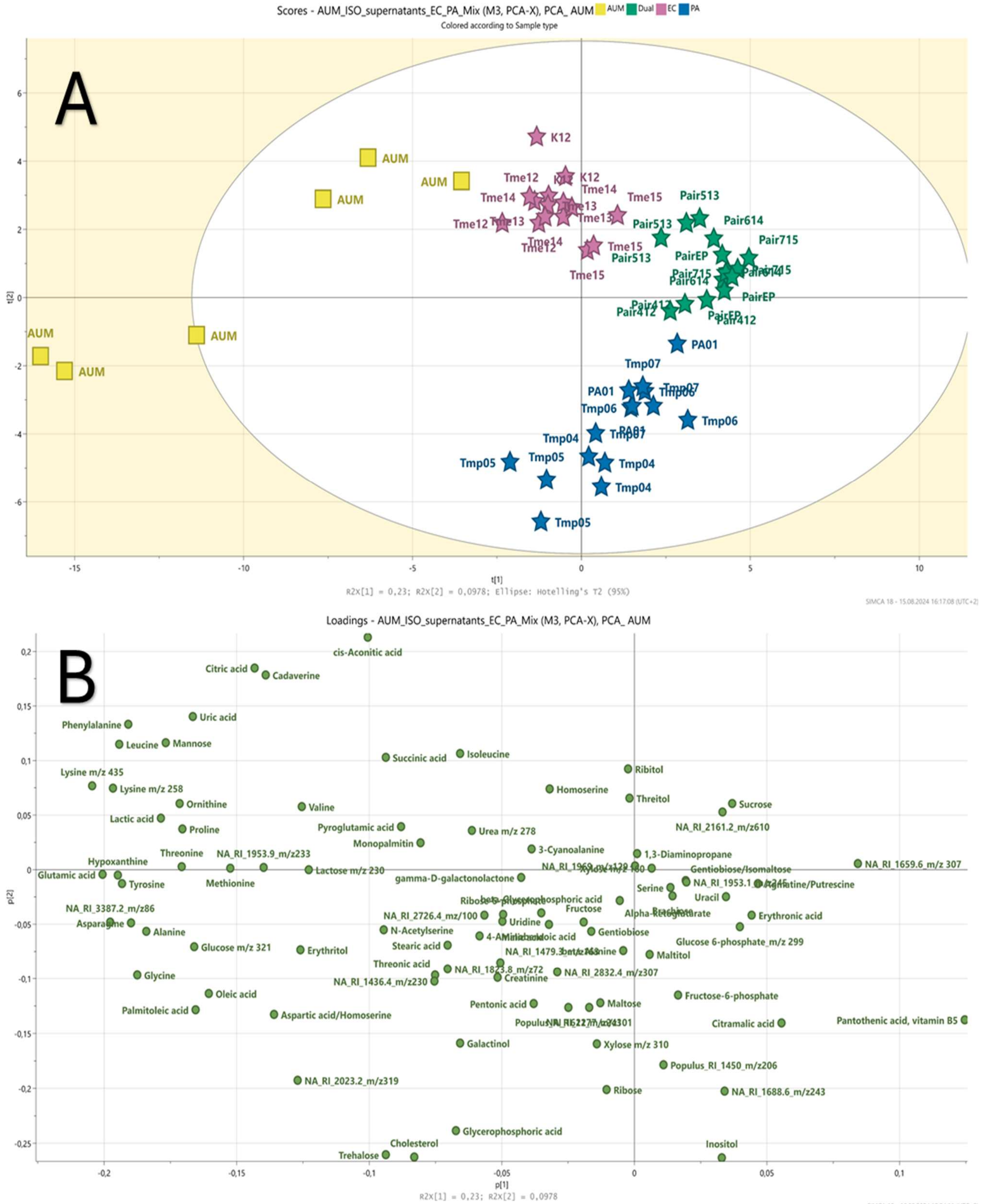

Figure S12. PCA model (R2X = 0.454, Q2 = 0.226) of supernatants in AUM.

A — Score plot; B — Loadings.

Legend of score plot: green stars (Dual) — supernatant co-culture, blue stars (PA) — supernatants of *P. aeruginosa* monocultures, pink stars (EC) — supernatants of *E. coli* monoculture, orange squares (AUM) — pure AUM medium

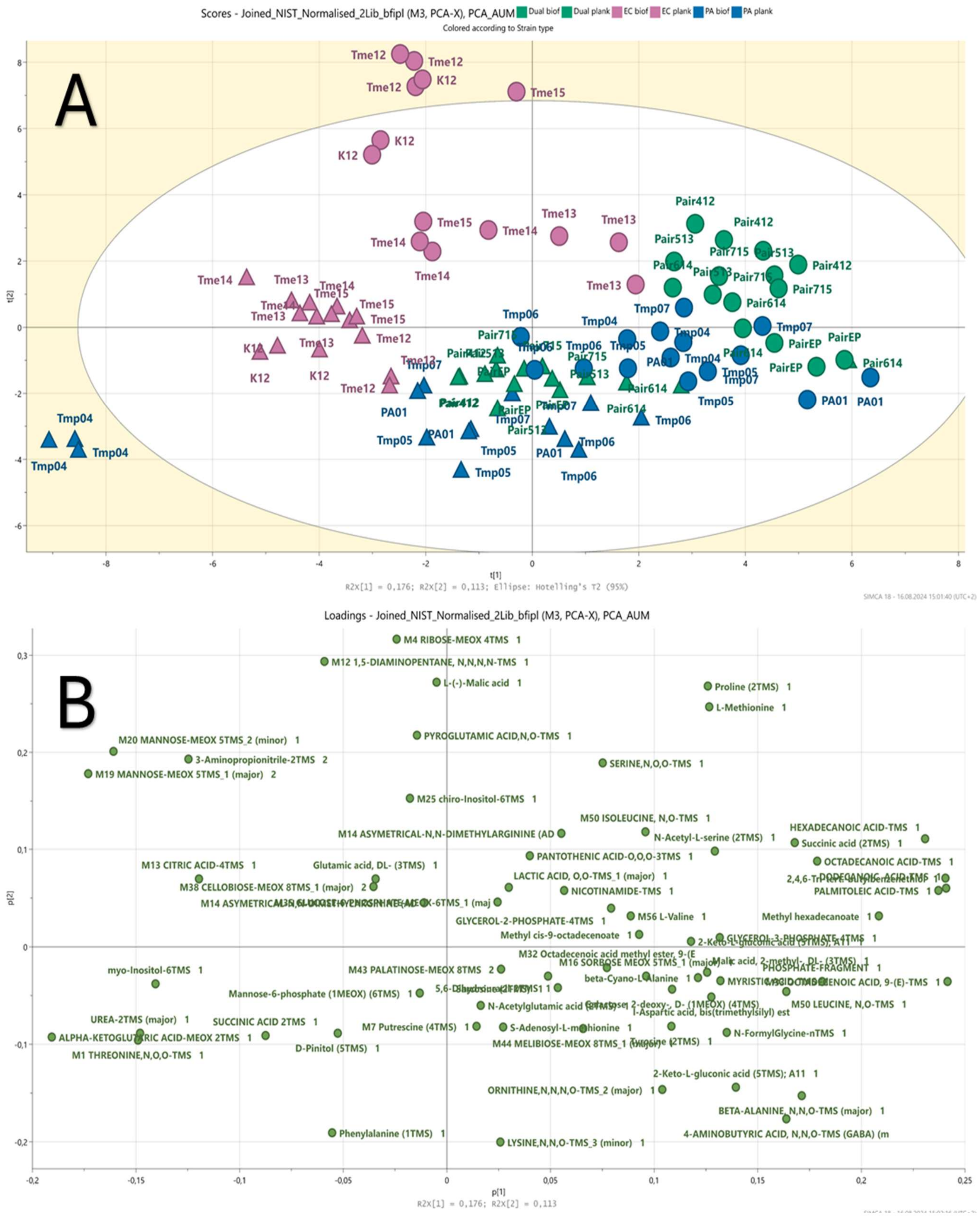

Figure S13. PCA model (R2X = 0.448, Q2 = 0.241) of planktonic and biofilm cells in AUM.

A — Score plot; B — Loadings.

<u>Legend of score plot:</u> green triangles (Dual plank) — planktonic cell co-culture, green circles (Dual biof) — biofilm cell co-culture, blue triangles (PA plank) — *P. aeruginosa* planktonic monocultures, blue circles (PA biof) — *P. aeruginosa* biofilm monocultures, pink triangles (EC plank) — *E. coli* planktonic monocultures, pink circles (EC plank) — *E. coli* biofilm monocultures

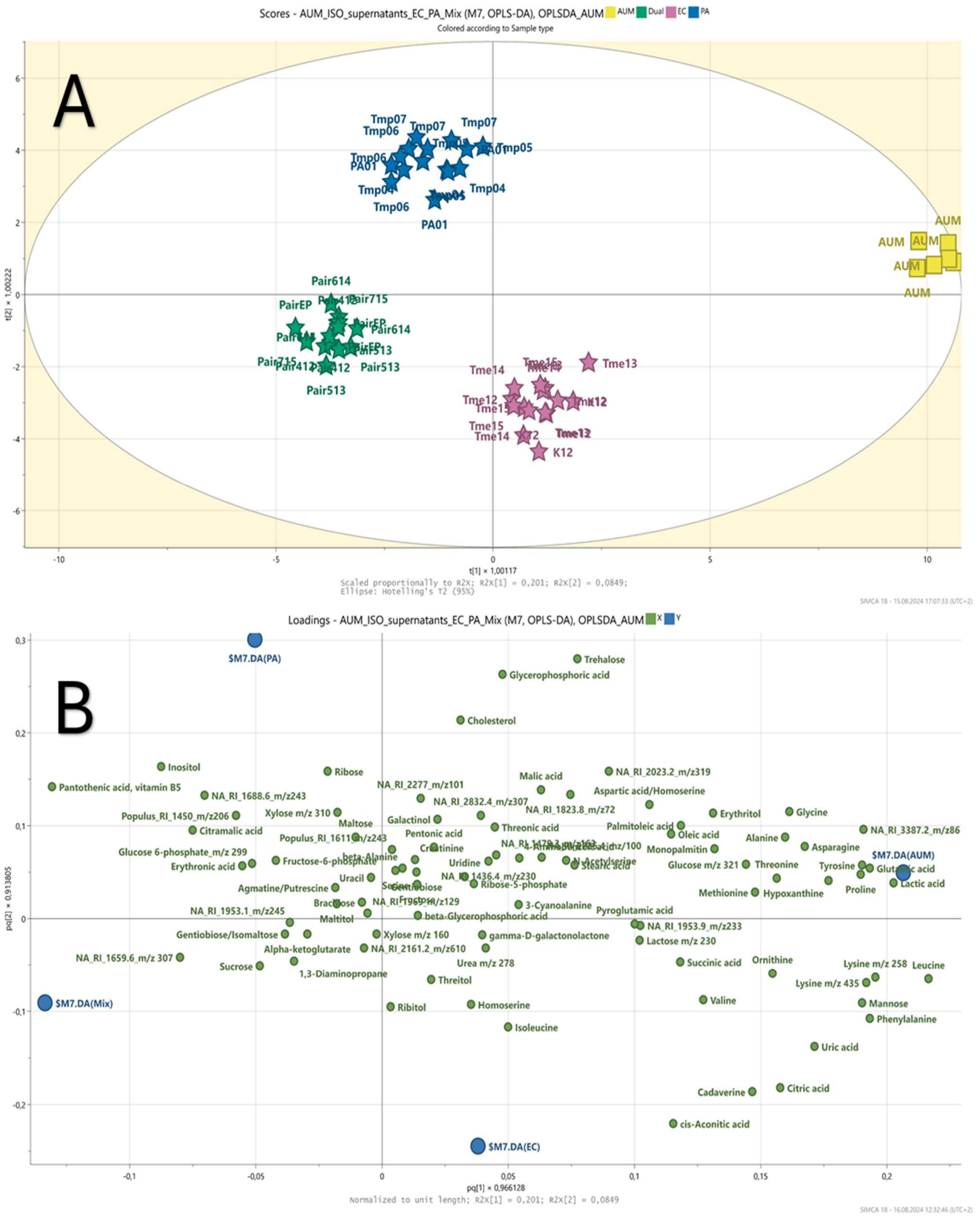

Figure S14. OPLS-DA model (R2X = 0.572, R2Y = 0.973, Q2 = 0.868, p = 2.89096 × 10^{-32}) of supernatants in AUM.

A — Score plot; B — Loadings.

Legend of score plot: green stars (Dual) — supernatant co-culture, blue stars (PA) — supernatants of *P. aeruginosa* monocultures, pink stars (EC) — supernatants of *E. coli* monoculture, orange squares (AUM) — pure AUM medium

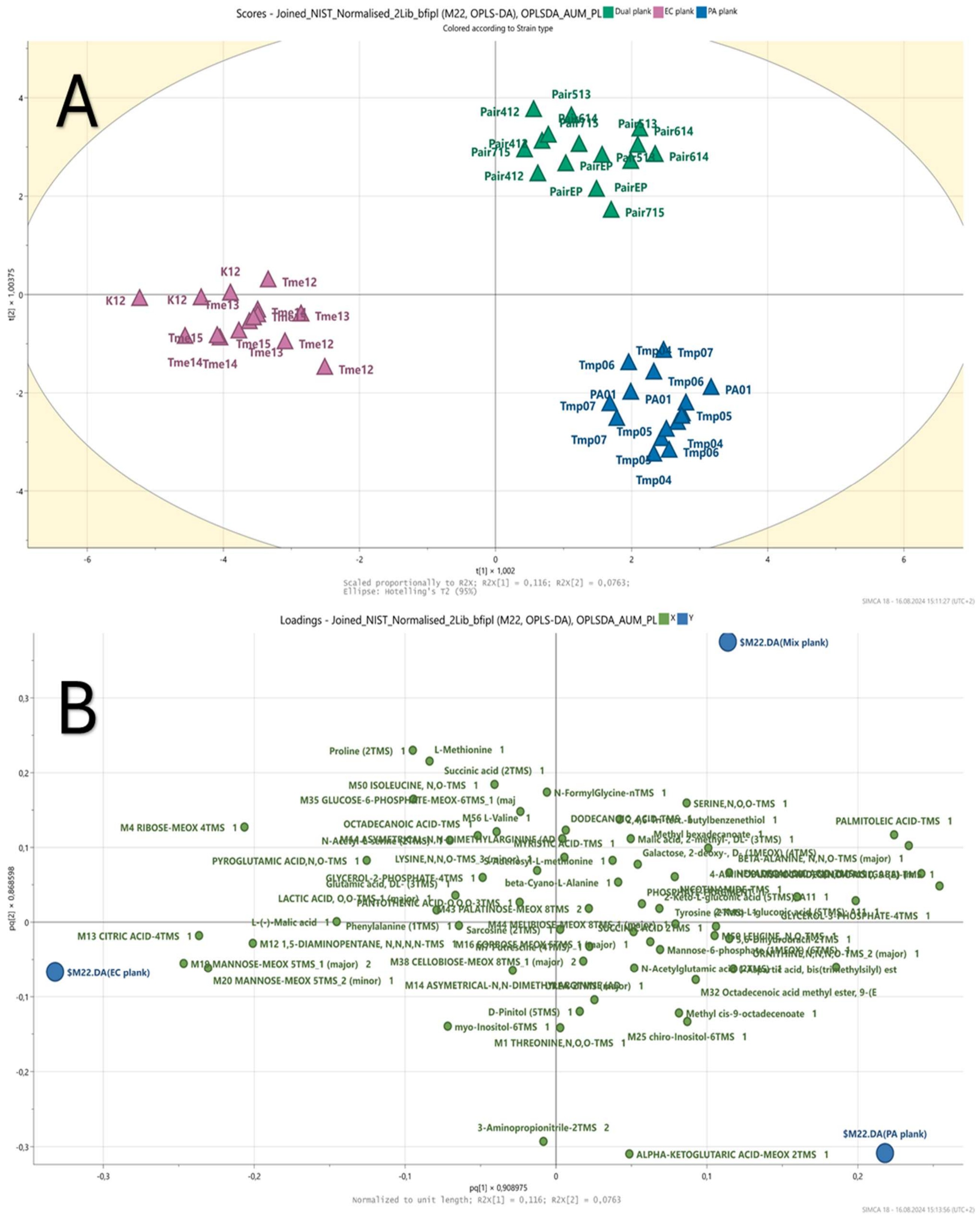

Figure S15. OPLS-DA model (R2X = 0.654, R2Y = 0.95, Q2 = 0.794, p = 1.11749 × 10^{-10}) of planktonic cells in AUM.

A — Score plot; B — Loadings.

Legend of score plot: green triangles (Dual plank) — planktonic cell co-culture, blue triangles (PA plank) — *P. aeruginosa* planktonic monocultures, pink triangles (EC plank) — *E. coli* planktonic monocultures

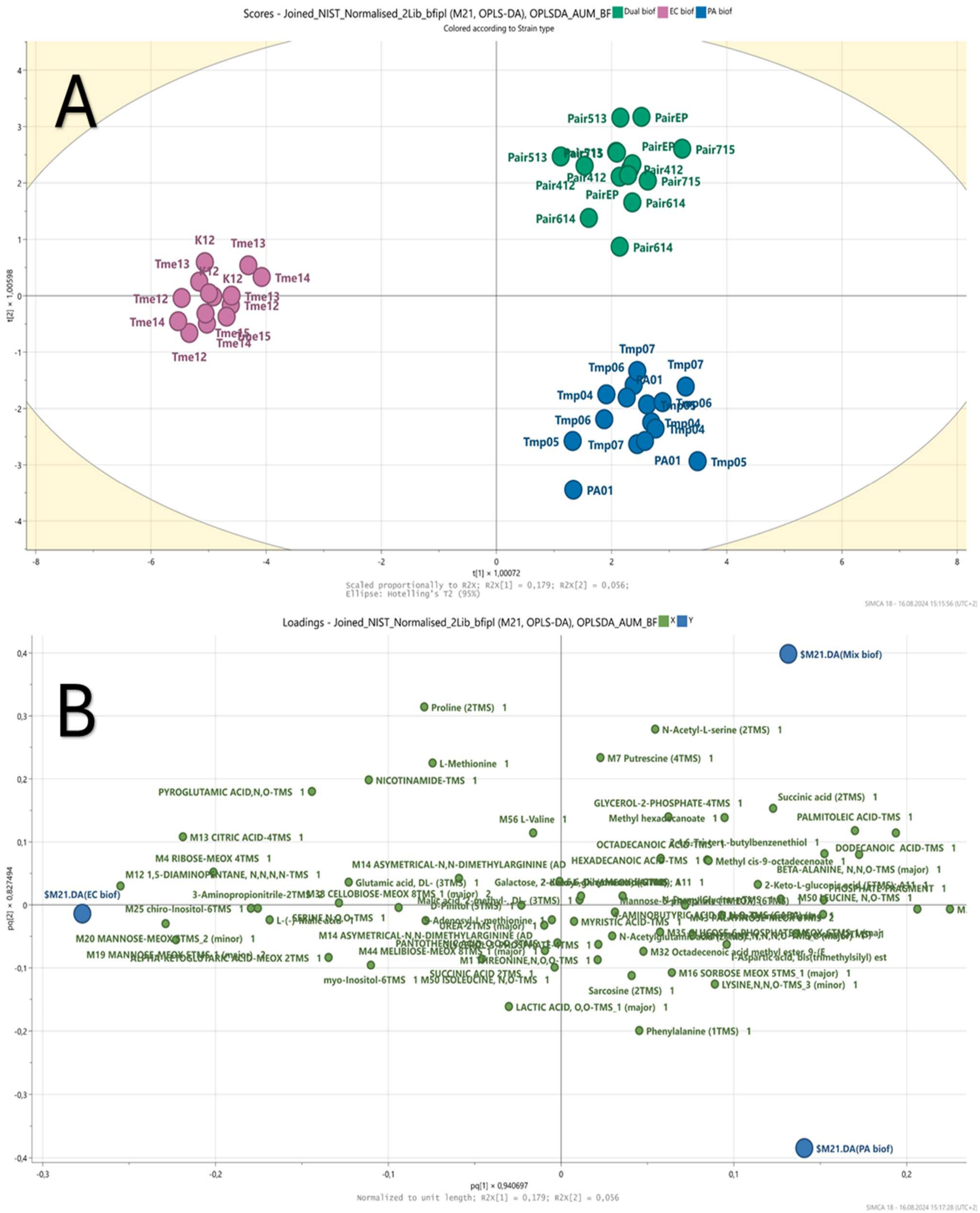

Figure S16. OPLS-DA model (R2X = 0.649, R2Y = 0.952, Q2 = 0.719, p = 8.20287 × 10^{-12}) of biofilm cells in ISO.

A — Score plot; B — Loadings.

Legend of score plot: green circles (Dual biof) — biofilm cell co-culture, blue circles (PA biof) — *P. aeruginosa* biofilm monocultures, pink circles (EC plank) — *E. coli* biofilm monocultures

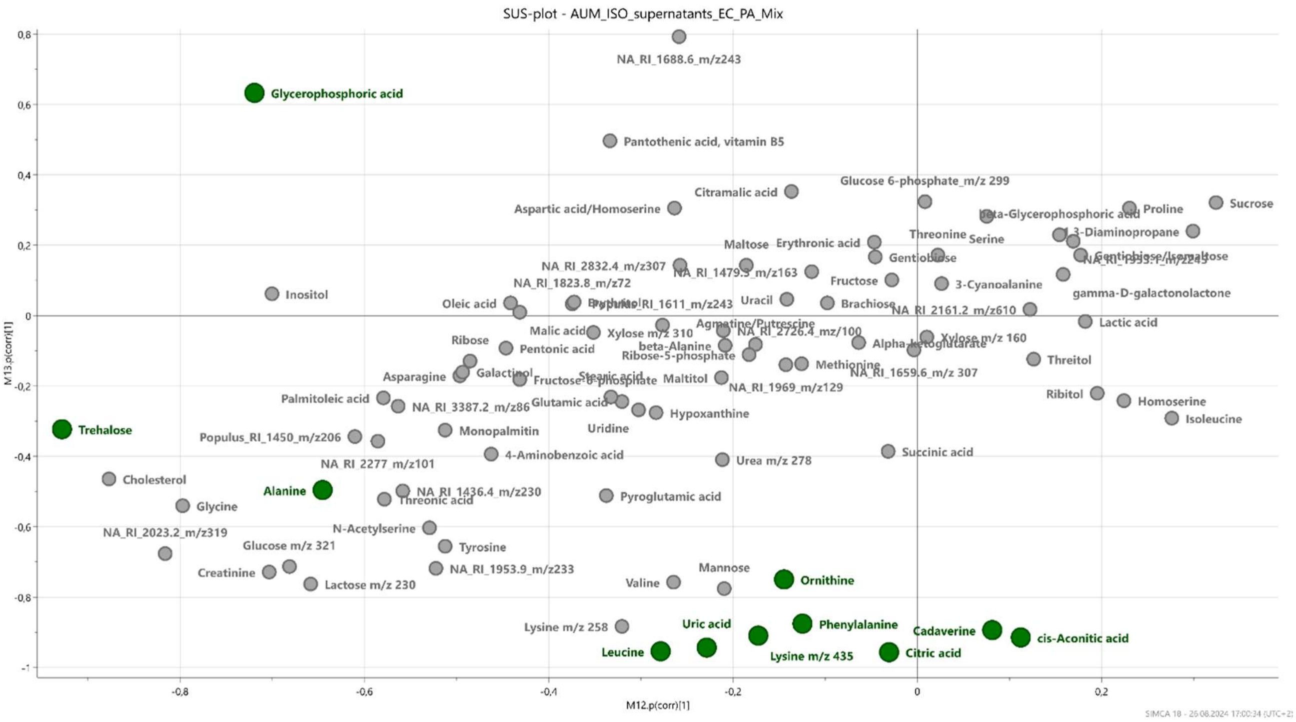

Figure S18. SUS-plot of supernatants in AUM.

OPLS-DA model of *P. aeruginosa* monocultures and co-cultures in AUM is on X-axis, OPLS-DA model of *E. coli* monocultures and co-cultures in AUM is on Y-axis. Annotated metabolites highlighted in green were picked for further analysis to check their levels in all samples

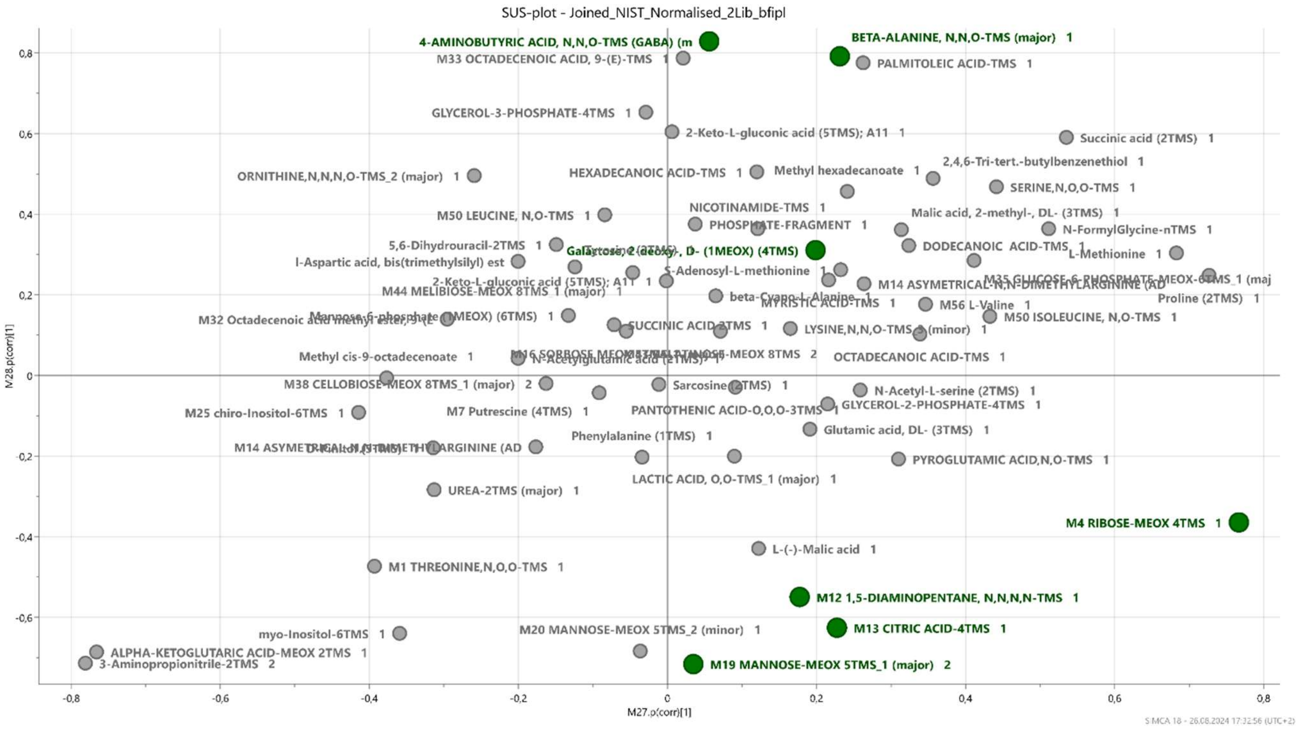

Figure S17. SUS-plot of planktonic cells in AUM.

OPLS-DA model of *P. aeruginosa* monocultures and co-cultures in AUM is on X-axis, OPLS-DA model of *E. coli* monocultures and co-cultures in AUM is on Y-axis. Annotated* metabolites highlighted in green were picked for further analysis to check their levels in all samples

* — 1,5-diaminopentane was further annotated as cadaverine

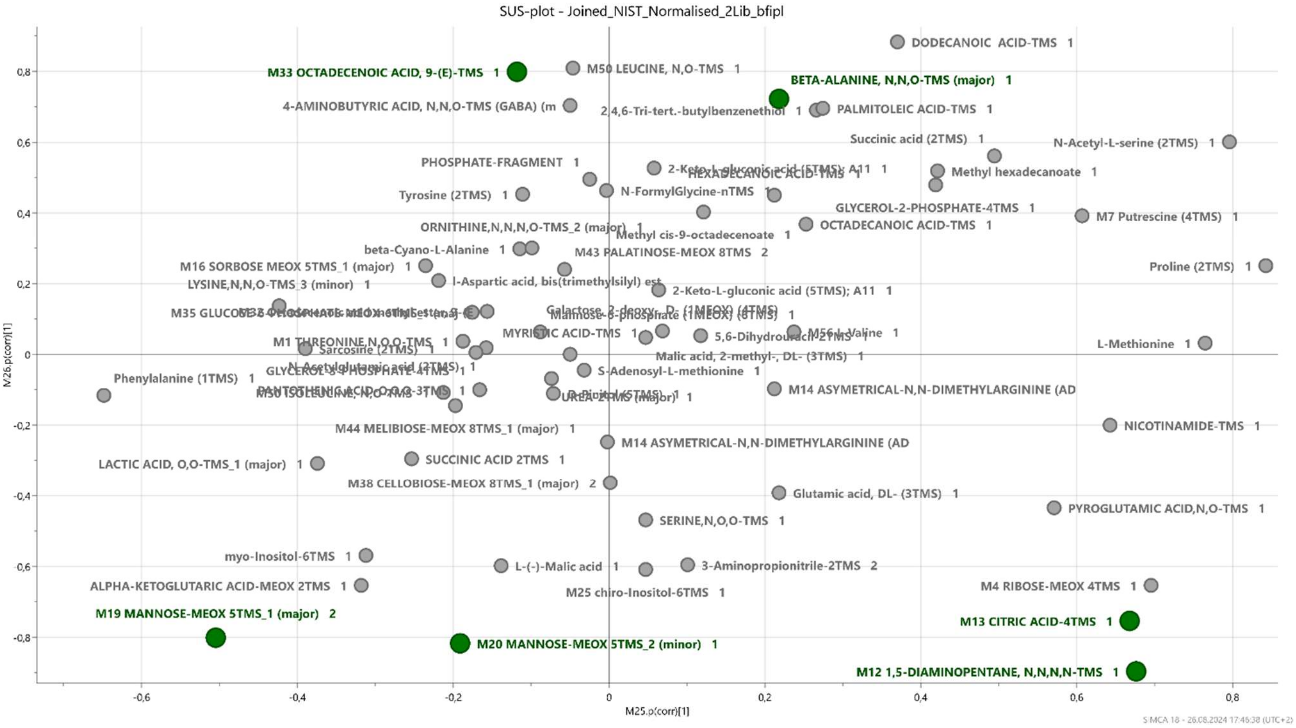

Figure S19. SUS-plot of biofilm cells in AUM.

OPLS-DA model of *P. aeruginosa* monocultures and co-cultures in AUM is on X-axis, OPLS-DA model of *E. coli* monocultures and co-cultures in AUM is on Y-axis. Annotated* metabolites highlighted in green were picked for further analysis to check their levels in all samples

* — octadecenoic acid was further annotated as oleic acid; 1,5-diaminopentane was further annotated as cadaverine; M20 mannose (minor) was further annotated as galactose; M19 mannose (major) was further annotated as mannose

Urease activity

The urease activity of CAUTI strains was evaluated based on the colour change of test tubes (**Figure S20**). CAUTI strains of *E. coli* showed no urease activity, consistent with previous literature[8]. Among the *P. aeruginosa* strains, Tmp05, Tmp06, and Tmp07 showed urease activity comparable to the reference strain PA01(**Figure S20**). Co-cultures showed differing urease activity compared to their respective monocultures. Pair412 and Pair513 had no urease activity after 120 h of incubation, while Pair715 had urease levels similar to PairEP. In contrast, Pair614 showed reduced urease activity compared to Tmp06.

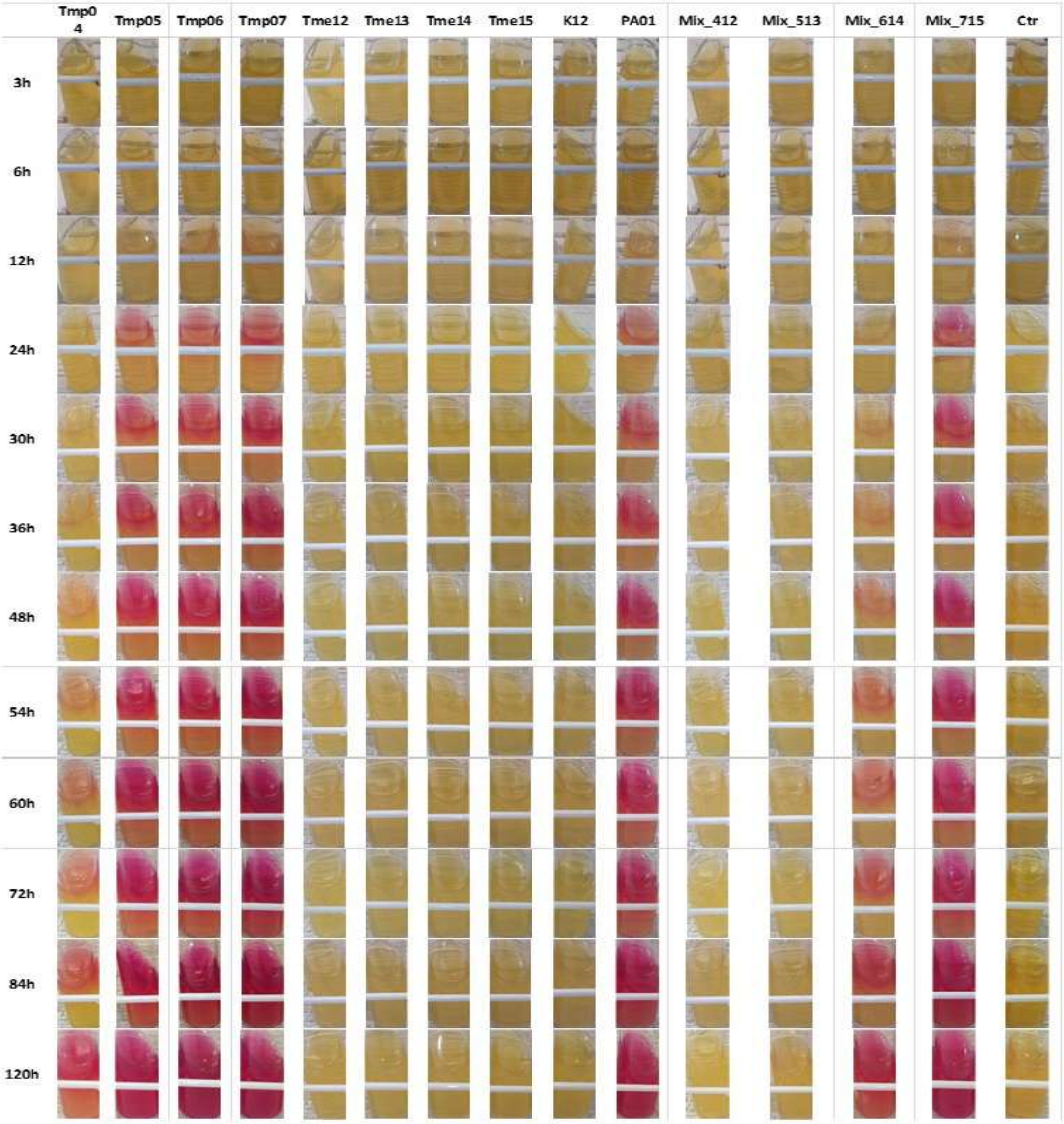

Figure S20. Urease test of CAUTI–strains.

Tubes with urease-positive bacteria changed their colour due to alkalinisation of the medium, i.e. indicator phenol red turned from yellow to pink within 24 h. Tubes with bacteria with weak urease activity became pink within few days. Tubes with bacteria without urease activity remained yellow.

Growth of strains in 96-well plates

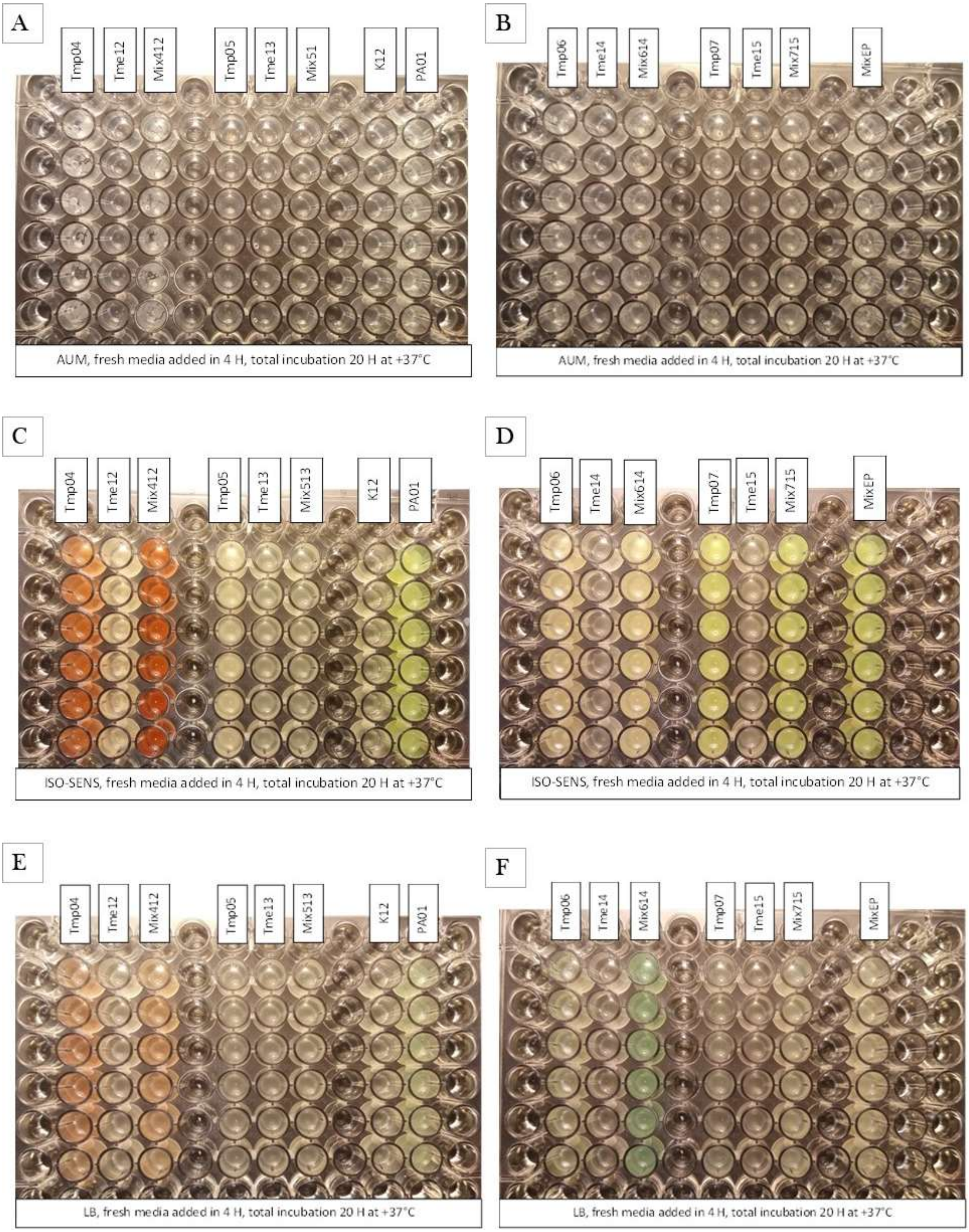

Figure S21. Growth of CAUTI–strains in different media in 96-well plates.

A, B — mono- and dual-species biofilms in AUM; C, D — mono- and dual-species biofilms in ISO; E, F — mono- and dual-species biofilms in LB

References

1. Rosenberg, M., Gutnick, D. & Rosenberg, E. Adherence of bacteria to hydrocarbons: A simple method for measuring cell-surface hydrophobicity. *FEMS Microbiology Letters* **9**, 29–33 (1980).
2. Burkart, M., Toguchi, A. & Harshey, R. M. The chemotaxis system, but not chemotaxis, is essential for swarming motility in Escherichia coli. *Proceedings of the National Academy of Sciences* **95**, 2568–2573 (1998).
3. Ha, D.-G., Kuchma, S. L. & O'Toole, G. A. Plate-Based Assay for Swimming Motility in Pseudomonas aeruginosa. in *Pseudomonas Methods and Protocols* (eds Filloux, A. & Ramos, J.-L.) 59–65 (Springer, New York, NY, 2014). doi:10.1007/978-1-4939-0473-0_7.
4. Ha, D.-G., Kuchma, S. L. & O'Toole, G. A. Plate-Based Assay for Swarming Motility in Pseudomonas aeruginosa. in *Pseudomonas Methods and Protocols* (eds Filloux, A. & Ramos, J.-L.) 67–72 (Springer, New York, NY, 2014). doi:10.1007/978-1-4939-0473-0_8.
5. Tremblay, J. & Déziel, E. Improving the reproducibility of Pseudomonas aeruginosa swarming motility assays. *J Basic Microbiol* **48**, 509–515 (2008).
6. Xicohtencatl-Cortes, J. *et al.* The Type 4 Pili of Enterohemorrhagic Escherichia coli O157:H7 Are Multipurpose Structures with Pathogenic Attributes. *J Bacteriol* **191**, 411–421 (2009).
7. Turnbull, L. & Whitchurch, C. B. Motility Assay: Twitching Motility. in *Pseudomonas Methods and Protocols* (eds Filloux, A. & Ramos, J.-L.) 73–86 (Springer, New York, NY, 2014). doi:10.1007/978-1-4939-0473-0_9.
8. Bichler, K.-H. *et al.* Urinary infection stones. *International Journal of Antimicrobial Agents* **19**, 488–498 (2002).
9. Budhathoki-Uprety, J. *et al.* Synthetic molecular recognition nanosensor paint for microalbuminuria. *Nat Commun* **10**, 3605 (2019).
10. Nakayama, A. *et al.* Presence of immunounreactive albumin in the urine of diabetic patients. *Journal of Clinical Laboratory Analysis* **20**, 29–36 (2006).
11. Kida, Y., Shimizu, T. & Kuwano, K. Cooperation between LepA and PlcH Contributes to the In Vivo Virulence and Growth of Pseudomonas aeruginosa in Mice. *Infection and Immunity* **79**, 211–219 (2011).
12. Kruczek, C. *et al.* Serum albumin alters the expression of iron-controlled genes in Pseudomonas aeruginosa. *Microbiology* **158**, 353–367.
